# Pyranodipyran Derivatives with Tyrosyl DNA Phosphodiesterase 1 Inhibitory Activities and Fluorescent Properties from *Aspergillus* sp. EGF 15-0-3

**DOI:** 10.3390/md20030211

**Published:** 2022-03-17

**Authors:** Xia Wei, Fang-Ting Wang, Mei-Xia Si-Tu, Hao Fan, Jin-Shan Hu, Hao Yang, Shan-Yue Guan, Lin-Kun An, Cui-Xian Zhang

**Affiliations:** 1 School of Pharmaceutical Sciences, Guangzhou University of Chinese Medicine, Guangzhou 510006, China; weixia6994@163.com (X.W.); stmxlalala@163.com (M.-X.S.-T.); fh_tcm@163.com (H.F.); hujinshan_zoe@163.com (J.-S.H.); 2 School of Pharmaceutical Sciences, Sun Yat-Sen University, Guangzhou 510006, China; wangft5@mail2.sysu.edu.cn (F.-T.W.); yangh328@mail2.sysu.edu.cn (H.Y.); 3 Instrumental Analysis & Research Center, Sun Yat-Sen University, Guangzhou 510275, China; pusgshy@mail.sysu.edu.cn

**Keywords:** soft-coral-derived fungus, pyranodipyran derivatives, tyrosyl DNA phosphodiesterase 1 inhibitory activities, fluorescent properties

## Abstract

Four new benzodipyran racemates, namely (±)-aspergiletals A–D (**3**–**6**), representing a rare pyrano[4,3-*h*]chromene scaffold were isolated together with eurotiumide G (**1**) and eurotiumide F (**2**) from the soft-coral-derived fungus *Aspergillus* sp. EGF 15-0-3. All the corresponding optically pure enantiomers were successfully separated by a chiral HPLC column. The structures and configurations of all the compounds were elucidated based on the combination of NMR and HRESIMS data, chiral separation, single-crystal X-ray diffraction, quantum chemical ^13^C NMR, and electronic circular dichroism calculations. Meanwhile, the structure of eurotiumide G was also revised. The TDP1 inhibitor activities and photophysical properties of the obtained compounds were evaluated. In the TDP1 inhibition assay, as a result of synergy between (+)-**6** and (−)-**6**, (±)-**6** displayed strong inhibitory activity to TDP1 with IC_50_ values of 6.50 ± 0.73 μM. All compounds had a large Stokes shift and could be utilized for elucidating the mode of bioactivities by fluorescence imaging.

## 1. Introduction

Tyrosyl DNA phosphodiesterase 1 (TDP1) has recently been considered as a rational anticancer target [1,2] owning to its ability to break down various DNA adducts induced by chemotherapeutics drugs [3,4], such as topotecan, irinotecan, 10-hydroxycamptothecin, and belotecan [5,6]. TDP1 inhibitors can potentiate the combined anticancer activities of Top1-target anticancer drugs and overcome cancer cell resistance to therapeutic drugs in some cancers [7,8,9]. However, most TDP1 inhibitors today are synthetic compounds [10].

Benzodipyran is a small but unique class that mostly comes from chemical synthesis [11]. The biological activity of benzopyran scaffolds has gained considerable attention over the last few decades [12]. However, benzodipyran bearing a pyrano[4,3-*h*]chromene unit in its backbone is rare in nature, with only two examples reported to date [13].

**Figure 1:**
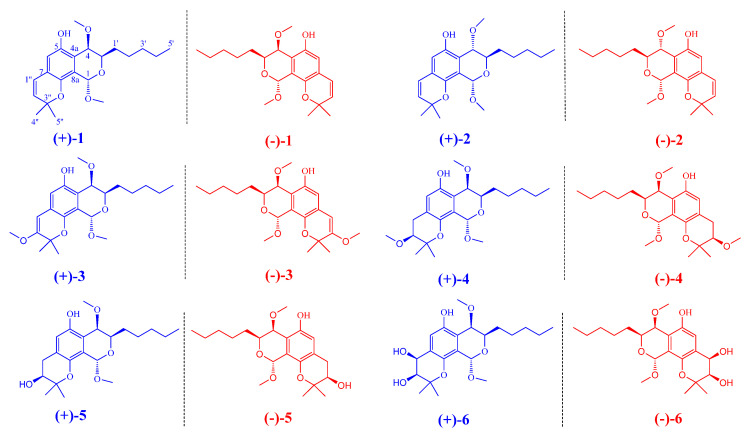
Chemical structures of compounds **1**−**6** from *Aspergillus* sp. EGF 15-0-3.

During our continuing search for natural TDP1 inhibitors [14,15,16,17,18], four new benzodipyran racemates, namely (±)-aspergiletals A–D (**3**–**6**), representing a rare pyrano[4,3-*h*]chromene scaffold were isolated together with eurotiumide G (**1**) and eurotiumide F (**2**) from the soft-coral-derived fungus *Aspergillus* sp. EGF 15-0-3 (Figure 1). Biogenetically, these rare scaffolds were formed by a cascade sequence of epoxidation, nucleophilic cyclization, and oxidation from benzene carbaldehydes. Structurally, **3**–**6** share the rare pyrano[4,3-*h*]chromene framework but differ in the oxygenation patterns on the 2H-pyran ring. Due to the high substitution of two pyran rings, the stereochemical complexity of these molecules makes it challenging to elucidate their absolute configurations. Based on the combination of NMR and HRESIMS data, chiral separation, quantum chemical ^13^C NMR, electronic circular dichroism calculations, and single-crystal X-ray diffraction, the absolute configurations of all the compounds were umambiguously determined. Meanwhile, the structure of eurotiumide G, was also revised. All compounds were screened for TDP1 inhibition, and the photophysical properties were evaluated. Herein, the isolation and structure elucidation, hypothetical biogenetic pathway, TDP1 inhibitor activities, and the photophysical properties of **1**–**6** are presented.

## 2. Results

Previously, the metabolite capability of the title strain was preliminarily accessed by integration of the OSMAC strategy and LC–MS/MS analyses [16]. As a result, the chemical profiles of benzodipyran were identified in four different media (Figure S1), indicating that benzodipyran was produced in 4 out of 18 tested media. The rice medium was selected for further scale fermentation, and the EtOAc crude extract was subjected to silica gel, Sephadex LH-20, and semipreparative HPLC to obtain six benzodipyran racemates.

**Figure 2:**
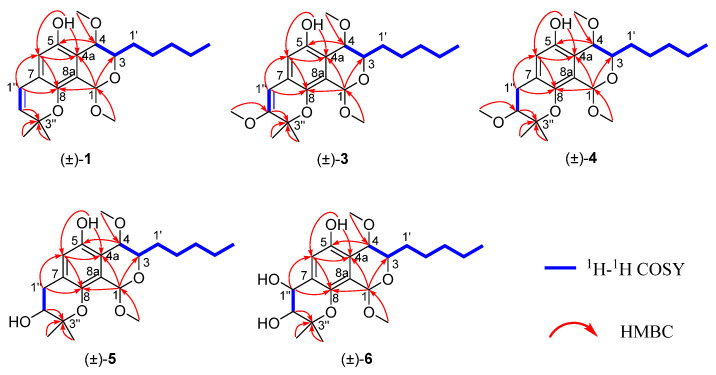
^1^H−^1^H COSY and key HMBC correlations of **1** and **3**–**6**.

Compound **1** was isolated as yellow oil with a molecular formula of C_21_H_30_O_5_ by its HR-ESI-MS *m/z* 361.2003 [M–H]^−^ (calculated for 361.2015), indicating the presence of seven degrees of unsaturation. The UV spectrum showed the absorption maxima at 235, 266, 276, and 341 nm. The IR spectrum showed absorption bands for hydroxyl (3423 cm^−1^), alkane (2937 and 2923 cm^−1^), and phenyl (1560 and 1461 cm^−1^) groups. Comprehensive analyses of the 1D NMR (Table 1) and HSQC spectra of **1** revealed the presence of a penta-substituted benzene ring (*δ*_H_ 6.58 (s, H-6); *δ*_C_ 117.8, 149.1, 113.3, 122.5, 143.4, and 124.1), one cis-disubstituted double bond (*δ*_H_ 6.28 (d, *J* = 10.0, H-1″) and 5.68 (d, *J* = 10.0, H-2″); *δ*_C_ 122.2 and 132.4), an acetal (*δ*_H_ 5.56 (1H, s); *δ*_C_ 95.4), an oxygenated quaternary carbon signal at *δ*_C_ 77.4, two oxygenated methines (*δ*_H_ 4.20 (td, *J* = 6.8, 3.2, H-3) and 4.44 (d, *J* = 3.2, H-4); *δ*_C_ 70.2 and 68.8), two methoxyls (*δ*_H_ 3.54 (s, OCH_3_-1) and 3.18 (s, OCH_3_-4); *δ*_C_ 55.8 and 53.9), four methylenes (*δ*_H_ 1.79 (m, H_2_-1′), 1.66 (m, H_2_-2′), 1.61 (m, H_2_-3′), and 1.51 (m, H_2_-4′); *δ*_C_ 30.4, 25.9, 32.0, and 22.8), and three methyls (*δ*_H_ 0.93 (t, *J* = 6.4, H_3_-5′), 1.41 (s, H_3_-4″), and 1.41 (s, H_3_-5″); *δ*_C_ 14.2, 27.4, and 28.0). The aforementioned spectroscopic data were highly similar to those of eurotiumide G, a natural racemate isolated from the gorgonian-derived fungus *Eurotium* sp. XS-200900E6 [13], suggesting **1** might be the same compound as eurotiumide G. Further interpretation of the ^1^H−^1^H COSY and HMBC spectra of **1** (Figure 2) confirmed the assumption.

**Figure 3:**
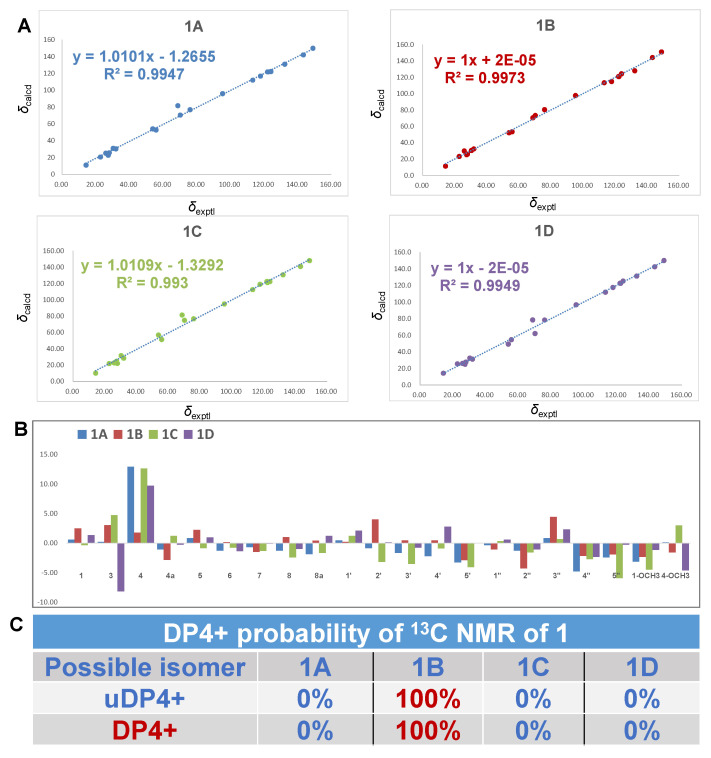
^13^C NMR calculation results of four possible isomers of **1**. (**A**) Linear correlation plots of predicted versus experimental ^13^C NMR chemical shifts; (**B**) relative errors and standard deviations between the predicted ^13^C NMR chemical shifts of four potential structures and recorded ^13^C NMR data; (**C**) the DP4+ probability of ^13^C NMR chemical shifts.

Interestingly, when we attempted to evaluate the relative configuration of **1**, we found that although the NMR data of **1** were consistent with eurotiumide G reported by Wang [13] et al. as a natural racemate from the gorgonian-derived fungus *Eurotium* sp. XS-200900E6, and by Namba [19] et al. as a synthesis through asymmetric total synthesis, their relative configurations were in conflict. Although the structure of original eurotiumide G were claimed to be revised by synthesis, small molecules with different stereo configurations may have highly similar spectra. Meanwhile, according to the key NOE correlations described in the originally reported literature, it seems that the structure of eurotiumide G they proposed is correct. This interesting phenomenon aroused our curiosity and we wondered whether these two molecules happened to have very similar NMR spectra as to be indistinguishable or the original proposed structure of eurotiumide G was incorrect. Therefore, GIAO-based quantum chemical ^13^C NMR calculations at mPW1PW91/6-31+G(d,p) level using chloroform as solution were performed to clarify the relative configuration of **1** (eurotiumide G) unambiguously. There were four possible relative configurations that needed to be taken into account: 1R*,3R*,4R*-**1** (**1A**); 1R*,3S*,4S*-**1** (**1B**); 1S*,3S*,4R*-**1** (**1C**); and 1R*,3S*,4R*-**1** (**1D**). As a result, the calculated carbon chemical shifts of **1B** were in excellent agreement with the experimental data, with coefficient value (R^2^) of 0.9973, while the predicted data for the rest of the configurations did not match the experimental ones as indicated by R^2^ of 0.9947, 0.9930, and 0.9949, respectively. Furthermore, DP4+ probability of 100% in favor of **1B** suggested the relative configuration of the synthesis by Namba et al. was indeed correct, and the relative configuration of **1** was unambiguously concluded as 1R*,3S*,4S* (Figure 3).

**Figure 4:**
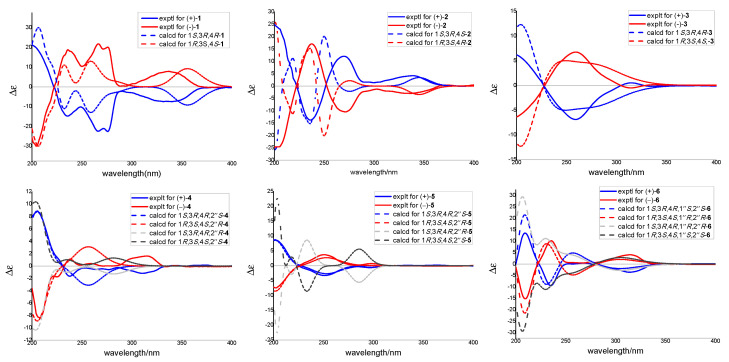
Experimental and calculated ECD spectra of (+)-**1**, (−)-**1**, (+)-**2**, (−)-**2**, (+)-**3**, (−)-**3**, (+)-**4**, (−)-**4**, (+)-**5**, (−)-**5**, (+)-**6**, and (−)-**6**.

**Figure 5:**
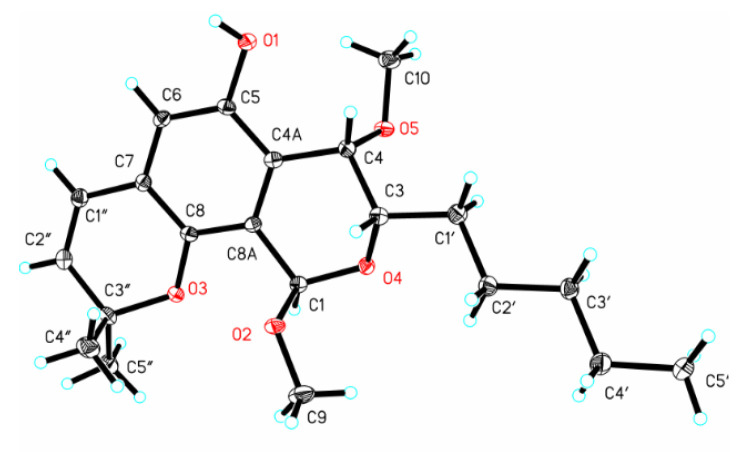
X-ray ORTEP drawing of (−)-**1**.

However, as for the absolute configuration of eurotiumide G enantiomers, although the absolute configurations were previously established using enantioselective synthetic routes [19], the method they used to determine the structure was based on a Mo K*α* radiation of X-ray crystallographic analysis with poor flack parameter of 0.3 (4), which may be unreliable. With the optical rotation value close to zero as well as almost no signal in its ECD spectrum, **1** was assumed to be a racemic mixture and subsequently separated into a pair of enantiomers, (+)-**1** and (−)-**1**, with a ratio of 1:1 via a chiral HPLC column. The absolute configurations of (+)-**1** and (−)-**1** were further determined by quantum chemical ECD calculation (Figure 4) and Cu K*α* radiation of X-ray crystallographic analysis with quality flack parameter of 0.11(16) (Figure 5). Hence, based on the combination of the above evidence, the structure including absolute configurations of **1** was unambiguously established, and the (+) eurotiumide G and (−) eurotiumide G were revised correctly as 1*S*,3*R*,4*R* and 1*R*,3*S*,4*S*, respectively.

Interpretation of the 1D and 2D NMR of **2** indicated that it was eurotiumide F, which was also obtained as a racemate from the gorgonian-derived fungus *Eurotium* sp. XS-200900E6 [13]. Similar to eurotiumide G, the absolute configuration of eurotiumide F was not solved thoroughly. In our experiments, the chiral resolution of **2** was successfully carried out, and the absolute configuration of (+)-**2** and (−)-**2** were determined as 1*S*,3*R*,4*S* and 1*R*,3*S*,4*R* by quantum chemical ECD calculations (Figure 4). In addition, considering the experimental and calculated ECD spectra of compound **2** was complex and did not seem to be perfectly matched in the region between 200 and 220 nm, GIAO-based quantum chemical ^13^C NMR calculations were performed to further confirm correctness of the relative configuration of **2**. As shown in Figure S2, the calculated carbon chemical shifts of 1R*,3S*,4R*-**2** (**2D**) were in excellent agreement with the experimental data with coefficient value (R^2^) of 0.999 and DP4+ probability of 100% (Figure S2), indicating the correct stereochemical assignments of both enantiomers.

Aspergiletal A (**3**) was isolated as yellow oil with a molecular formula of C_22_H_32_O_6_ by its HR-ESI-MS *m*/*z* 391.2120 [M–H]^−^ (calculated for 391.2121), indicating the presence of seven degrees of unsaturation. Comprehensive analyses of the 1D NMR (Table 1) spectra of **3** with those of **1** suggested **3** was highly similar to **1**, and the major differences were the appearance of an additional methoxyl group (δ_H_ 3.15 (s, OCH_3_-2″); δ_C_ 51.3) and an olefinic proton (δ_H_ 5.68 (d, H-2″)) of **1** being absent in **3**. Interpretation of the HMBC spectrum from OCH_3_-2″ to C-2′′ located the methoxyl group at C-2″ (Figure 2). The coupling constant of H-3 and H-4 and key NOE correlations of **3** were similar to **1**, so the relative structure of **3** was determined as 1S*,3R*,4R*. Moreover, **3** was a racemic mixture and successfully separated into a pair of enantiomers (+)-**3** and (−)-**3** by a chiral HPLC column. The absolute configuration of (+)-**3** and (−)-**3** were established as 1*S*,3*R*,4*R* and 1*R*,3S,4*S*, respectively, using the quantum chemical ECD calculation method (Figure 4).

Aspergiletal B (**4**) was isolated as yellow oil with a molecular formula of C_22_H_34_O_6_ by its HR-ESI-MS *m/z* 393.2298 [M–H]^−^, (calculated for 393.2277). Interpretation of the NMR spectroscopic data as well as the molecular formula of **4** indicated that it was a dihydro analogue of **3**. The main difference was the appearance of one methylene (δ_H_ 1.81 (m, H-1″); δ_C_ 22.8] and one oxygenated methine (δ_H_ 4.74 (dt, J = 6.8, 3.2, H-2″); δ_C_ 88.4) in **4** instead of two olefin carbons (δ_H_ 6.57 (s, H-1″), δ_C_ 103.8 and 161.5) in **3**. This conclusion was confirmed by further interpretation of the ^1^H−^1^H COSY and HMBC spectra of **4** (Figure 2). As the structure of **4** contained two isolated stereoclusters, C-1−C-4 in part A and C-1″−C-2″in part B, separated by a pentasubstituted benzene ring, the relative configuration of isolated stereocluster was independently investigated. Based on the coupling constants of the observed protons and key NOESY correlations, the relative configuration of part A was concluded as 1S*,3R*,4R*. Due to the lack of confident NOE correlations, it was insufficient to make conclusions on the relative configurations of two individual stereoclusters using NMR methods alone. The optical values of **4** in MeOH solvent was close to zero, indicating **4** was a racemate. In order to establish the relative and absolute configurations of **4**, it was subsequently separated by a chiral HPLC column, and quantum chemical ECD calculation was carried out. In terms of the whole-compound stereochemistry, four possible absolute configurations needed to be considered: 1*S*,3*R*,4*R*,2″*S*-**4** (**4A**); 1*R*,3*S*,4*S*,2″*R*-**4** (**4B**); 1*S*,3*R*,4*R*,2″*R*-**4** (**4C**); and 1*R*,3*S*,4*S*,2″*S*-**4** (**4D**). As shown in Figure 4, the comparison of predicted ECD curves of 1*S*,3*R*,4*R*,2″*S*-**4** (**4A**) and 1*R*,3*S*,4*S*,2″*R*-**4** (**4B**) with the experimental ones of (+)-**4** and (−)-**4** showed quite good agreement (Figure 4), which allowed the absolute configurations of (+)-**4** and (−)-**4** to be established. Similar to **2**, as the experimental and calculated ECD spectra of compound **4** did not seem to be perfectly matched in the region between 230 and 280 nm, GIAO-based quantum chemical ^13^C NMR calculations were also performed, and the result showed that the stereochemistry of compound **4** that we had derived was correct (Figure S3).

Aspergiletal C (**5**) was isolated as yellow oil with a molecular formula of C_21_H_32_O_6_ by its HR-ESI-MS *m/z* 379.2123 [M−H]^−^, (calculated for 379.2121). The 1D NMR data of **5** was structurally similar to those of **4**, except for the difference of the resonance assigned to C-1″ and absence of the signal corresponding to C-2″ methoxyl (δ_H_ 3.17 (s); δ_C_ 50.2), suggesting the presence of C-2″ hydroxyl in **5**. Similar to **4**, **5** was also a racemate and contained two isolated stereoclusters, and the absolute configurations of (+)-**5** and (−)-**5** were independently assigned as 1*S*,3*R*,4*R*,2″*S*-**5** and 1*R*,3*S*,4*S*,2″*R*-**5** (Figure 4). Certainly, the absolute stereochemical configuration at C-2″ of 5 could be determined by Mosher’s method. However, unfortunately, **5** was only obtained with total amount of 1.0 mg. When **5** was chiral separated by HPLC, only 0.5 mg each of (+)-**5** and (−)-**5** were obtained, making it difficult to perform Mosher’s method. Considering there were two isolated stereoclusters in **5**, the stereochemistry of compound **5** was also confirmed using GIAO-based quantum chemical ^13^C NMR calculations (Figure S4).

**Figure 6:**
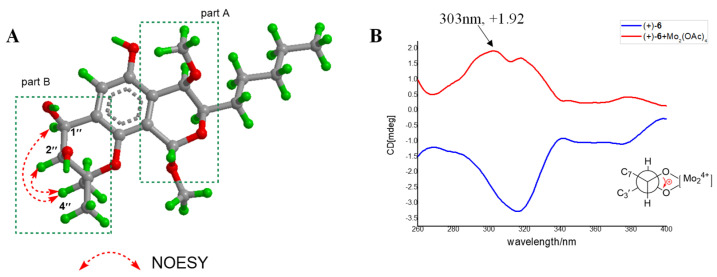
(**A**) Key NOE correlations of **6**; (**B**) CD spectra of (+)-**6** and Mo complex of (+)-**6** recorded in DMSO (the Newman projection represents the preferred conformation of the vic-diol in the chiral Mo complex).

Aspergiletal D (**6**) was isolated as yellow oil with a molecular formula of C_21_H_32_O_7_ by its HR-ESI-MS *m/z* 395.2076 [M−H]^−^, (calculated for 395.2070). The 1D NMR data highly resembled those of **5**, suggesting **6** had the same scaffold as **5**, except for the appearance of the oxygenated methine (δ_H_ 5.31 (d, J = 4.8, H-1″); δ_C_ 74.0) instead of methylene (δ_H_ 3.18 (m, H-1″a) and 3.14 (d, J = 8.4, H-1″b); δ_C_ 31.0). This suggested the presence of C-1″ hydroxyl in **6**. Similarly, **6** was a racemate and contained two isolated stereoclusters, and the relative configuration of part A in **6** was established as 1S*,3R*,4R*. Meanwhile, the key NOE correlations of H-1″/H-2″ and H_3_-4″as well as small coupling constant (*J*_1″,2″_ = 4.8 Hz) for part B in **6** indicated that H-1″ and H-2″ were in cis-diaxial (Figure 6A). The absolute configuration of the 1″,2″-diol unit in part B of **6** could be assigned by measuring the Mo_2_(OAc)_4_ induced circular dichroism spectrum (ICD) of Mo complex of (+)-**6**. The ICD spectrum showed a positive sign of Cotton effect (CE) at 303 nm (Figure 6B), allowing the assignment of C-1″ and C-2″ as 1″*S*,2″*S* based on the empirical rule [20,21]. Finally, the absolute configurations of (+)-**6** and (−)-**6** were independently assigned as 1*S*,3*R*,4*R*,1″*S*,2″*S*-**6** and 1*R*,3*S*, 4*S*,1″*R*,2″*R*-**6** by quantum chemical ECD calculation (Figure 4). Similar to **5**, the stereochemistry of **6** was also confirmed using GIAO-based quantum chemical ^13^C NMR calculations (Figure S5).

**Scheme 1:**
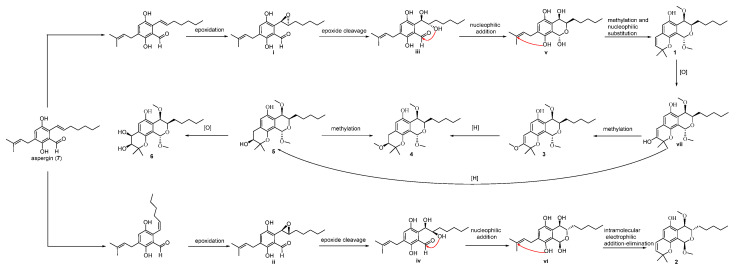
Hypothetical biogenetic pathways for **1**–**6**.

Based on the coisolation of the precursor aspergin (**7**), a plausible biosynthetic pathway for compounds **1**–**6** is proposed in Scheme 1. Epoxidation of the unsaturated C_7_-alkyl chain in **7** was followed by isomerization of the double bond and epoxidation to obtain **i** and **ii**. Subsequently, selective epoxide cleavage of **i** and **ii** were used to obtain the key intermediates **iii** and **iv**, respectively. Then, intermediates **iii** and **iv** were transformed to **v** and **vi**, respectively, under nucleophilic addition. Next, **v** and **vi** were subjected to methylation and intramolecular electrophilic addition–elimination to produce **1** and **2**, respectively. The subsequent sequence of oxidation, methylation, reduction, and oxidation of **1** led to the furnishment of **3**–**6**, respectively.

**Figure 7:**
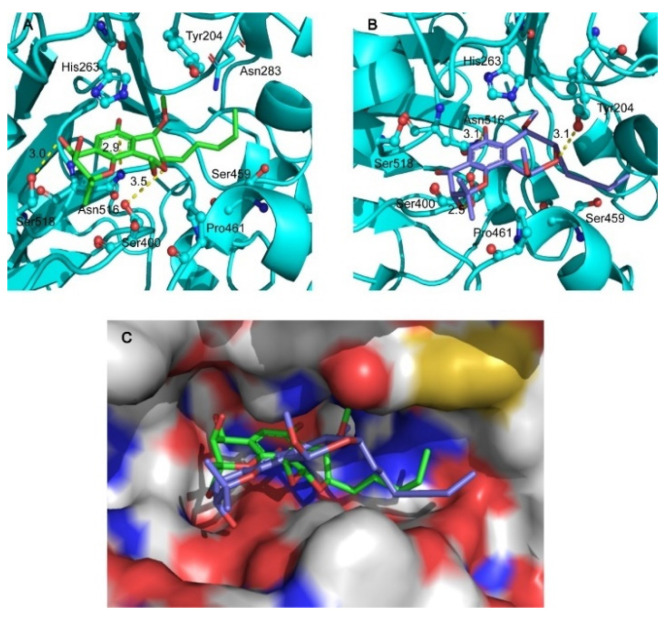
Hypothetical binding mode of (+)-**6** (**A**) or (−)-**6** (**B**) in the complex with TDP1 (PDB: 6DJD). (**C**) Overlay of the binding mode of (+)-**6** or (−)-**6** with TDP1. The TDP1 inhibitor (+)-**6** is represented as a green stick model, (−)-**6** is represented as a purple stick model, and the protein is represented as a cartoon model (**A**,**B**) and as a surface model (**C**).

Considering the chiral properties of compounds will affect their activity, all of the obtained compounds, including racemates and enantiomers, were screened for TDP1 inhibition through a fluorescence assay using a quenched fluorescent single-stranded oligonucleotide (5′-FAM-AGGATCTAAAAGACTT-BHQ-3′) as substrate. Indeed, (+)-**6**, (−)-**6**, and (±)-**6** exhibited different inhibitory activity against the TDP1 enzyme with IC_50_ values of 27.76 ± 1.73, 37.31 ± 3.63, and 6.50 ± 0.73 μM, respectively (Table 2). To further investigate the mechanism of the TDP1 inhibitory activity of (+)-**6** and (−)-**6**, a hypothetical binding mode was built using in silico docking from the X-ray crystal structure of humanized TDP1-inhibitor (PDB: 6DJD) [22] to evaluate the mode of interactions of (+)-**6** and (−)-**6** with TDP1. The inhibitor was docked into the binding site. The hypothetical structural position of the top-ranked (+)-**6** is shown in Figure 7A. The tricyclic core of (+)-**6** binds to the DNA binding groove in the proximity of the catalytically important residue His263, indicating a potential π–π stacking interaction stabilizing the inhibitor TDP1 complex. Three hypothetical hydrogen bonds were observed between the phenol hydroxy group and the amide group of residue Asn516 (2.9 Å), the 1″-hydroxy group and Ser518 residue (3.0 Å), and the pyran O atom and Ser400 residue (3.5 Å), which might contribute to TDP1 inhibition. Similarly, the polycyclic core scaffold of (−)-**6** also lied along the DNA binding groove (Figure 7B), and the H-bonds (3.1 Å) between the phenol hydroxy group and the amide group of residue Asn516 was also observed. In addition, two H-bonds formed between the 2″-hydroxyl group and Ser400 residue (2.5 Å) and the pyran oxygen atom and phenol hydroxy group of Tyr204 residue (3.1 Å). As shown in Figure 7C, the configuration of (+)-**6** was more geometrically suitable for the narrow catalytic pocket than (−)-**6**, leading to closer proximity to the catalytic domain. The gold score values of (+)-**6** and (−)-**6** were 55.62 and 44.90, respectively. Interestingly, although the mode of interactions of (+)-**6** and (−)-**6** with TDP1 were similar, with the racemate (±)-**6** exhibiting stronger inhibitory activity against the TDP1 enzyme with IC_50_ value of 6.50 ± 0.73 μM, which may be a result of synergy between (+)-**6** and (−)-**6**. The mechanism of the TDP1 inhibitory activity of (±)-**6** remains to be validated by biophysical assays in further research.

**Figure 8:**
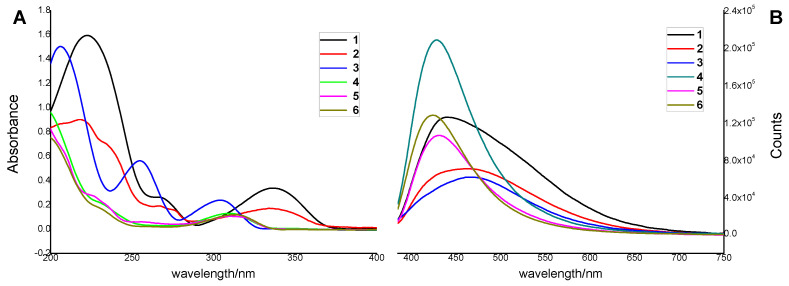
(**A**) Absorption spectra of **1**–**6** in MeOH; (**B**) fluorescence emission spectra of **1**–**6** in MeOH.

**Figure 9:**
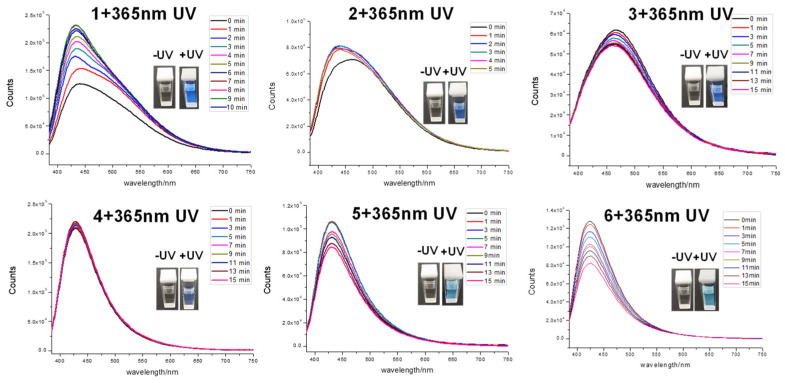
Change in fluorescence emission spectra of **1**–**6** in MeOH upon photoirradiation with 365 nm UV light.

**Figure 10:**
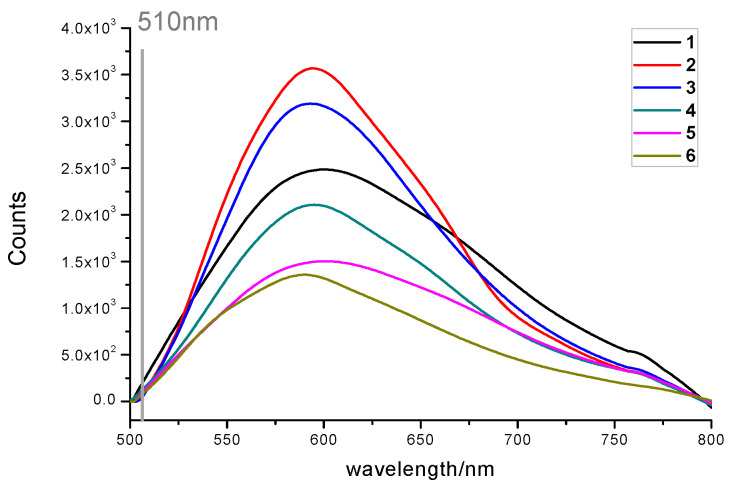
Fluorescence emission spectra of **1**–**6** in MeOH upon photoirradiation with 485 nm UV light with concentration of 100 μM/mL.

In addition, the fluorescent properties of **1**–**6** were also evaluated. Interestingly, all the probes emitted detectable fluorescence (Figure 8). Fluorescence measurements revealed that all the compounds had a large Stokes shift (up to 266 nm, Table 3), suggesting they were capable of the resistance of fluorescence quenching. Surprisingly, the fluorescence of **1** increased substantially with time, indicating a structural change to a more fluorescent, undetermined species upon 365 nm irradiation. Compound **2** responded similarly to **1** but with a smaller magnitude improvement in fluorescence. In contrast, **4** appeared to be inert to photobleaching compared to **3**, **5**, and **6**, all of which showed significant loss of fluorescence with time (Figure 9). Meanwhile, compounds **1**–**6** with the concentration of 100 μM/mL were exposed to 485 nm UV light, and their fluorescence emission spectra were recorded (Figure 10) as a control experiment because the assay used to assess TDP1 inhibition was a fluorescence assay. As the figure shows, **1–6** did not exhibit fluorescence emission spectra at 510 nm when exposed to 485 nm UV light. In addition, during the TDP1 inhibition assay, the tested compound solutions were read by a Flash multimode reader at Ex485/Em510 nm to identify false-positive compounds that had autofluorescence. The above results indicate that the fluorescence of the compounds did not interfere with the inhibition results.

## 3. Materials and Methods

### 3.1. General Experimental Procedures

Details of the instruments and materials used in this work are included in the Supporting Information.

### 3.2. Fungal Strain and Fermentation

The fungal strain EGF 15-0-3 was isolated from a soft coral collected from the South China Sea and identified as *Aspergillus* sp. [23]. A voucher sample of EGF15-0-3 was preserved at the Lab of Marine Natural Medicine, Guangzhou University of Chinese Medicine, P. R. China.

The fungus was cultured on a plate containing PDB medium at 28 °C for 3 days. A single colony was transferred aseptically to an Erlenmeyer flask containing PDB medium and incubated on a rotatory shaker at 165 rpm at 28 °C for 3 days to obtain the seed culture. Subsequently, the seed culture was subcultured in polished glutinous rice medium (autoclaved sterilization with rice and artificial seawater) and incubating at 28 °C for 35 days under static conditions.

### 3.3. Extraction and Isolation

After 35 days of cultivation, the rice medium (10 kg) of EGF 15-0-3 was extracted with ethyl acetate three times (8 h per time) on a rotatory shaker at 165 rpm and concentrated under reduced pressure at 40 °C to yield 50.0 g of the EtOAc residue. The residue was then subjected to silica gel column eluted with a gradient mixture of petroleum ether/ethyl acetate (100:0 to 0:100, *v*/*v*) to obtain 10 major fractions (Fr. A−Fr. J) by TLC analysis. Fr. J (4.0 g) was separated into 7 fractions (Fr. J1−Fr. J7) by Sephadex LH-20 column chromatography (GE Healthcare, China) with MeOH. Fraction J4 (1.2 g) was further separated using preparative HPLC with 80% aqueous MeOH (flow rate of 8.0 mL/min) to yield 13 subfractions (Fr. J41−Fr. J413). Subfraction J45 (*t*_R_ = 15.3 min, 28.0 mg) was purified by repeated semipreparative HPLC with aqueous MeOH (V_MeOH_:V_H__2O_=60:40, flow rate of 3.0 mL/min) to yield **6** (*t*_R_ = 36.2 min, 4.0 mg) and **5** (*t*_R_ = 41.6 min, 1.0 mg); subfraction J49 (*t*_R_ = 20.1 min, 21.0 mg) was purified by repeated semipreparative HPLC using aqueous MeOH as mobile phase (V_MeOH_:V_H__2O_ = 70:30, flow rate of 3.0 mL/min) to yield **3** (*t*_R_ = 30.7 min, 3.0 mg); and subfraction J412 (*t*_R_ = 50.1 min, 107.0 mg) was purified by repeated semipreparative HPLC with aqueous MeOH or MeCN (flow rate of 3.0 mL/min) to yield **1** (*t*_R_ = 25.3 min, 20.0 mg), **4** (*t*_R_ = 28.7 min, 3.0 mg), and **2** (*t*_R_ = 40.2 min, 4.0 mg).

### 3.4. Structural Characterizations of ***1***–***6***

(±)-Eurotiumide G (**1**): yellow oil (MeOH),${\left[\alpha \right]}_{D}^{20}$ 0 (*c* 0.02, MeOH), UV (MeOH) *λ*_max_ (log *ε*) 221 (1.62), 266 (0.28), 336 (0.34) nm; IR (KBr) *ν*_max_ 3423, 2937, 2923, 2900, 1619, 1460, 1260, 1099 cm^−1^; HRESIMS *m/z*: 361.2003 [M–H]^−^ (calculated for C_21_H_29_O_5_ 361.2015); ^1^H MNR (400 MHz) and ^13^C NMR (100 MHz) data in CDCl_3_, see Table 1.

(+)-**1**: ${\left[\alpha \right]}_{D}^{20}$ +12.6 (c 0.30, MeOH), ${\left[\alpha \right]}_{D}^{20}$ +15.0 (c 0.20, CH_2_Cl_2_), CD (MeOH) *λ*_max_ (Δɛ) 235 (−15.8), 267 (−24.0), 332 (−7.9) nm.

(−)-**1**: ${\left[\alpha \right]}_{D}^{20}$ −10.9 (c 0.30, MeOH), ${\left[\alpha \right]}_{D}^{20}$ −15.3 (c 0.20, CH_2_Cl_2_), CD (MeOH) *λ*_max_ (Δɛ) 236 (20.0), 266 (23.2), 332 (8.3) nm.

(±)-Eurotiumide F (**2**): yellow oil (MeOH), ${\left[\alpha \right]}_{D}^{20}$ 0 (c 0.02, MeOH), HRESIMS *m/z*: 361.2023 [M−H]^−^ (calculated for C_21_H_29_O_5_ 361.2015); ^1^H MNR (400 MHz) and ^13^C NMR (100 MHz) data in CDCl_3_, see Table 1.

(+)-**2**: ${\left[\alpha \right]}_{D}^{20}$ +17.2 (c 0.30, MeOH), CD (MeOH) *λ*_max_ (Δɛ) 236 (−13.7), 270 (12.0) nm, 339 (4.2) nm.

(−)-**2**: ${\left[\alpha \right]}_{D}^{20}$ −15.7 (c 0.30, MeOH), CD (MeOH) *λ*_max_ (Δɛ) 237 (17.0), 271 (−10.4) nm, 337 (−3.0) nm.

(±)-Aspergiletal A (**3**): yellow oil (MeOH), ${\left[\alpha \right]}_{D}^{20}$ 0 (c 0.02, MeOH), UV (MeOH) *λ*_max_ (log ε) 206 (1.49), 254 (0.58), 302 (0.24) nm; IR (KBr) *ν*_max_ 3347, 2937, 1699, 1562, 1411, 1347, 1112 cm^−1^; HRESIMS *m/z*: 391.2120 [M−H]^−^(calculated for C_22_H_31_O_6_ 391.2121); ^1^H MNR (400 MHz) and ^13^C NMR (100 MHz) data in CDCl_3_, see Table 1.

(+)-**3**: ${\left[\alpha \right]}_{D}^{20}$ +16.6 (c 0.20, MeOH), CD (MeOH) *λ*_max_ (Δɛ) 257 (−18.5), 309 (1.4) nm.

(−)-**3**: ${\left[\alpha \right]}_{D}^{20}$ −14.0 (c 0.20, MeOH), CD (MeOH) *λ*_max_ (Δɛ) 254 (19.4), 309 (−1.3) nm.

(±)-Aspergiletal B (**4**): yellow oil (MeOH), ${\left[\alpha \right]}_{D}^{20}$ 0 (c 0.02, MeOH), UV (MeOH) *λ*_max_ (log ε) 225 (0.26), 309 (0.13) nm; IR (KBr) *ν*_max_ 3382, 2927, 2856, 1693, 1461, 1380, 1230, 1105, 1041 cm^−1^; HRESIMS *m/z*: 393.2298 [M−H]^−^ (calculated for C_22_H_33_O_6_ 393.2277); ^1^H MNR (400 MHz) and ^13^C NMR (100 MHz) data in CDCl_3_, see Table 1.

(+)-**4**: ${\left[\alpha \right]}_{D}^{20}$ +24.5 (c 0.30, MeOH), CD (MeOH) *λ*_max_ (Δɛ) 206 (8.8), 237 (−1.6), 310 (−1.2) nm.

(−)-**4**: ${\left[\alpha \right]}_{D}^{20}$ −22.6 (c 0.30, MeOH), CD (MeOH) *λ*_max_ (Δɛ) 208 (−8.3), 238 (1.1), 315 (1.6) nm.

(±)-Aspergiletal C (**5**): yellow oil (MeOH), ${\left[\alpha \right]}_{D}^{20}$ 0 (c 0.02, MeOH), UV (MeOH) *λ*_max_ (log ε) 203 (2.53), 249 (3.27), 270 (3.87) nm; IR (KBr) *ν*_max_ 3407, 2933, 2859, 1621, 1461, 1365, 1259, 1016 cm^−1^; HRESIMS *m/z*: 379.2123 [M−H]^−^ (calculated for C_21_H_31_O_6_ 379.2121); ^1^H MNR (400 MHz) and ^13^C NMR (100 MHz) data in CDCl_3_, see Table 1.

(+)-**5**: ${\left[\alpha \right]}_{D}^{20}$ +20.4 (c 0.30, MeOH), CD (MeOH) *λ*_max_ (Δɛ) 202 (8.7), 251 (−3.3), 296 (−0.7) nm.

(−)-**5**: ${\left[\alpha \right]}_{D}^{20}$ −18.2 (c 0.30, MeOH), CD (MeOH) *λ*_max_ (Δɛ) 204 (−7.5), 251 (3.7), 298 (0.7) nm.

(±)-Aspergiletal D (**6**): yellow oil (MeOH), ${\left[\alpha \right]}_{D}^{20}$ 0 (c 0.02, MeOH), UV (MeOH) *λ*_max_ (log ε) 225 (0.22), 311 (0.12) nm; IR (KBr) *ν*_max_ 3384, 2923, 2788, 1685, 1618, 1461, 1382, 1105, 1047 cm^−1^; HRESIMS *m*/*z*: 395.2076 [M–H]^−^ (calculated for C_21_H_31_O_7_ 395.2070); ^1^H MNR (400 MHz) and ^13^C NMR (100 MHz) data in CD_3_OD, see Table 1.

(+)-**6**: ${\left[\alpha \right]}_{D}^{20}$ +27.0 (c 0.30, MeOH), CD (MeOH) *λ*_max_ (Δɛ) 210 (13.5), 235 (−9.5), 313 (−3.6) nm.

(−)-**6**: ${\left[\alpha \right]}_{D}^{20}$ −25.0 (c 0.30, MeOH), CD (MeOH) *λ*_max_ (Δɛ) 209 (−15.2), 235 (10.16), 314 (4.1) nm.

### 3.5. Chiral Separation of ***1***–***6***

Compounds **1***–***6** were subsequently performed on a semipreparative HPLC with a chiral chromatographic column (FLM Chiral ND (2)) (Guangzhou FLM Scientific Instrument Co., Ltd., Guangzhou, China) and successfully separated into the optical pure enantiomers. More detailed information are included in the Supporting Information.

### 3.6. Quantum Chemical Calculations

Firstly, random conformational searches were performed by SYBYL X 2.1.1 program (Tripos) using MMFF94s molecular force field, with an energy cutoff of 10 kcal mol^−1^ to the global minima to obtain the conformers, and the obtained conformers were subsequently subjected to structural optimization using Gaussion 09 software at the B3LYP/6-31+G(d) level. The optimized stable conformers were performed at mPW1PW91/6-311+G(d,p) level with chloroform or methanol as the solvent for quantum chemical NMR calculations, and ECD calculations were performed at the B3LYP/6-31+G* level in the gas phase using Gaussion 09 software package [24] according to previous reports [25]. Boltzmann distributions of the overall NMR and ECD data were estimated and subsequently compared with the experimental ones. The DP4+ analysis was performed according to the published protocol [26]. The ECD spectra were produced by SpecDis 1.70.1 software (University of Wuerzburg, Wuerzburg, Germany) [27].

### 3.7. X-ray Crystallographic Analysis

Crystal (−)-**1** was obtained using the solvent vapor diffusion method. The intensity data of (−)-**1** was recorded on a Rigaku Oxford Diffraction Supernova diffractometer (Agilent SuperNova, America) with Cu Kα radiation (λ = 1.54184 Å). The crystal structure was solved and refined with a similar method reported previously [28]. For the structural refinements, nonhydrogen atoms were refined anisotropically and the hydrogen atom positions were geometrically idealized and allowed to ride on their parent atoms. Crystal data of (−)-**1** in the standard CIF format were deposited with the Cambridge Crystallographic Data Centre with CCDC number 1981300. The crystallographic data of (−)-**1** are shown in Table S1.

Crystal data for (−)-**1**: orthorhombic, C_2__1_H_32_O_5_, M = 362.45, a = 6.0081(2) Å, b=15.4529(5) Å, c = 21.4935(8) Å, *α* = 90, *β* = 90, *γ* = 90, V = 1995.51(12) Å^3^, space group *P*2_1_2_1_2_1_, Z = 4, Dx = 1.206 g/m^3^, µ(Cu Ka) = 0.687 mm^−1^, and F(000) = 784.0. Independent reflections: 3927 (R_int_ = 0.0485, R_sigma_ = 0.0547), The final R1 values were 0.0448, wR2 = 0.0986 [I > 2 σ(I)]. Flack parameter = 0.11(16).

### 3.8. TDP1 Inhibition Assay

The TDP1 inhibition was tested through a fluorescence assay according to a previously reported method [29]. Detailed procedures are included in the Supporting Information.

### 3.9. Molecular Modeling

Molecular Modeling was according to a previously reported method [8]. Details are included in the Supporting Information.

### 3.10. Measurement of Fluorescent Properties

Each probe was prepared at 300 μg/mL in MeOH. The absorption spectra were recorded from 200 to 400 nm using a Chirascan circular dichroism spectrometer (Jasco, Tokyo, Japan). The fluorescence spectra were recorded from 385 to 750 nm with an excitation wavelength of 365 nm using Edinburgh Instruments FLS1000. For UV-irradiated probes, each probe was exposed to 365 nm UV light from a handheld UV lamp for a certain time, and the fluorescence spectra were then recorded using the same method mentioned above.

## 4. Conclusions

Four new benzodipyran racemates representing a rare pyrano[4,3-*h*]chromene scaffold, possibly originating from a cascade sequence of epoxidation, intramolecular electrophilic addition–elimination, and oxidation from the aspergin side chain, were isolated from the soft-coral-derived fungus *Aspergillus* sp. EGF 15-0-3. They were successfully separated by a chiral HPLC, and their absolute stereochemistry were determined according to XRD (Cu K*α*), Snatzke’s method, and ECD calculations. Meanwhile, the structure of eurotiumide G was also revised. The enzyme assay indicated **6** to be a novel TDP1 inhibitor, showing strong TDP1 inhibition ability with IC_50_ value of 6.50 ± 0.73 μM. Preliminary results of molecular docking showed that different configurations of (+)-**6** and (−)-**6** would bring different hydrogen bond interactions and that (+)-**6** was more geometrically suitable for the narrow catalytic pocket than (−)-**6**, leading to closer proximity to the catalytic domain. However, the strong inhibitory activity against the TDP1 enzyme by (±)-**6** indicated that the combination of the enantiomers provided inhibitory synergy. The mechanism of the significant TDP1 inhibitory activity of (±)-**6** remains to be validated by biophysical assays in further research. In addition, the superior fluorescent properties of benzodipyran derivatives could be utilized for elucidating the mode of bioactivities by fluorescence imaging.

## Figures and Tables

**Table 1:** ^1^H NMR data (400 MHz) and ^13^C NMR data (100 MHz) of **1**–**6** (*δ* in ppm, *J* in Hz) ^a^.

No.	1 ^b^		2 ^b^		3 ^b^		4 ^b^		5 ^b^		6 ^c^	
	*δ*_H_, mult, *J*	*δ*_C_, mult	*δ*_H_, mult, *J*	*δ*_C_, mult	*δ*_H_, mult, *J*	*δ*_C_, mult	*δ*_H_, mult, *J*	*δ*_C_, mult	*δ*_H_, mult, *J*	*δ*_C_, mult	*δ*_H_, mult, *J*	*δ*_C_, mult
1	5.56, s	95.4, CH	5.42, s	95.2, CH	5.91, s	95.0, CH	5.51, s	95.3, CH	5.52, s	95.2, CH	5.55, s	96.6, CH
2												
3	4.20, dt (6.8, 3.2)	70.2, CH	4.20, dt (10.0, 3.0)	64.8, CH	4.26, dt (6.8, 3.2)	70.8, CH	4.18, dt (7.2, 3.2)	70.1, CH	4.18, dt (7.0, 3.2)	70.5, CH	4.00, ddd (7.2, 4.4, 1.6)	72.6, CH
4	4.44, d (3.2)	68.8, CH	4.63, d (10.0)	76.0, CH	4.56, d (3.2)	68.9, CH	4.44, d (3.2)	68.7, CH	4.43, d (3.2)	68.9, CH	4.34, d (1.6)	70.9, CH
4a		117.8, C		117.8, C		114.9, C		117.3, C		116.5, C		117.9, C
5		149.1, C		149.4, C		151.3, C		150.5, C		150.2, C		151.4, C
6	6.58, s	113.3, CH	6.51, s	113.4, CH	7.05, s	106.9, CH	6.75, s	112.8, CH	6.76, s	112.9, CH	6.89, s	112.9, CH
7		122.5, C		122.5, C		129.4, C		116.2, C		117.4, C		123.0, C
8		143.4, C		143.0, C		146.3, C		149.3, C		149.6, C		150.9, C
8a		124.1, C		124.2, C		119.9, C		128.8, C		128.8, C		131.3, C
1′	1.79, m	30.4, CH_2_	1.78, m	31.9, CH_2_	1.86, m	30.5, CH_2_	1.65, m	30.8, CH_2_	1.80, m	30.4, CH_2_	1.30, m	31.9, CH_2_
2′	1.66, m	25.9, CH_2_	1.66, m	25.2, CH_2_	1.41, m	26.0, CH_2_	1.61, m	26.0, CH_2_	1.25, m	26.0, CH_2_	1.45, m	26.8, CH_2_
3′	1.61, m	32.0, CH_2_	1.62, m	32.0, CH_2_	1.40, m	32.0, CH_2_	1.39, m	32.0, CH_2_	1.39, m	32.0, CH_2_	1.40, m	33.0, CH_2_
4′	1.51, m	22.8, CH_2_	1.51, m	22.8, CH_2_	1.40, m	22.8, CH_2_	1.51, m	30.4, CH_2_	1.33, m	22.8, CH_2_	1.42, m	23.7, CH_2_
5′	0.93, t (6.4)	14.2, CH_3_	0.92, t (7.2)	14.2, CH_3_	0.94, t (6.8)	14.3, CH_3_	0.93, t (6.6)	14.2, CH_3_	0.93, t (6.4)	14.2, CH_3_	0.95, t (6.8)	14.4, CH_3_
1″	6.28, d (10.0)	122.2, CH	6.28, d (9.6)	122.1, CH	6.57, s	103.8, CH	1.81, m	22.8, CH_2_	3.18, m ^a^3.14, d (8.4)	31.0, CH_2_	5.31, d (4.8)	74.0, CH
2″	5.68, d (10.0)	132.4, CH	5.68, d (9.6)	132.2, CH		161.5, C	4.74, dt (6.8, 3.2)	88.4, CH	4.69, t (8.4)	89.7, CH	4.20, t (4.8)	98.3, CH
3″		77.4, C		77.4, C		73.6, C		76.1, C		72.1, C		72.0, C
4″	1.41, s	27.4, CH_3_	1.41, s	27.6, CH_3_	1.62, s	25.9, CH_3_	1.24, s	22.2, CH_3_	1.33, s	24.3, CH_3_	1.27, s	25.4, CH_3_
5″	1.41, s	28.0, CH_3_	1.40, s	28.0, CH_3_	1.61, s	25.1, CH_3_	1.13, s	19.6, CH_3_	1.19, s	26.1, CH_3_	1.25, s	25.8, CH_3_
1-OCH_3_	3.54, s	55.8, CH_3_	3.53, s	56.1, CH_3_	3.63, s	55.9, CH_3_	3.53, s	55.8, CH_3_	3.53, s	55.6, CH_3_	3.45, s	58.4, CH_3_
4-OCH_3_	3.18, s	53.9, CH_3_	3.18, s	51.4, CH_3_	3.20, s	53.9, CH_3_	3.26, s	53.8, CH_3_	3.19, s	53.9, CH_3_	3.35, s	49.6, CH_3_
2″-OCH_3_					3.15, s	51.3, CH_3_	3.17, s	50.2, CH_3_				

^a^ Overlapped signals are reported without designating multiplicity, ^b^ in CDCl_3_, ^c^ in CD_3_OD.

**Table 2:** TDP1 enzyme inhibition of the isolated compounds **1***–***6** (IC_50_ ± SD in μM) ^a^.

Compound	TDP1 Inhibition (μM)	Compound	TDP1 Inhibition (μM)
(±)-**1**	>100	(±)-**4**	>100
(+)-**1**	>100	(+)-**4**	>100
(−)-**1**	>100	(−)-**4**	>100
(±)-**2**	57.81 ± 2.20	(±)-**5**	>100
(+)-**2**	>100	(+)-**5**	>100
(−)-**2**	>100	(−)-**5**	>100
(±)-**3**	>100	(±)-**6**	6.50 ± 0.73
(+)-**3**	>100	(+)-**6**	27.76 ± 1.73
(−)-**3**	>100	(−)-**6**	37.31 ± 3.63

^a^ IC_50_ value, defined as the concentration of the compound to inhibit 50% of TDP1 activity. Every experiment was independently repeated at least three times.

**Table 3:** Fluorescent properties of **1**–**6** ^a^.

Probes	*λ*_abs_	*λ*_em_^b^	Stokes Shift	ε [M^–1^cm^–1^]	*φ*_F_
**1**	221	445	224	125388.61	0.0392
**2**	223	464	241	70590.11	0.0225
**3**	206	472	266	61279.01	0.0048
**4**	225	428	203	208307.41	0.0649
**5**	203	432	229	106186.60	0.0482
**6**	225	424	199	127827.28	0.0416

^a^ All test samples were prepared at the concentration of 300 μg/mL in MeOH. ^b^ Determined at *λ*exc = 365 nm.

## Supplementary Materials

The following supporting information can be downloaded at https://www.mdpi.com/article/10.3390/md20030211/s1, Figure S1. UPLC-MS/MS analyses of the EtOAc extract of *Aspergillus* sp. EGF15-0-3 in different media; Figures S2–S5: ^13^C NMR calculation results of possible isomers of **2**, **4**, **5**, and **6**; Figures S6–S11: Chiral separations of **1**–**6**; Table S1: Crystal data and structure refinement for (−)-**1**; Tables S2–S6: Experimental chemical shifts and calculated unscaled shifts for PD4+ probability analysis for **1**, **2**, **4**, **5**, and **6**; Tables S7–S15: Details of ECD calculations of **1**–**6**; Figures S12–S17: Key molecular orbitals **1**–**6**; Figures S18–S67: the UV, IR, HRESIMS, 1D NMR, and 2D NMR spectra of compounds **1**–**6**.

## References

[1] Dexheimer T.S., Antony S., Marchand C., Pommier Y. (2008). Tyrosyl-DNA Phosphodiesterase as a target for anticancertherapy. Anti-Cancer Agents Med. Chem..

[2] Comeaux E.Q., Waardenburg R.C.A.M.V. (2014). Tyrosyl-DNA phosphodiesterase I resolves both naturally and chemically induced DNA adducts and its potential as a therapeutic target. Drug Metab. Rev..

[3] Interthal H., Pouliott J.J., Champoux J.J. (2001). The tyrosyl-DNA phosphodiesterase Tdp1 is a member of the phospholipase D superfamily. Proc. Natl. Acad. Sci. USA.

[4] Sun Y., Saha S., Wang W., Saha L.K., Huang S.N., Pommier Y. (2020). Excision repair of topoisomerase DNA-protein crosslinks (TOPDPC). DNA Repair..

[5] Nivens M.C., Felder T., Galloway A.H., Pena M.M., Pouliot J.J., Spencer H.T. (2004). Engineered resistance to camptothecin and antifolates by retroviral coexpression of tyrosyl DNA phosphodiesterase-I and thymidylate synthase. Cancer Chemother. Pharmacol..

[6] Barthelmes H.U., Habermeyer M., Christensen M.O., Mielke C., Interthal H., Pouliot J.J., Boege F., Marko D. (2004). TDP1 overexpression in human cells counteracts DNA damage mediated by topoisomerases I and II. J. Biol. Chem..

[7] Zakharenko A., Luzina O., Koval O., Nilov D.I., Gushchina N., Dyrkheeva V., Svedas N., Salakhutdinov O.L. (2016). Tyrosyl-DNA phosphodiesterase 1 inhibitors: Usnic acid enamines enhance the cytotoxic effect of camptothecin. J. Nat. Prod..

[8] Zhang X.R., Wang H.W., Tang W.L., Zhang Y., Yang H., Hu D.X., Ravji A., Marchand C., Kiselev E., Ofori-Atta K. (2018). Discovery, Synthesis, and evaluation of oxynitidine derivatives as dual Inhibitors of DNA topoisomerase IB (TOP1) and tyrosyl-DNA phosphodiesterase 1 (TDP1), and potential antitumor agents. J. Med. Chem..

[9] Kovaleva K., Oleshko O., Mamontova E., Yarovaya O., Zakharova O., Zakharenko A., Kononova A., Dyrkheeva N., Cheresiz S., Pokrovsky A. (2019). Dehydroabietylamine ureas and thioureas as tyrosyl-DNA phosphodiesterase 1 inhibitors that enhance the antitumor effect of temozolomide on glioblastoma cells. J. Nat. Prod..

[10] Laev S.S., Salakhutdinov N.F., Lavrik O.I. (2016). Tyrosyl-DNA phosphodiesterase inhibitors: Progress and potential. Bioorgan. Med. Chem..

[11] Bantick J.R., Cairns H., Chambers A., Hazard R., King J., Lee T.B. (1976). Benzodipyran derivatives with antiallergic activity. J. Med. Chem..

[12] Chen C.Y., Liu N.Y., Lin H.C., Lee C.Y., Hung C.C., Chang C.S. (2016). Synthesis and bioevaluation of novel benzodipyranone derivatives as P-glycoprotein inhibitors for multidrug resistance reversal agents. Eur. J. Med. Chem..

[13] Chen M., Shao C.L., Wang K.L., Xu Y., She Z.G., Wang C.Y. (2014). Dihydroisocoumarin derivatives with antifouling activities from a gorgonian-derived *Eurotium* sp. fungus. Tetrahedron.

[14] Xiao L.G., Zhang Y., Zhang H.L., Li D., Gu Q., Tang G.H., Yu Q., An L.K. (2020). Spiroconyone A, a new phytosterol with a spiro [5,6] ring system from *Conyza japonica*. Org. Biomol. Chem..

[15] Zhang H.L., Zhang Y., Yan X.L., Xiao L.G., Hu D.X., Yu Q., An L.K. (2020). Secondary metabolites from *Isodon ternifolius* (D. Don) Kudo and their anticancer activity as DNA topoisomerase IB and Tyrosyl-DNA phosphodiesterase 1 inhibitors. Bioorg. Med. Chem..

[16] Wei X., Su J.C., Hu J.S., He X.X., Lin S.J., Zhang D.M., Ye W.C., Chen M.F., Lin H.W., Zhang C.X. (2022). Probing indole diketopiperazine-based hybrids as environmental-induced products from *Aspergillus* sp. EGF 15-0-3. Org. Lett..

[17] Cheng Y.J., Chen N.N., Li J., Su J.C., Yang J.Y., Zhang C.X., Lin H.W., Zhou Y.J. (2021). Antimicrobial chlorinated carbazole alkaloids from the sponge-associated actinomycete *Streptomyces diacarni* LHW51701. Chin. J. Chem..

[18] Liu B.X., Wei X., Xiao X.J., Zhang Q., Zhang C.X. (2021). Research on benzaldehydes from the soft coral-associated symbiotic fungus *Aspergillus* sp. EGF15-0-3. J. Trop. Oceanogr..

[19] Nakayama A., Sato H., Nagano S.J., Karanjit S., Imagawa H., Namba K. (2019). Asymmetric total syntheses and structure elucidations of (+)-eurotiumide F and (+)-eurotiumide G. Chem. Pharm. Bull..

[20] Snatzke G., Wagner U., Wolff H.P. (1981). Circulardichroism—LXXV1: Cottonogenic derivatives of chiral bidentate ligands with the complex [Mo_2_ (O_2_CCH_3_)_4_]. Tetrahedron.

[21] Górecki M., Jabłońska E., Kruszewska A., Suszczyńska A., Urbańczyk-Lipkowska Z., Gerards M., Morzycki J.W., Szczepek W.J., Frelek J. (2007). Practical method for the absolute configuration assignment of *tert*/*tert* 1,2-diols using their complexes with Mo_2_(OAc)_4_. J. Org. Chem..

[22] Lountos G.T., Zhao X.Z., Evgeny K., Tropea J.E., Needle D., Pommier Y., Burke T.R., Waugh D.S. (2019). Identification of a ligand binding hot spot and structural motifs replicating aspects of tyrosyl-DNAphosphodiesterase I (TDP1) phosphoryl recognitionby crystallographic fragment cocktail screening. Nucleic Acids Res..

[23] Wei X., Feng C., Wang S.Y., Zhang D.M., Li X.H., Zhang C.X. (2020). New indole diketopiperazine alkaloids from soft coral-associated epiphytic fungus *Aspergillus* sp. EGF 15-0-3. Chem. Biodive..

[24] Frisch M.J., Trucks G.W., Schlegel H.B., Scuseria G.E., Robb M.A., Cheeseman J.R., Scalmani G., Barone V., Mennucci B., Petersson G.A. (2009). Gaussian 09, Revision A.02.

[25] Song J.G., Su J.C., Song Q.Y., Huang R.L., Tang W., Hu L.J., Huang X.J., Jiang R.W., Li Y.L., Ye W.C. (2019). Cleistocaltones A and B, Antiviral phloroglucinol−terpenoid adducts from *Cleistocalyx operculatus*. Org. Lett..

[26] Grimblat N., Zanardi M.M., Sarotti A.M. (2015). Beyond DP4: An improved probability for the stereochemical assignment of isomeric compounds using quantum chemical calculations of NMR shifts. J. Org. Chem..

[27] Bruhn T., Schaumloffel A., Hemberger Y., Pescitelli G. (2017). SpecDis Version 1.70.

[28] Su J.C., Wang S., Cheng W., Huang X.J., Li M.M., Jiang R.W., Li Y.L., Wang L., Ye W.C., Wang. Y. (2018). Phloroglucinol derivatives with unusual skeletons from *Cleistocalyx operculatus* and their in vitro antiviral activity. J. Org. Chem..

[29] Hu D.X., Tang W.L., Zhang Y., Yang H., Wang W., Agama K., Pommier Y., An L.K. (2021). Synthesis of methoxy-, methylenedioxy-, hydroxy-, and haloSubstituted benzophenanthridinone derivatives as DAN topoisomerase IB (TOP1) and tyrosyl-DNA phosphodiesterase 1 (TDP1) inhibitors and their biological activity for drug-resistant cancer. J. Med. Chem..

