# Supplementary material for: Pyranodipyran Derivatives with Tyrosyl DNA Phosphodiesterase 1 Inhibitory Activities and Fluorescent Properties from Aspergillus sp. EGF 15-0-3
Source: Mar Drugs. 2022 Mar 17;20(3):211. doi: 10.3390/md20030211 (PMC8954640; doi:10.3390/md20030211)
Supplement: Supplementary file 1 [file marinedrugs-20-00211-s001.zip › marinedrugs-1624199-supplementary.pdf]

Supporting Information

Pyranodipyran Derivatives with Tyrosyl DNA Phosphodiesterase 1 Inhibitory Activities and Fluorescent Properties from *Aspergillus* sp. EGF 15-0-3

Xia Wei[†,#], Fang-Ting Wang[‡,#], Mei-Xia Si-Tu[†], Hao Fan[†], Jin-Shan Hu[†], Hao Yang[‡], Shan-Yue Guan[§], Lin-Kun An[*,‡], Cui-Xian Zhang[*,†]

[†] School of Pharmaceutical Sciences, Guangzhou University of Chinese Medicine, Guangzhou 510006, People's Republic of China

[‡] School of Pharmaceutical Sciences, Sun Yat-Sen University, Guangzhou 510006, People's Republic of China

[§]Instrumental analysis & Research center, Sun Yat-Sen University, Guangzhou 510275, People's Republic of China

[#] They are equal to the manuscript.

Figure S1. UPLC-MS/MS analyses of the EtOAc extract of *Aspergillus sp.* EGF15-0-3 in different media....S4-S5

Figure S2. ^{13}C NMR calculation results of four possible isomers of **2**....S5

Figure S3. ^{13}C NMR calculation results of two possible isomers of **4**....S6

Figure S4. ^{13}C NMR calculation results of two possible isomers of **5**....S7

Figure S5. ^{13}C NMR calculation results of four possible isomers of **6**....S8

Experimental Section

1. General Experimental Procedures....S9

2. Chiral separations of **1–6**....S9-S11

3. X-ray crystallographic analysis of (–)-**1**....S11

4. Quantum chemical ^{13}C NMR calculations of **1**....S12-S15

5. Quantum chemical ECD calculations of **1–6**....S15-S29

6. TDP1 inhibition assay....S29-S30

7. Molecular Modelling....S30

Spectral Information

Figure S18. UV spectrum of **1** in MeOH....S31

Figure S19. IR spectrum of **1** (KBr disc) S31

Figure S20. HRESIMS spectrum of **1**....S31

Figure S21-S27. 1D and 2D NMR spectra of **1** in $CDCl_3$....S32-S35

Figure S28. UV spectrum of **3** in MeOH....S35

Figure S29. IR spectrum of **3** (KBr disc) S36

Figure S30. HRESIMS spectrum of **3**....S36

Figure S31-S37. 1D and 2D NMR spectra of **3** in $CDCl_3$....S36-S39

Figure S38. UV spectrum of **4** in MeOH....S40

Figure S39. IR spectrum of **4** (KBr disc) S40

Figure S40. HRESIMS spectrum of **4**...S40

Figure S41-S47. 1D and 2D NMR spectra of **4** in $CDCl_3$..S41-S44

Figure S48. UV spectrum of **5** in MeOH...S44

Figure S49. IR spectrum of **5** (KBr disc) S45

Figure S50. HRESIMS spectrum of **5**...S45

Figure S51-S57.1D and 2D NMR spectra of **5** in $CDCl_3$...S45-S48

Figure S58. UV spectrum of **6** in MeOH...S49

Figure S59. IR spectrum of **6** (KBr disc) S49

Figure S60. HRESIMS spectrum of **6**...S49

Figure S61-S67.1D and 2D NMR spectra of **6** in CD_3OD..S50-S53

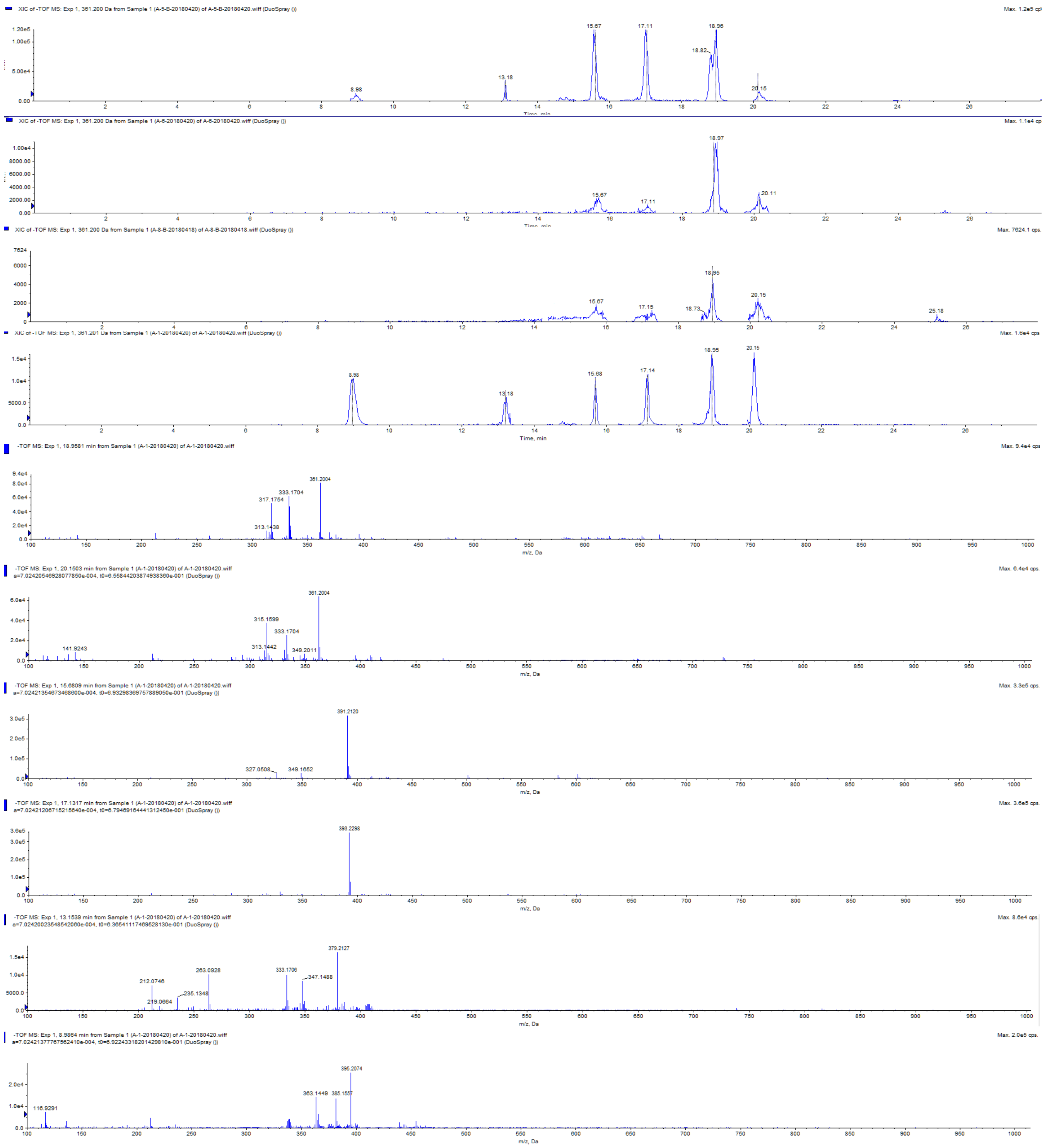

Figure S1. UPLC-MS/MS analyses of the EtOAc extract of *Aspergillus sp.* EGF15-0-3 in different media (A: Extracted ion chromatogram of the EtOAc extract in PDB medium; B: Extracted ion chromatogram of the EtOAc extract in Z-1 medium; C: Extracted ion chromatogram of the EtOAc extract in Z-3 medium, D: Extracted ion chromatogram of the EtOAc extract in rice medium, Peak I: retention time: 18.958 min, *m/z* 361.2004 $[M-H]^-$, Peak II: retention time: 20.153 min, *m/z* 361.2004 $[M-H]^-$, Peak III: retention time: 15.680 min, *m/z* 391.2120 $[M-H]^-$, Peak IV: retention time: 17.131 min, *m/z* 393.2298 $[M-H]^-$, Peak V:

retention time: 13.153 min, *m/z* 379.2127 [M–H]⁻, Peak VI: retention time: 8.986 min, *m/z* 395.2074 [M–H]⁻).

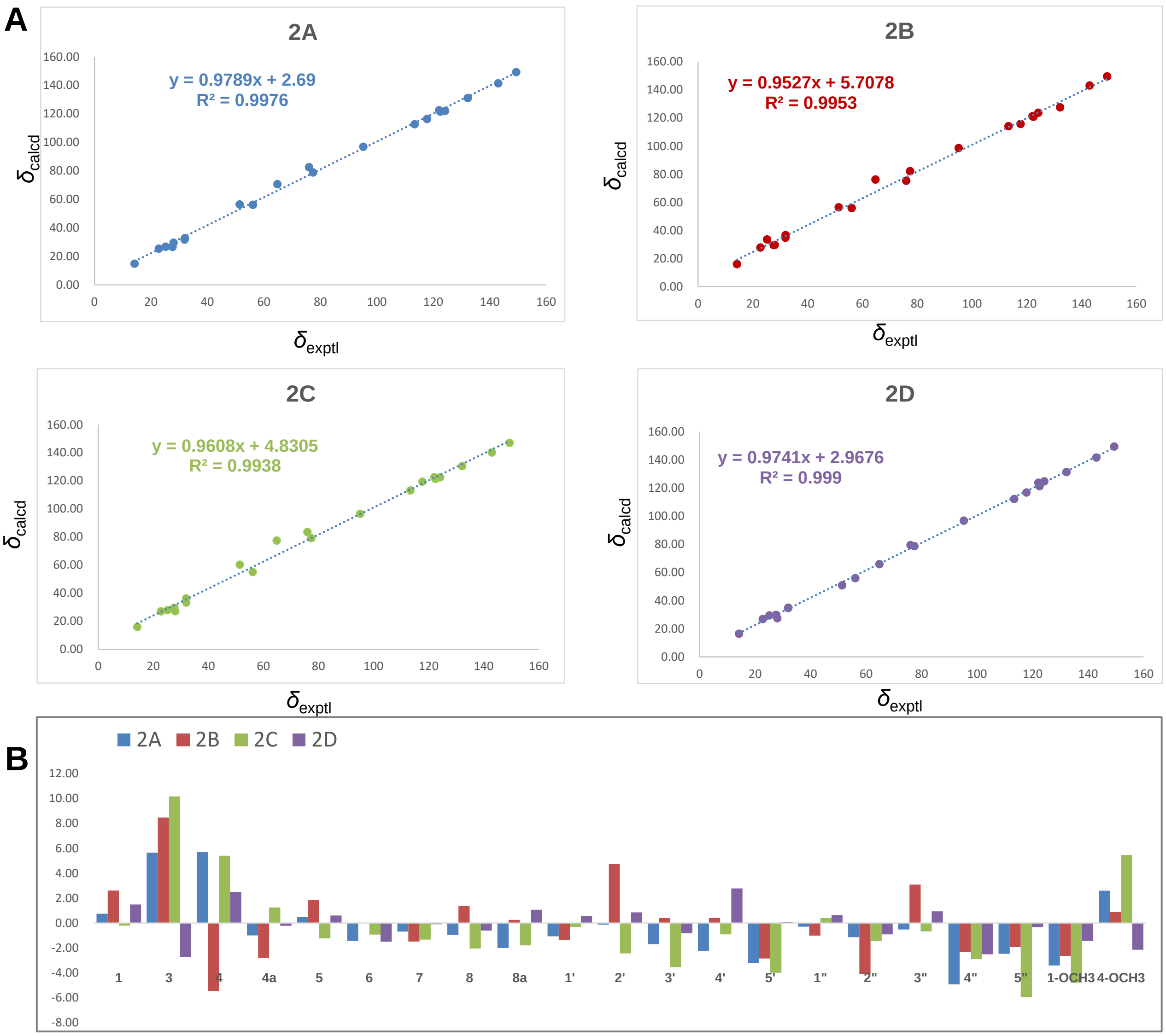

C

DP4+ probability of ^{13}C NMR of 2				
Possible isomer	2A	2B	2C	2D
uDP4+	0%	0%	0%	100%
DP4+	0%	0%	0%	100%

Figure S2. ^{13}C NMR calculation results of four possible isomers of **2**. (**A**) Linear correlation plots of predicted versus experimental ^{13}C NMR chemical shifts. (**B**) Relative errors and standard derivations between the predicted ^{13}C NMR chemical shifts of four potential structures and recorded ^{13}C NMR data. (**C**) The DP4+ probability of ^{13}C NMR chemical shifts. 1*R**,3*R**,4*R**-**2** (**2A**), isomer 2 is 1*R**,3*S**,4*S**-**2** (**2B**), isomer 3 is 1*S**,3*S**,4*R**-**2** (**2C**), and isomer 4 is 1*R**,3*S**,4*R**-**2** (**2D**).

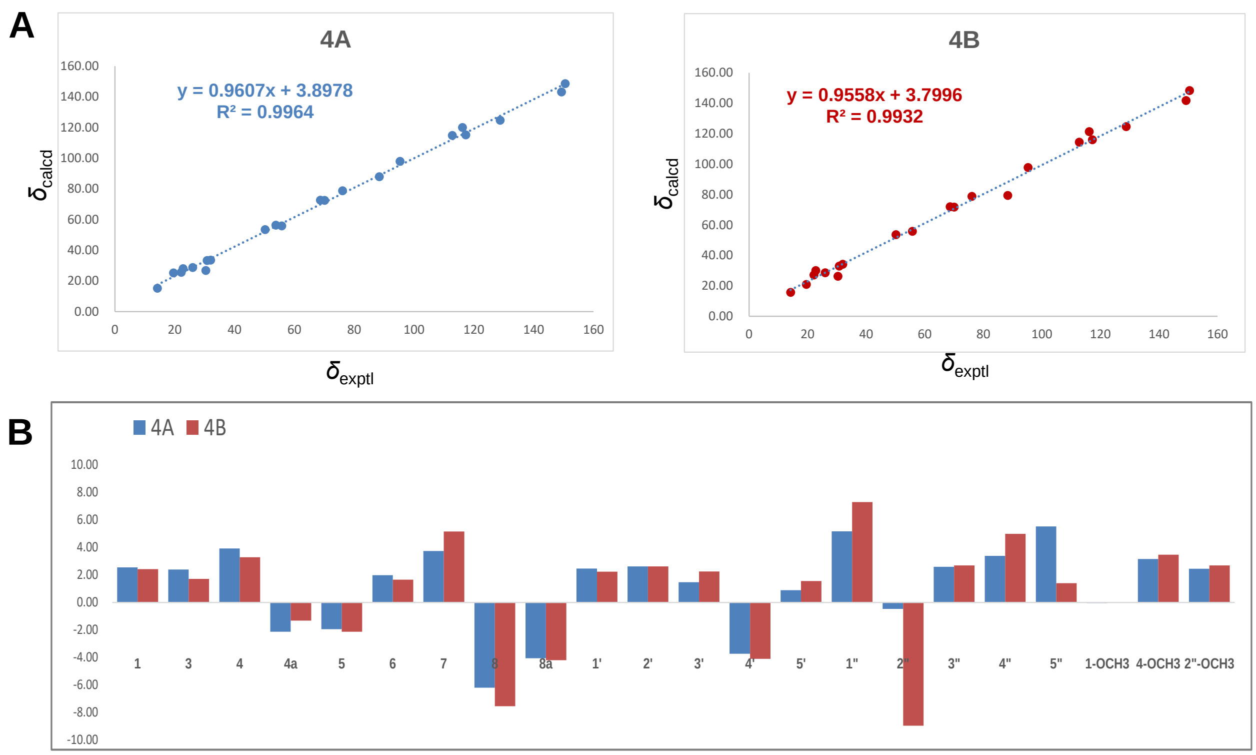

C

DP4+ probability of ^{13}C NMR of 4

Possible isomer	4A	4B
uDP4+	100%	0%
DP4+	100%	0%

Figure S3. ^{13}C NMR calculation results of two possible isomers of **4** (**A**) Linear correlation plots of predicted versus experimental ^{13}C NMR chemical shifts. (**B**) Relative errors and standard derivations between the predicted ^{13}C NMR chemical shifts of four potential structures and recorded ^{13}C NMR data. (**C**) The DP4+ probability of ^{13}C NMR chemical shifts. 1*R**,3*S**,4*S**,2′′*R**-**4** (**4A**), and 1*R**,3*S**,4*S**,2′′*S**-**4** (**4B**).

A

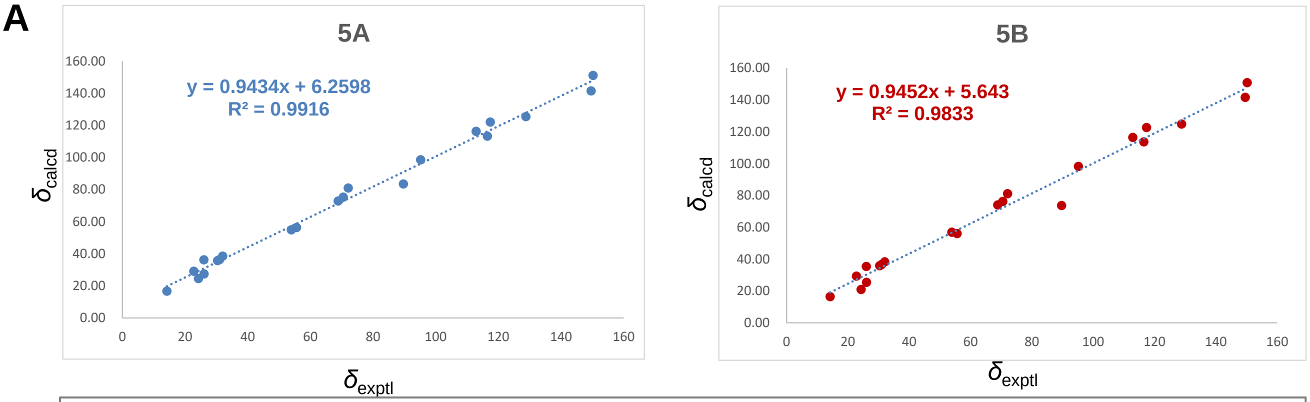

B

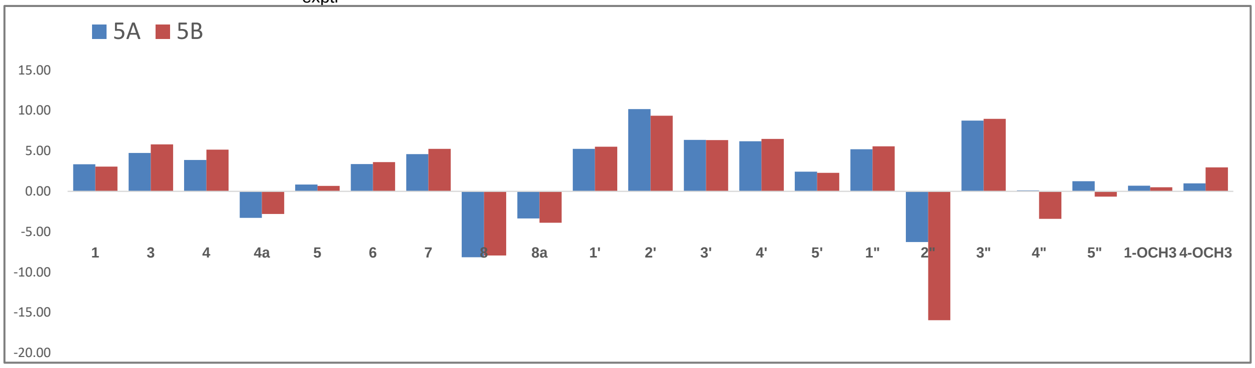

C

DP4+ probability of ^{13}C NMR of 5		
Possible isomer	5A	5B
uDP4+	100%	0%
DP4+	100%	0%

Figure S4. ^{13}C NMR calculation results of two possible isomers of **5** (**A**) Linear correlation plots of predicted versus experimental ^{13}C NMR chemical shifts. (**B**) Relative errors and standard derivations between the predicted ^{13}C NMR chemical shifts of four potential structures and recorded ^{13}C NMR data. (**C**) The DP4+ probability of ^{13}C NMR chemical shifts. 1*R**,3*S**,4*S**,2′′*R**-**5** (**5A**), and 1*R**,3*S**,4*S**, 2′′*S**-**5** (**5B**).

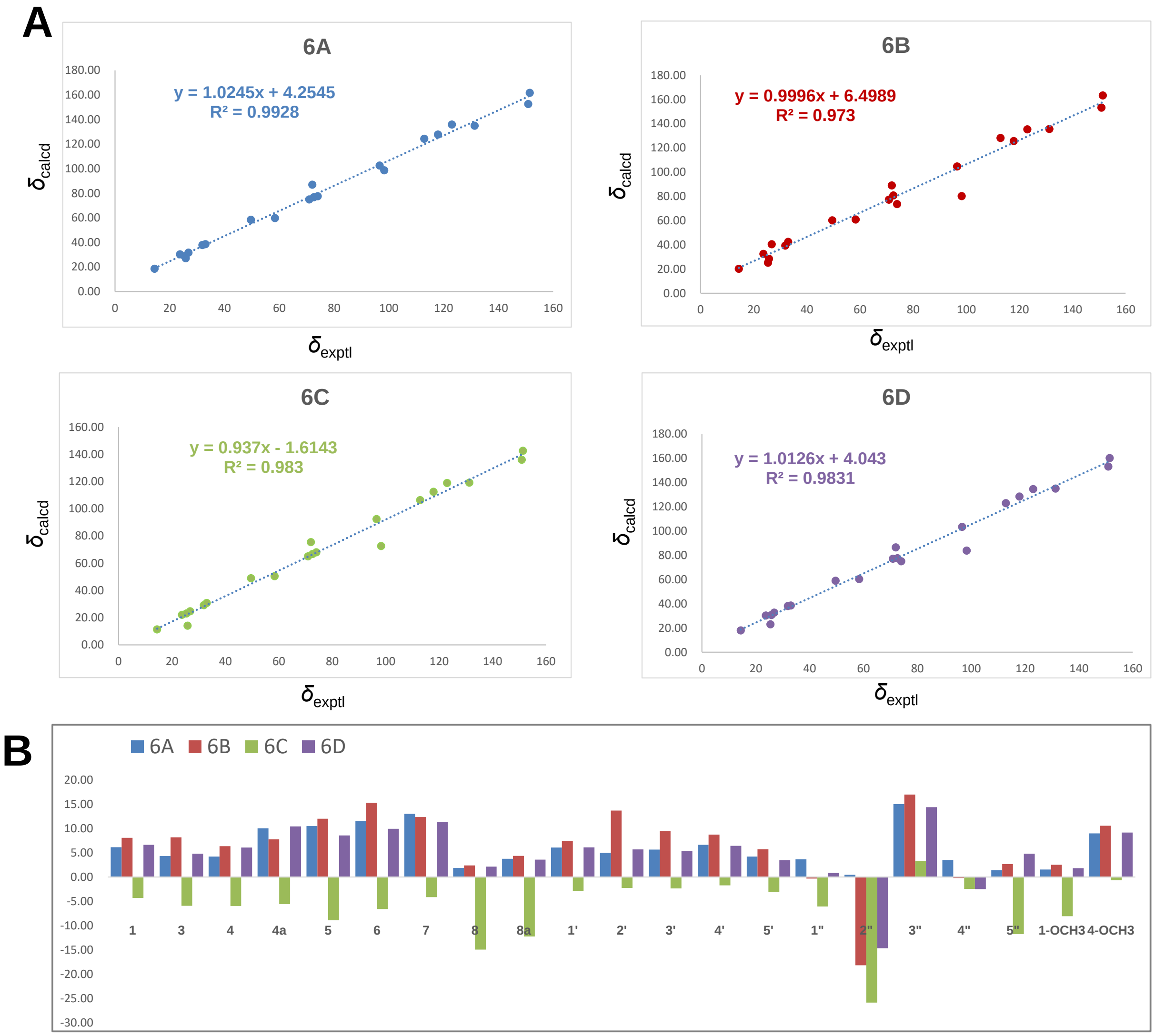

C

DP4+ probability of ^{13}C NMR of 6				
Possible isomer	6A	6B	6C	6D
uDP4+	100%	0%	0%	0%
DP4+	100%	0%	0%	0%

Figure S5. ^{13}C NMR calculation results of four possible isomers of **6**. (**A**) Linear correlation plots of predicted versus experimental ^{13}C NMR chemical shifts. (**B**) Relative errors and standard derivations between the predicted ^{13}C NMR chemical shifts of four potential structures and recorded ^{13}C NMR data. (**C**) The DP4+ probability of ^{13}C NMR chemical shifts. 1*R**,3*S**,4*S**,1′′*R**,2′′*R**-**6** (**6A**), 1*R**,3*S**,4*S**,1′′*S**,2′′*S**-**6** (**6B**), 1*R**,3*S**,4*S**,1′′*R**,2′′*S**-**6** (**6C**), and 1*R**,3*S**,4*S**,1′′*S**,2′′*R**-**6** (**6D**).

Experimental Section

1. General Experimental Procedures.

Optical rotations were performed on a MCP 200 digital polarimeter (Anton Paar). UV and ECD data were recorded with a Chirascan circular dichroism spectrometer (Applied Photophysics). Fluorescence spectra were recorded with Edinburgh instruments FLS1000. IR spectra were acquired with a Nicolet6700-Continum Fourier transform infrared spectrometer and microscope spectrometer (Thermo Scientific). NMR spectra were recorded on a Bruker AVANCE III HD 400 MHz DIGITAL NMR spectrometer (Bruker Optics) with tetramethylsilane as the internal standard. HR-ESI-MS were obtained on a Triple TOFTM 5600+ systems (AB SCIEX). The crystallographic data were recorded on a Rigaku Oxford Diffraction Supernova diffractometer with Cu Kα radiation. HPLC was operated by using HPLC (quiksep, H & E). Column chromatography (CC) was performed on 200-300 mesh silica gel (Qingdao Marine Chemical Factory, China) and Sephadex LH-20 (Amersham Pharmacia). Fractions were monitored by TLC with silica gel GF_{254} on glass plates (Qingdao Marine Chemical Factory, China), and spots were visualized under ultraviolet transmission reflectometer (Shanghai Jingke Industrial).

3. Chiral Separation of 1—6

Except for **5**, the mobile phases of all the remaining compounds were isopropanol−*n*-hexane (10:90, v/v) and the flow rate was 3.0 mL/min. And followed by obtained (+)-**1** (4.0 mg, t_R= 9.102 min) and (–)-**1** (4.0 mg, t_R= 13.768 min) (Figure S6), (+)-**2** (2.0 mg, t_R= 6.863 min) and (–)-**2** (2.0 mg, t_R= 6.438 min) (Figure S7), (+)-**3** (1.0 mg, t_R=15.155 min) and (–)-**3** (1.0 mg, t_R= 10.788 min) (Figure S8), (+)-**4** (0.8 mg, t_R= 11.363 min) and (–)-**4** (0.8 mg, t_R= 14.613 min) (Figure S9), (+)-**6** (1.0 mg, t_R= 7.782 min) and (–)-**6** (1.0 mg, t_R= 6.882 min) (Figure S11), respectively. While **5** was used with isopropanol−*n*-hexane (8:92, v/v) and to yield (+)-**5** (0.5 mg, t_R= 13.132 min) and (–)-**5** (0.5 mg, t_R= 14.948 min) (Figure S10).

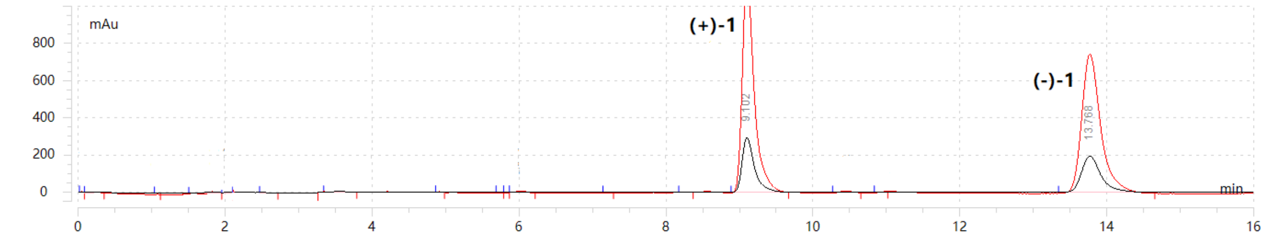

Figures S6. The chiral HPLC analysis of **1**.

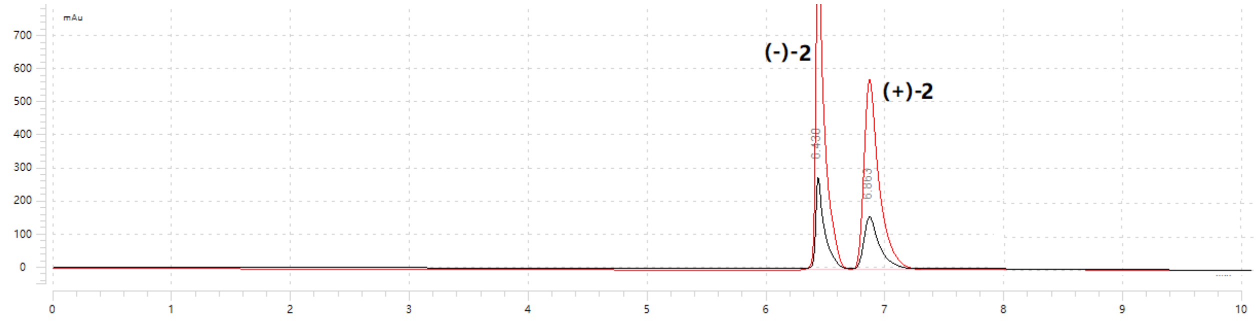

Figures S7. The chiral HPLC analysis of **2**.

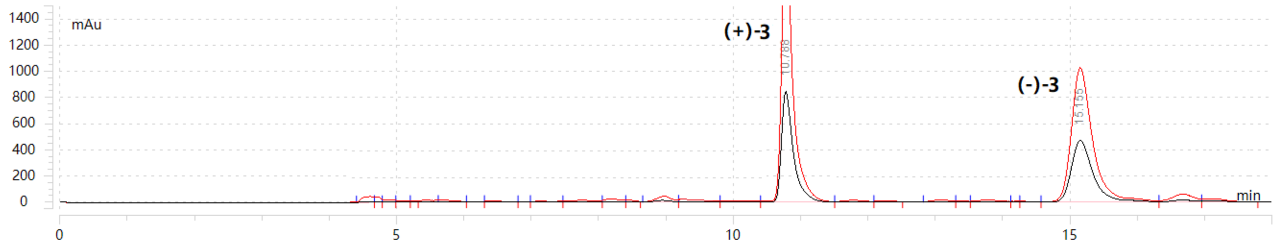

Figures S8. The chiral HPLC analysis of **3**.

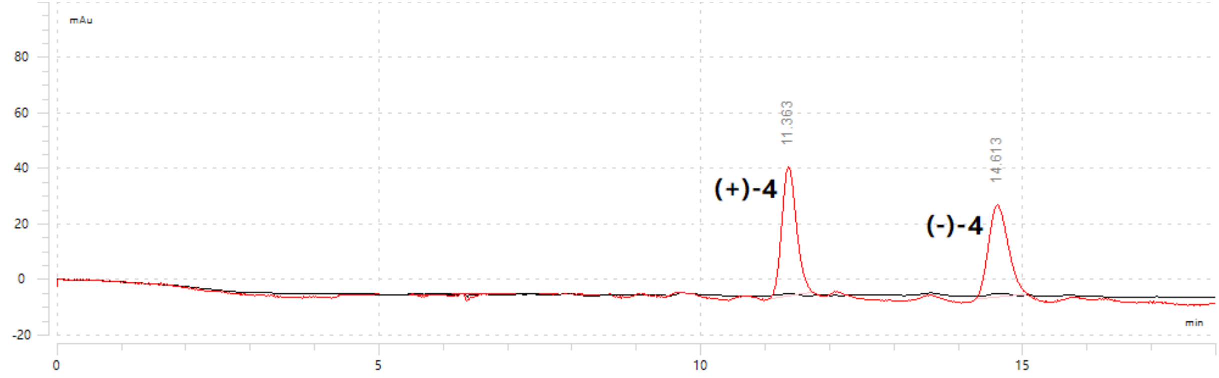

Figures S9. The chiral HPLC analysis of **4**.

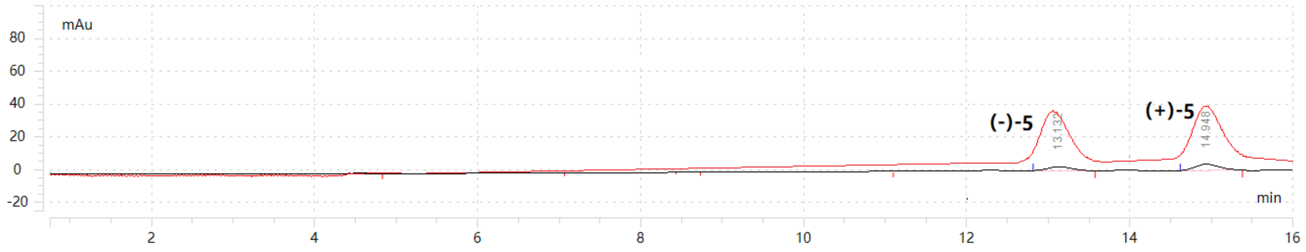

Figures S10. The chiral HPLC analysis of **5**.

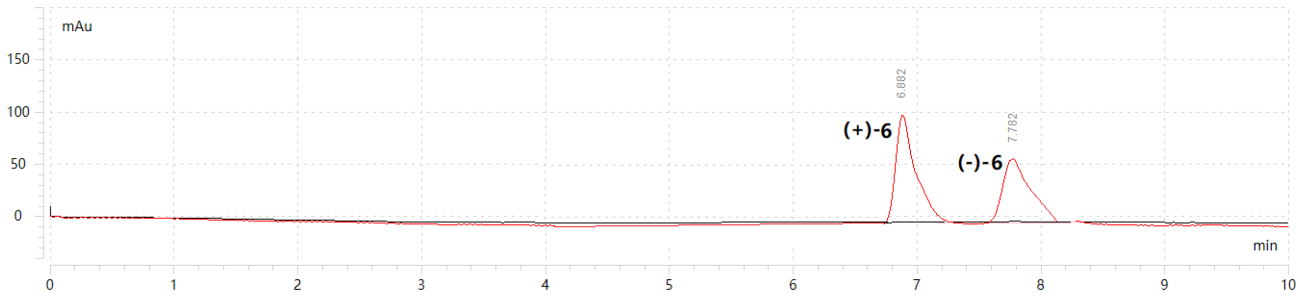

Figures S11. The chiral HPLC analysis of **6**.

3. X-ray crystallographic analysis of (–)-1

Table S1. Crystal data and structure refinement for (–)-**1**

Identification code	(–)-**1**
Empirical formula	$C_{21}H_{30}O_5$
Formula weight	362.45
Temperature/K	100.00(10)
Crystal system	orthorhombic
Space group	$P2_12_12_1$
a/Å	6.0081(2)
b/Å	15.4529(5)
c/Å	21.4935(8)
α/°	90
β/°	90
γ/°	90
Volume/$Å^3$	1995.51(12)
Z	4
$\rho_{calc}g/cm^3$	1.206
μ/mm^{-1}	0.687
F(000)	784.0
Crystal size/mm^3	0.12 × 0.1 × 0.09
Radiation	CuKα (λ = 1.54184)
2Θ range for data collection/°	7.046 to 147.142
Index ranges	$-5 \leq h \leq 7$, $-18 \leq k \leq 18$, $-26 \leq l \leq 25$
Reflections collected	12660
Independent reflections	3927 [R_{int} = 0.0485, R_{sigma} = 0.0547]
Data/restraints/parameters	3927/0/241
Goodness-of-fit on F^2	1.044
Final R indexes [I>=2σ (I)]	R_1 = 0.0448, wR_2 = 0.0986
Final R indexes [all data]	R_1 = 0.0549, wR_2 = 0.1047
Largest diff. peak/hole / e $Å^{-3}$	0.19/-0.23
Flack parameter	0.11(16)

4. Quantum chemical ^{13}C NMR calculations of 1

Table S2. Experimental chemical shifts and calculated unscaled shifts for PD4+ probability analysis for **1**[a].

Nuclie	Sp2?	Experi-mental	mPW1PW91/6-311G(d,p), PCM			
			Isomer 1	Isomer 2	Isomer 3	Isomer 4
C		95.4	97.07	98.61	96.45	96.75
C		70.2	70.84	76.28	77.43	65.86
C		68.8	82.73	75.43	83.54	79.32
C	x	117.8	116.57	115.77	119.32	116.76
C	x	149.1	149.40	149.71	147.01	149.35
C	x	113.3	112.90	114.18	113.08	112.13
C	x	122.5	121.73	120.92	121.34	121.23
C	x	143.4	141.59	143.14	140.14	141.61
C	x	124.1	122.19	123.77	122.52	124.63
C		30.4	31.89	34.84	36.20	34.69
C		25.9	26.93	33.67	27.79	29.48
C		32.0	32.87	36.71	33.21	34.90
C		22.8	25.55	27.89	26.97	26.84
C		14.2	14.96	16.11	15.85	16.34
C	x	122.2	122.60	121.29	122.60	123.66
C	x	132.4	131.17	127.68	130.45	131.33
C		77.4	78.96	82.26	79.10	78.61
C		27.4	26.82	29.58	29.64	29.85
C		28.0	29.68	29.71	27.11	27.39
C		55.8	56.34	56.06	54.95	55.88
C		53.9	56.55	56.48	60.21	50.75

[a] The chemical shifts were measured in chloroform-*d*, the unscaled shifts were calculated in chloroform. Isomer 1 is configuration 1*R**,3*R**,4*R**-**1**, isomer 2 is 1*R**,3*S**,4*S**-**1**, isomer 3 is 1*S**,3*S**,4*R**-**1**, and isomer 4 is 1*R**,3*S**,4*R**-**1**.

Table S3. Experimental chemical shifts and calculated unscaled shifts for PD4+ probability analysis for **2**[a].

Nuclie	Sp2?	Experi-mental	mPW1PW91/6-311G(d,p), PCM			
			Isomer 1	Isomer 2	Isomer 3	Isomer 4
C		95.2	97.07	98.61	96.45	96.75
C		64.8	70.84	76.28	77.43	65.86
C		76	82.73	75.43	83.54	79.32
C	x	117.8	116.57	115.77	119.32	116.76
C	x	149.4	149.40	149.71	147.01	149.35
C	x	113.4	112.90	114.18	113.08	112.13
C	x	122.5	121.73	120.92	121.34	121.23
C	x	143.0	141.59	143.14	140.14	141.61

C	x	124.2	122.19	123.77	122.52	124.63
C		31.9	31.89	34.84	36.20	34.69
C		25.2	26.93	33.67	27.79	29.48
C		32.0	32.87	36.71	33.21	34.90
C		22.8	25.55	27.89	26.97	26.84
C		14.2	14.96	16.11	15.85	16.34
C	x	122.1	122.60	121.29	122.60	123.66
C	x	132.2	131.17	127.68	130.45	131.33
C		77.4	78.96	82.26	79.10	78.61
C		27.6	26.82	29.58	29.64	29.85
C		28.0	29.68	29.71	27.11	27.39
C		56.1	56.34	56.06	54.95	55.88
C		51.4	56.55	56.48	60.21	50.75

[a] The chemical shifts were measured in chloroform-*d*, the unscaled shifts were calculated in chloroform. Isomer 1 is configuration 1*R**,3*R**,4*R**-**2**, isomer 2 is 1*R**,3*S**,4*S**-**2**, isomer 3 is 1*S**,3*S**,4*R**-**2**, and isomer 4 is 1*R**,3*S**,4*R**-**2**.

Table S4. Experimental chemical shifts and calculated unscaled shifts for PD4+ probability analysis for **4**[a].

Nuclie	Sp2?	Experi-mental	mPW1PW91/6-311G(d,p), PCM	
			Isomer 1	Isomer 2
C		95.3	97.85	97.71
C		70.1	72.49	71.81
C		68.7	72.62	71.99
C	x	117.3	115.16	115.98
C	x	150.5	148.55	148.37
C	x	112.8	114.77	114.46
C	x	116.2	119.93	121.34
C	x	149.3	143.10	141.76
C	x	128.8	124.74	124.60
C		30.8	33.26	33.03
C		26.0	28.62	28.62
C		32.0	33.47	34.25
C		30.4	26.67	26.30
C		14.2	15.09	15.75
C		22.8	27.96	30.10
C		88.4	87.92	79.43
C		76.1	78.68	78.78
C		22.2	25.57	27.18
C		19.6	25.12	21.00
C		55.8	55.75	55.78
C		50.2	53.35	53.67
C		53.8	56.24	56.49

[a] The chemical shifts were measured in chloroform-*d*, the unscaled shifts were calculated in chloroform. Isomer 1 is configuration 1is 1*R**,3*S**,4*S**,2′′*R**-**4**, and isomer 2 is 1*R**,3*S**,4*S**,2′′*S**-**4**.

Table S5. Experimental chemical shifts and calculated unscaled shifts for PD4+ probability analysis for **5**[a].

Nuclie	Sp2?	Experi-mental	mPW1PW91/6-311G(d,p), PCM	
			Isomer 1	Isomer 2
C		95.2	98.55	98.26
C		70.5	75.26	76.31
C		68.9	72.78	74.05
C	x	116.5	113.22	113.69
C	x	150.2	151.04	150.87
C	x	112.9	116.28	116.51
C	x	117.4	122.01	122.65
C	x	149.6	141.45	141.66
C	x	128.8	125.45	124.92
C		30.4	35.67	35.93
C		26.0	36.18	35.37
C		32.0	38.38	38.34
C		22.8	29.01	29.30
C		14.2	16.64	16.49
C		31.0	36.22	36.58
C		89.7	83.43	73.75
C		72.1	80.85	81.07
C		24.3	24.42	20.89
C		26.1	27.37	25.44
C		55.6	56.31	56.10
C		53.9	54.89	56.87

[a] The chemical shifts were measured in chloroform-*d*, the unscaled shifts were calculated in chloroform. Isomer 1 is configuration 1is 1*R**,3*S**,4*S**,2′′*R**-**5**, and isomer 2 is 1*R**,3*S**,4*S**,2′′*S**-**5**.

Table S6. Experimental chemical shifts and calculated unscaled shifts for PD4+ probability analysis for **6**[a].

Nuclie	Sp2?	Experi-mental	mPW1PW91/6-311G(d,p), PCM			
			Isomer 1	Isomer 2	Isomer 3	Isomer 4
C		96.6	102.74	104.67	92.29	103.23
C		72.6	76.92	80.79	66.69	77.41
C		70.9	75.11	77.25	64.96	76.96
C	x	117.9	127.92	125.66	112.34	128.32
C	x	151.4	161.90	163.38	142.49	159.96

C	x	112.9	124.43	128.21	106.33	122.84
C	x	123.0	136.03	135.34	118.88	134.36
C	x	150.9	152.76	153.27	135.96	153.03
C	x	131.3	135.04	135.65	119.09	134.88
C		31.9	37.98	39.35	29.04	38.02
C		26.8	31.79	40.50	24.55	32.49
C		33.0	38.65	42.47	30.67	38.40
C		23.7	30.35	32.45	22.00	30.14
C		14.4	18.62	20.14	11.28	17.88
C		74.0	77.67	73.68	67.95	74.84
C		98.3	98.77	80.15	72.48	83.67
C		72.0	87.02	88.96	75.35	86.39
C		25.4	28.92	25.21	22.97	22.93
C		25.8	27.21	28.46	14.06	30.61
C		58.4	59.94	60.95	50.35	60.22
C		49.6	58.59	60.18	48.93	58.77

[a] The chemical shifts were measured in methanol-d_4, the unscaled shifts were calculated in methanol. Isomer 1 is configuration 1*R**,3*S**,4*S**,1′′*R**,2′′*R**-**6**, isomer 2 is 1*R**,3*S**,4*S**,1′′*S**,2′′*S**-**6**, isomer 3 is 1*R**,3*S**,4*S**,1′′*R**,2′′*S**-**6**, and isomer 4 1*R**,3*S**,4*S**,1′′*S**,2′′*R**-**6**.

5. Quantum chemical ECD calculations of 1–6

Table S7. Cartesian coordinate of dominant conformer of **1** and **2** (B3LYP/6-31+g(d))

Cartesian coordinate of **1**							Cartesian coordinate of **2**					
Standard orientation							Standard orientation					
Center Number	Atomic Number	Atomic Type	Coordinates (Angstroms)				Center Number	Atomic Number	Atomic Type	Coordinates (Angstroms)		
			X	Y	Z					X	Y	Z
1	6	0	-1.785856	1.254584	-2.237779		1	6	0	-2.848329	2.329141	-0.170235
2	6	0	-2.464652	0.227331	-1.586587		2	6	0	-3.361792	1.035246	-0.22654
3	6	0	-2.008932	-0.205685	-0.31333		3	6	0	-2.46289	-0.059829	-0.291968
4	6	0	-0.890559	0.382569	0.291251		4	6	0	-1.070765	0.1319	-0.302422
5	6	0	-0.204847	1.418867	-0.371266		5	6	0	-0.555303	1.437708	-0.283909
6	6	0	-0.656501	1.84077	-1.630346		6	6	0	-1.454579	2.519321	-0.204062
7	6	0	-3.626502	-0.448147	-2.161122		7	6	0	-4.795275	0.747363	-0.235785
8	6	0	-4.302192	-1.357372	-1.447834		8	6	0	-5.230795	-0.516706	-0.168341
9	6	0	-3.939369	-1.71553	-0.030228		9	6	0	-4.293253	-1.69134	-0.053467
10	8	0	-2.587147	-1.242549	0.367155		10	8	0	-2.895122	-1.353083	-0.424456
11	6	0	-0.383221	-0.050619	1.639185		11	6	0	-0.13984	-1.054075	-0.344468
12	8	0	1.040403	-0.111474	1.726777		12	8	0	1.169544	-0.708683	-0.802778
13	6	0	1.841722	0.81124	0.945561		13	6	0	1.765537	0.411227	-0.095326
14	6	0	0.970243	1.973725	0.389107		14	6	0	0.927469	1.667342	-0.439
15	6	0	2.521192	0.011265	-0.169837		15	6	0	3.194625	0.500428	-0.628994
16	6	0	3.593743	-0.931842	0.384603		16	6	0	4.116678	-0.525695	0.039999

17	6	0	4.236668	-1.765059	-0.731257		17	6	0	5.546628	-0.431939	-0.507263
18	6	0	5.313787	-2.708497	-0.177658		18	6	0	6.472261	-1.457057	0.162994
19	6	0	5.957388	-3.542165	-1.283649		19	6	0	7.897752	-1.36825	-0.377404
20	8	0	1.803728	2.741113	-0.517933		20	8	0	1.375439	2.81955	0.31023
21	6	0	2.353262	3.943851	0.06191		21	6	0	1.320083	2.709148	1.751545
22	8	0	-0.813463	0.99162	2.534844		22	8	0	-0.026316	-1.485711	1.020263
23	6	0	-0.531712	0.732753	3.924952		23	6	0	0.655806	-2.746124	1.185403
24	8	0	-0.066325	2.835217	-2.362733		24	8	0	-1.059619	3.830492	-0.158668
25	6	0	-3.802278	-3.222445	0.168446		25	6	0	-4.216768	-2.194703	1.385909
26	6	0	-4.89231	-1.055859	0.964084		26	6	0	-4.611933	-2.788764	-1.064977
27	1	0	-2.10325	1.618387	-3.214797		27	1	0	-3.499029	3.200954	-0.110159
28	1	0	-3.901649	-0.183662	-3.182508		28	1	0	-5.473548	1.597893	-0.307296
29	1	0	-5.1691	-1.883092	-1.842675		29	1	0	-6.287952	-0.772995	-0.173396
30	1	0	-0.705268	-1.053681	1.984444		30	1	0	-0.446593	-1.870922	-1.028319
31	1	0	2.572134	1.192362	1.691962		31	1	0	1.72211	0.18606	0.993912
32	1	0	0.597905	2.615797	1.224499		32	1	0	1.142758	2.017181	-1.483028
33	1	0	2.959046	0.716707	-0.906669		33	1	0	3.579901	1.528879	-0.471504
34	1	0	1.754951	-0.569005	-0.7232		34	1	0	3.184511	0.335257	-1.726943
35	1	0	3.133422	-1.598944	1.142603		35	1	0	3.71104	-1.543971	-0.128307
36	1	0	4.370376	-0.353751	0.919942		36	1	0	4.121426	-0.375744	1.136482
37	1	0	4.678023	-1.097913	-1.496146		37	1	0	5.94516	0.589439	-0.354744
38	1	0	3.459673	-2.351913	-1.258492		38	1	0	5.542748	-0.591479	-1.603013
39	1	0	4.869779	-3.378266	0.58483		39	1	0	6.076668	-2.479724	0.005993
40	1	0	6.091854	-2.123431	0.35018		40	1	0	6.47811	-1.299523	1.259235
41	1	0	6.723695	-4.212616	-0.879524		41	1	0	7.932749	-1.55758	-1.456414
42	1	0	6.438431	-2.909177	-2.03783		42	1	0	8.551155	-2.103205	0.10569
43	1	0	5.217778	-4.163117	-1.801956		43	1	0	8.334111	-0.377798	-0.205161
44	1	0	1.553201	4.636289	0.341133		44	1	0	1.49736	3.740739	2.078204
45	1	0	2.985375	3.69649	0.919753		45	1	0	0.339221	2.352503	2.082243
46	1	0	2.959683	4.350582	-0.757631		46	1	0	2.120453	2.049659	2.098645
47	1	0	0.534233	0.522489	4.062303		47	1	0	0.042075	-3.556293	0.781859
48	1	0	-0.817399	1.672122	4.410503		48	1	0	1.638734	-2.711634	0.703728
49	1	0	-1.149362	-0.096369	4.281192		49	1	0	0.750591	-2.829715	2.273684
50	1	0	0.794232	3.150323	-1.925408		50	1	0	-0.048585	3.915754	-0.108578
51	1	0	-3.339217	-3.446123	1.139657		51	1	0	-3.965961	-1.379658	2.077869
52	1	0	-4.771799	-3.728046	0.132336		52	1	0	-5.158895	-2.640701	1.716495
53	1	0	-3.153364	-3.666798	-0.597544		53	1	0	-3.423807	-2.946942	1.493502
54	1	0	-5.910584	-1.445678	0.87687		54	1	0	-5.54157	-3.310796	-0.81947
55	1	0	-4.55269	-1.214874	1.99592		55	1	0	-4.704194	-2.379368	-2.079437
56	1	0	-4.932118	0.031083	0.81214		56	1	0	-3.804066	-3.532839	-1.09864

Table S8. Cartesian coordinate of dominant conformer of **3** and **4** (B3LYP/6-31+g(d))

Cartesian coordinate of **3**							Cartesian coordinate of **4**					
Standard orientation							Standard orientation					
Center	Atomic	Atomic	Coordinates (Angstroms)				Center	Atomic	Atomic	Coordinates (Angstroms)		

Number	Number	Type	X	Y	Z		Number	Number	Type	X	Y	Z
1	6	0	-1.975802	-1.149492	-2.076446		1	6	0	1.911771	2.328097	0.803504
2	6	0	-2.554877	-0.306555	-1.134997		2	6	0	2.676991	1.179529	0.614574
3	6	0	-1.932661	-0.170981	0.139958		3	6	0	2.062843	0.037683	0.047188
4	6	0	-0.778604	-0.880224	0.46427		4	6	0	0.691794	0.014468	-0.258391
5	6	0	-0.196928	-1.741854	-0.492019		5	6	0	-0.088633	1.155764	-0.023493
6	6	0	-0.795304	-1.85656	-1.751874		6	6	0	0.549878	2.31602	0.454941
7	6	0	-0.10756	-0.766228	1.806936		7	6	0	0.066493	-1.2071	-0.885195
8	8	0	1.317137	-0.712799	1.729336		8	8	0	-1.135574	-0.881766	-1.590078
9	6	0	2.000013	-1.450741	0.682604		9	6	0	-2.12377	-0.194967	-0.770618
10	6	0	1.038048	-2.455211	-0.015032		10	6	0	-1.571011	1.172637	-0.296571
11	6	0	2.582117	-0.429619	-0.299582		11	6	0	-3.360677	-0.108699	-1.664184
12	6	0	3.729024	0.368487	0.32858		12	6	0	-4.577672	-0.825695	-1.070519
13	6	0	4.275884	1.422083	-0.643193		13	6	0	-5.169385	-0.075579	0.130516
14	6	0	5.426112	2.219775	-0.014596		14	6	0	-6.405002	-0.794762	0.687405
15	6	0	5.97397	3.276155	-0.984521		15	6	0	-6.996832	-0.045432	1.889602
16	6	0	7.119426	4.073479	-0.364289		16	6	0	-8.228712	-0.755101	2.447172
17	8	0	1.744802	-2.999768	-1.161492		17	8	0	-1.901537	2.270757	-1.177915
18	8	0	-0.446009	-1.988539	2.485315		18	8	0	-0.279551	-2.068109	0.206778
19	8	0	-0.311184	-2.645324	-2.757481		19	8	0	-0.104611	3.512444	0.612781
20	6	0	-3.777592	0.445143	-1.383016		20	6	0	4.127905	1.157118	0.981479
21	6	0	-4.155083	1.382411	-0.492606		21	6	0	4.701951	-0.266384	0.964247
22	8	0	-2.474066	0.616094	1.138859		22	8	0	2.744581	-1.129259	-0.223415
23	6	0	-3.335475	1.743393	0.750244		23	6	0	4.21735	-1.071241	-0.277882
24	8	0	-5.263219	2.185788	-0.54346		24	8	0	6.135119	-0.198598	0.795328
25	6	0	-0.022538	-2.039031	3.86278		25	6	0	6.869416	-0.009857	2.019357
26	6	0	2.312789	-4.307713	-0.936377		26	6	0	-0.685331	-3.394522	-0.191013
27	6	0	-4.2011	1.918924	1.989391		27	6	0	-1.223713	2.265319	-2.456581
28	6	0	-2.429766	2.931192	0.446928		28	6	0	4.639154	-0.44152	-1.595675
29	6	0	-6.168035	2.007272	-1.664437		29	6	0	4.585654	-2.54533	-0.166331
30	1	0	-2.408738	-1.283233	-3.066541		30	1	0	2.343565	3.242066	1.20896
31	1	0	-0.36642	0.129504	2.406782		31	1	0	0.692291	-1.710967	-1.648935
32	1	0	2.79316	-1.992085	1.242383		32	1	0	-2.291331	-0.837355	0.129308
33	1	0	0.745811	-3.269596	0.691624		33	1	0	-2.138543	1.490736	0.617924
34	1	0	2.924095	-0.960669	-1.21249		34	1	0	-3.608265	0.953811	-1.876128
35	1	0	1.777484	0.257085	-0.631837		35	1	0	-3.109826	-0.555823	-2.651448
36	1	0	3.365979	0.856291	1.256784		36	1	0	-5.350216	-0.934449	-1.857771
37	1	0	4.541502	-0.313757	0.642349		37	1	0	-4.305009	-1.858586	-0.777935
38	1	0	4.620189	0.933033	-1.574669		38	1	0	-4.406073	0.029894	0.924951
39	1	0	3.462963	2.10963	-0.94729		39	1	0	-5.435548	0.957498	-0.167102
40	1	0	5.080718	2.708789	0.916585		40	1	0	-7.172003	-0.897139	-0.104099
41	1	0	6.239125	1.533879	0.291068		41	1	0	-6.140045	-1.827795	0.983852
42	1	0	6.320203	2.78764	-1.916257		42	1	0	-6.231859	0.054718	2.684412
43	1	0	5.161285	3.964529	-1.288564		43	1	0	-7.262879	0.988573	1.59466
44	1	0	7.502949	4.824823	-1.063291		44	1	0	-9.022169	-0.83696	1.695738
45	1	0	6.799303	4.600596	0.541621		45	1	0	-7.991425	-1.770312	2.784828

46	1	0	7.957886	3.424984	-0.086255		46	1	0	-8.643467	-0.213033	3.304363
47	1	0	0.578681	-3.064616	-2.499794		47	1	0	-0.979024	3.517394	0.105362
48	1	0	-4.340233	0.202935	-2.277855		48	1	0	4.280549	1.614389	1.980204
49	1	0	-0.59703	-1.32095	4.4547		49	1	0	4.710581	1.793837	0.277612
50	1	0	-0.263369	-3.06751	4.153118		50	1	0	4.447094	-0.811738	1.897915
51	1	0	1.052918	-1.846714	3.935437		51	1	0	6.689985	-0.83934	2.709442
52	1	0	2.813387	-4.520998	-1.889458		52	1	0	7.910659	-0.010902	1.672444
53	1	0	3.041286	-4.268999	-0.12123		53	1	0	6.61616	0.950175	2.477082
54	1	0	1.524537	-5.039162	-0.733091		54	1	0	0.171809	-3.938759	-0.597193
55	1	0	-4.682271	0.970525	2.269282		55	1	0	-1.018695	-3.837581	0.754299
56	1	0	-3.607362	2.242023	2.852811		56	1	0	-1.505639	-3.340021	-0.914029
57	1	0	-4.993768	2.65861	1.813064		57	1	0	-1.828838	2.951066	-3.057799
58	1	0	-3.012545	3.838358	0.247336		58	1	0	-0.204396	2.640081	-2.324069
59	1	0	-1.757003	3.137515	1.289352		59	1	0	-1.209798	1.257433	-2.887952
60	1	0	-1.792958	2.741001	-0.4273		60	1	0	4.36824	-1.069544	-2.451507
61	1	0	-6.578807	0.993306	-1.659706		61	1	0	4.166509	0.535623	-1.752549
62	1	0	-6.944388	2.751184	-1.446244		62	1	0	5.728704	-0.288283	-1.612932
63	1	0	-5.654606	2.233786	-2.603276		63	1	0	4.302536	-3.102253	-1.067314
							64	1	0	5.668118	-2.65805	-0.018679
							65	1	0	4.068058	-3.024836	0.675056

Table S9. Cartesian coordinate of dominant conformer of **5** and **6** (B3LYP/6-31+g(d))

Cartesian coordinate of **5**							Cartesian coordinate of **6**					
Standard orientation							Standard orientation					
Center Number	Atomic Number	Atomic Type	Coordinates (Angstroms) X	Y	Z		Center Number	Atomic Number	Atomic Type	Coordinates (Angstroms) X	Y	Z
1	6	0	-2.342091	2.362504	-0.687082		1	6	0	-2.31474	2.126359	-0.821478
2	6	0	-2.973962	1.139092	-0.473678		2	6	0	-2.853231	0.884494	-0.501859
3	6	0	-2.182796	0.009739	-0.159983		3	6	0	-1.991373	-0.147483	-0.071681
4	6	0	-0.779597	0.077782	-0.132927		4	6	0	-0.598205	0.023915	-0.033966
5	6	0	-0.141592	1.298233	-0.395024		5	6	0	-0.049271	1.262375	-0.396873
6	6	0	-0.940281	2.437914	-0.614642		6	6	0	-0.923401	2.315527	-0.732593
7	6	0	-4.464572	1.025317	-0.554319		7	6	0	-4.341225	0.644393	-0.621423
8	6	0	-4.932204	-0.436454	-0.553141		8	6	0	-4.776694	-0.600827	0.19507
9	6	0	-4.141064	-1.302091	0.472367		9	6	0	-3.799476	-1.788678	-0.005924
10	8	0	-2.714827	-1.233169	0.116217		10	8	0	-2.458296	-1.349674	0.426062
11	6	0	0.041171	-1.13607	0.227681		11	6	0	0.302641	-1.087775	0.444885
12	8	0	1.333633	-0.768753	0.719576		12	8	0	1.564483	-0.591254	0.895347
13	6	0	2.09058	0.068986	-0.198453		13	6	0	2.263472	0.219868	-0.090567
14	6	0	1.360781	1.417791	-0.423458		14	6	0	1.441617	1.49164	-0.420158
15	6	0	3.468464	0.192355	0.451016		15	6	0	3.627221	0.491161	0.543109
16	6	0	4.559127	-0.505324	-0.369327		16	6	0	4.772717	-0.135312	-0.25965
17	6	0	5.930075	-0.379109	0.308577		17	6	0	6.130598	0.141762	0.399634
18	6	0	7.027407	-1.072619	-0.510993		18	6	0	7.30024	-0.455202	-0.396276
19	6	0	8.394154	-0.950652	0.159085		19	6	0	7.38423	-1.975587	-0.280763

20	8	0	1.794158	2.469466	0.469112		20	8	0	1.782636	2.634415	0.394463
21	6	0	1.404627	2.309559	1.853621		21	6	0	1.386474	2.548516	1.783867
22	8	0	0.220369	-1.860066	-0.995202		22	8	0	0.529657	-1.917354	-0.702366
23	6	0	0.784825	-3.177559	-0.825987		23	6	0	1.227852	-3.147015	-0.413072
24	8	0	-0.41445	3.695423	-0.769104		24	8	0	-0.487059	3.588053	-0.998496
25	6	0	-4.437727	-2.784461	0.287701		25	6	0	-4.074954	-2.931105	0.961032
26	6	0	-4.297746	-0.837683	1.914733		26	6	0	-3.720173	-2.236303	-1.456938
27	8	0	-6.33311	-0.516006	-0.292278		27	8	0	-4.836271	-0.213611	1.572575
28	1	0	-2.90763	3.267549	-0.905691		28	8	0	-5.083667	1.771924	-0.171625
29	1	0	-4.932941	1.584922	0.282796		29	1	0	-2.960115	2.957912	-1.118124
30	1	0	-4.840122	1.525701	-1.474292		30	1	0	-4.637139	0.562255	-1.695798
31	1	0	-4.853988	-0.884304	-1.573711		31	1	0	-5.830102	-0.881893	-0.028928
32	1	0	-0.38242	-1.760315	1.039881		32	1	0	-0.079822	-1.658073	1.315309
33	1	0	2.121743	-0.476318	-1.174113		33	1	0	2.335163	-0.399453	-1.018441
34	1	0	1.702989	1.851091	-1.400963		34	1	0	1.76441	1.87508	-1.425089
35	1	0	3.725942	1.262559	0.602385		35	1	0	3.787941	1.585178	0.653159
36	1	0	3.419716	-0.248086	1.47108		36	1	0	3.622766	0.087274	1.579391
37	1	0	4.30281	-1.574886	-0.502519		37	1	0	4.618061	-1.228777	-0.349007
38	1	0	4.60331	-0.071791	-1.387319		38	1	0	4.772361	0.259079	-1.294189
39	1	0	6.185772	0.688921	0.449119		39	1	0	6.27915	1.234857	0.500651
40	1	0	5.890323	-0.814247	1.325773		40	1	0	6.136717	-0.258696	1.431897
41	1	0	6.774198	-2.141785	-0.650883		41	1	0	7.218514	-0.164801	-1.462129
42	1	0	7.070224	-0.636352	-1.52826		42	1	0	8.247947	-0.006614	-0.036486
43	1	0	8.693277	0.096976	0.278339		43	1	0	8.235026	-2.369873	-0.847281
44	1	0	8.397818	-1.407291	1.155335		44	1	0	7.505582	-2.293961	0.760862
45	1	0	9.170951	-1.447897	-0.432494		45	1	0	6.479409	-2.459495	-0.665619
46	1	0	1.546454	1.273944	2.183889		46	1	0	0.317762	2.767312	1.867079
47	1	0	2.079404	2.993403	2.378364		47	1	0	1.611272	1.557652	2.195265
48	1	0	0.359021	2.609998	1.968919		48	1	0	1.993101	3.326545	2.258675
49	1	0	1.727235	-3.119013	-0.271774		49	1	0	2.163339	-2.93753	0.116716
50	1	0	0.063871	-3.828748	-0.32394		50	1	0	0.582046	-3.813194	0.165396
51	1	0	0.951528	-3.501793	-1.859624		51	1	0	1.421128	-3.555342	-1.411466
52	1	0	0.548313	3.728805	-0.459606		52	1	0	0.456886	3.727098	-0.663504
53	1	0	-3.96422	-3.168148	-0.628279		53	1	0	-4.415667	-2.56294	1.940487
54	1	0	-4.052848	-3.389005	1.11633		54	1	0	-4.84301	-3.612357	0.579035
55	1	0	-5.519409	-2.958865	0.205761		55	1	0	-3.162146	-3.518767	1.137545
56	1	0	-3.585297	-1.363741	2.566431		56	1	0	-3.345792	-1.444671	-2.119584
57	1	0	-4.089153	0.231955	2.033003		57	1	0	-3.010334	-3.070649	-1.565637
58	1	0	-5.301795	-1.042564	2.300389		58	1	0	-4.688047	-2.572893	-1.842098
59	1	0	-6.556747	-0.119341	0.574513		59	1	0	-3.918937	-0.161863	1.945391
							60	1	0	-5.115245	1.783274	0.822595

Table S10. Key transitions and their related rotatory and oscillator strengths of dominant conformer of **1** at the B3LYP/6-31+g(d) level.

HOMO is 98

No.	Energy (cm-1)	Wavelength (nm)	R(length)	Osc. Strength	Major contribs
1	31683.07108	315.6259687	25.9821	0.188	HOMO->LUMO (95%)
2	38717.03226	258.2842593	7.2641	0.007	H-1->LUMO (26%), HOMO->L+2 (60%)
3	42819.16808	233.5402683	6.04	0.0054	HOMO->L+1 (65%), HOMO->L+3 (14%)
4	44523.41759	224.6009076	14.2536	0.655	H-1->LUMO (69%), HOMO->L+2 (27%)
5	46322.84052	215.8762262	2.0096	0.0064	HOMO->L+3 (16%), HOMO->L+4 (56%)
6	47802.8679	209.1924698	26.2184	0.2445	H-1->L+2 (64%)
7	48171.46327	207.591784	1.1449	0.0177	HOMO->L+3 (35%), HOMO->L+5 (31%)
8	48819.12648	204.8377495	12.5402	0.0402	HOMO->L+5 (37%), HOMO->L+7 (18%)
9	50516.117	197.9566244	33.5187	0.0194	HOMO->L+1 (12%), HOMO->L+6 (11%), HOMO->L+8 (15%), HOMO->L+10 (12%), HOMO->L+11 (11%)
10	51016.9873	196.0131425	-19.2966	0.0408	H-2->LUMO (69%)
11	51432.36283	194.4301107	-8.2791	0.0019	H-1->L+1 (13%), HOMO->L+8 (29%), HOMO->L+13 (13%)
12	51944.52489	192.5130708	-5.1114	0.0161	HOMO->L+4 (14%), HOMO->L+9 (27%)
13	52520.40475	190.4021884	-29.1605	0.0232	H-3->LUMO (11%), H-1->L+1 (24%), HOMO->L+9 (13%)
14	52602.67331	190.1044067	2.3638	0.0084	H-3->LUMO (58%)
15	53370.51312	187.3693809	-57.4406	0.1958	HOMO->L+12 (12%), HOMO->L+13 (11%), HOMO->L+20 (11%), HOMO->L+23 (10%)
16	53705.23321	186.2015934	3.7092	0.0155	HOMO->L+10 (26%), HOMO->L+11 (18%), HOMO->L+16 (17%)
17	54665.83953	182.9295971	9.477	0.0462	H-5->LUMO (10%), H-4->LUMO (38%)
18	54930.38939	182.0485912	-4.3338	0.0361	HOMO->L+11 (21%), HOMO->L+16 (17%)
19	55225.58831	181.0754816	-4.9198	0.0129	H-1->L+5 (16%), HOMO->L+8 (10%), HOMO->L+15 (21%)
20	56312.01712	177.5819889	-3.2207	0.0155	H-1->L+4 (20%), HOMO->L+13 (13%)
21	56456.39037	177.1278669	-15.4815	0.0116	H-1->L+3 (17%), H-1->L+4 (20%)
22	56642.70444	176.5452427	-2.4206	0.0007	H-8->LUMO (13%), H-6->LUMO (18%), H-5->LUMO (11%), H-4->LUMO (12%)
23	57153.25339	174.9681673	-9.1858	0.0048	H-5->LUMO (33%)
24	57208.09909	174.8004244	-4.0434	0.0011	HOMO->L+17 (25%)
25	57373.44275	174.2966697	29.0971	0.0263	H-1->L+3 (24%)
26	57629.12051	173.5233839	-0.4752	0.0131	H-1->L+5 (20%), HOMO->L+12 (17%)
27	57900.92935	172.7087995	-13.5094	0.0258	HOMO->L+6 (20%), HOMO->L+7 (15%), HOMO->L+20 (14%)
28	58194.51516	171.8375	-6.4838	0.0094	HOMO->L+9 (11%), HOMO->L+10 (16%), HOMO->L+16 (28%)
29	58596.17927	170.6595912	-10.1377	0.0236	H-2->L+2 (28%)
30	58733.29352	170.2611824	-0.8904	0.0503	
31	58787.33267	170.104673	-6.7732	0.0046	
32	58896.21751	169.7901906	27.5159	0.0061	HOMO->L+23 (13%)
33	59064.78739	169.3056124	10.2424	0.0087	HOMO->L+19 (30%)
34	59370.47152	168.4338989	0.1245	0.0013	HOMO->L+20 (17%)
35	59864.08283	167.0450716	-14.679	0.0052	HOMO->L+19 (14%)
36	60181.86527	166.163012	0.7417	0.0018	H-3->L+2 (24%)
37	60499.64772	165.2902187	-12.0204	0.036	
38	60618.21122	164.9669266	-4.5242	0.0089	
39	61019.87533	163.8810297	-24.6255	0.0157	
40	61118.27497	163.6171833	56.0948	0.0543	
41	61337.65777	163.0319833	-0.0829	0.0115	H-1->L+9 (12%)
42	61588.4962	162.3679846	-1.9993	0.003	
43	61723.19079	162.0136592	3.0827	0.0096	H-10->LUMO (20%), H-9->LUMO (19%)
44	61917.57041	161.5050451	3.8611	0.0118	H-4->L+2 (17%), H-3->L+2 (10%)
45	62067.58953	161.1146828	4.7362	0.0261	H-9->LUMO (10%)

46	62342.62459	160.4038981	3.638	0.0053	HOMO->L+24 (16%)
47	62424.89314	160.1925049	-17.2921	0.015	HOMO->L+24 (10%)
48	62924.15033	158.9214943	-7.4716	0.0016	H-2->L+3 (10%), HOMO->L+27 (11%)
49	63013.67787	158.6957044	-0.2801	0.0034	H-10->LUMO (21%), H-6->LUMO (11%)
50	63195.95918	158.2379654	-27.66	0.0186	H-1->L+10 (15%)

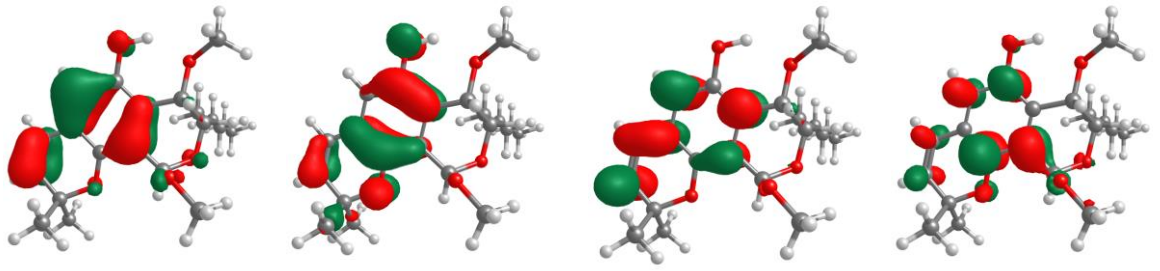

97 **98 (HOMO)** **99 (LUMO)** **101**

Figure S12. Key molecular orbitals involved in important transitions regarding the ECD spectrum of dominant conformer of **1**.

Table S11. Key transitions and their related rotatory and oscillator strengths of dominant conformer of **2** at the B3LYP/6-31+g(d) level.

HOMO is 98					
**No.**	**Energy (cm-1)**	**Wavelength (nm)**	**R(length)**	**Osc. Strength**	**Major contribs**
1	28998.05139	344.850758	-6.3409	0.1401	HOMO->LUMO (96%)
2	36312.6935	275.3857959	3.0223	0.0027	H-1->LUMO (25%), HOMO->L+1 (70%)
3	39030.78193	256.2080365	1.7541	0.0083	HOMO->L+2 (91%)
4	39791.36275	251.3108199	-20.6663	0.342	H-2->LUMO (11%), H-1->LUMO (55%), HOMO->L+1 (19%)
5	40877.79157	244.6316109	-14.6935	0.0247	HOMO->L+3 (81%)
6	41905.34191	238.6330607	23.5118	0.1793	H-2->LUMO (75%)
7	42656.24409	234.4322669	-1.8878	0.0028	HOMO->L+4 (67%), HOMO->L+5 (17%)
8	42913.53495	233.0267132	1.5515	0.0054	HOMO->L+4 (18%), HOMO->L+5 (74%)
9	44461.3129	224.9146359	24.2761	0.0926	H-1->L+1 (24%), HOMO->L+7 (45%), HOMO->L+8 (20%)
10	45126.7203	221.5982002	-20.7371	0.0583	H-1->L+1 (27%), HOMO->L+6 (11%), HOMO->L+8 (21%), HOMO->L+9 (23%)
11	45321.90647	220.6438515	-8.0537	0.0799	H-1->L+1 (32%), HOMO->L+6 (17%), HOMO->L+7 (26%)
12	45502.57467	219.767784	2.5571	0.0094	H-3->LUMO (97%)
13	45982.47455	217.4741594	0.8334	0.0111	HOMO->L+6 (20%), HOMO->L+7 (12%), HOMO->L+8 (36%), HOMO->L+9 (18%)
14	46392.2042	215.5534571	-0.9298	0.0423	HOMO->L+6 (13%), HOMO->L+9 (35%), HOMO->L+10 (33%)
15	47244.73223	211.6638094	-3.7611	0.0391	HOMO->L+6 (24%), HOMO->L+9 (13%), HOMO->L+10 (40%)
16	47606.87517	210.0536942	3.0954	0.017	H-4->LUMO (59%), HOMO->L+11 (23%)
17	47672.20608	209.7658326	-8.3753	0.043	H-4->LUMO (14%), H-1->L+2 (30%), HOMO->L+10 (13%), HOMO->L+11 (29%)
18	47706.08137	209.6168814	3.5451	0.0338	H-4->LUMO (17%), H-1->L+2 (56%), HOMO->L+11 (15%)
19	48572.32082	205.878571	1.5578	0.0142	HOMO->L+12 (83%)
20	48679.59256	205.4248911	-0.655	0.0053	H-2->L+1 (89%)
21	49446.62582	202.2382687	0.7726	0.0121	H-1->L+3 (56%), HOMO->L+13 (17%)
22	49724.08055	201.1098021	0.8261	0.0319	H-5->LUMO (77%)

23	49836.19161	200.6573873	11.242	0.0178	HOMO->L+13 (32%), HOMO->L+15 (17%), HOMO->L+16 (20%)
24	49894.26353	200.4238422	18.505	0.0242	H-1->L+3 (19%), HOMO->L+13 (14%), HOMO->L+14 (39%)
25	50334.64225	198.6703303	3.7018	0.0633	H-1->L+4 (10%), HOMO->L+15 (20%), HOMO->L+16 (42%)
26	50761.30954	197.0004338	-8.1455	0.0167	HOMO->L+13 (10%), HOMO->L+14 (38%), HOMO->L+15 (33%)
27	51000.85621	196.0751396	0.477	0.0084	H-1->L+4 (38%), H-1->L+5 (21%), HOMO->L+16 (14%)
28	51194.42927	195.333753	-1.49	0.0026	H-7->LUMO (65%), H-6->LUMO (12%)
29	51532.37558	194.0527656	-10.671	0.0082	H-1->L+4 (21%), H-1->L+5 (35%), HOMO->L+17 (27%)
30	51592.86716	193.8252427	-0.3447	0.0014	H-1->L+5 (25%), HOMO->L+17 (52%)
31	51995.33782	192.3249356	2.6798	0.008	H-3->L+1 (96%)
32	52187.29777	191.617509	-10.2574	0.0148	HOMO->L+19 (74%)
33	52497.82123	190.4840956	6.3065	0.0057	HOMO->L+18 (60%), HOMO->L+20 (15%)
34	52813.99057	189.3437684	-3.5108	0.0014	H-2->L+2 (49%), H-2->L+3 (31%)
35	53034.17992	188.5576437	6.7687	0.0437	H-1->L+7 (49%), H-1->L+8 (12%)
36	53281.79213	187.6813748	14.5766	0.0166	H-8->LUMO (11%), H-6->LUMO (15%), HOMO->L+18 (14%),<br>HOMO->L+20 (34%), HOMO->L+21 (10%)
37	53313.24776	187.57064	-2.2554	0.0007	H-8->LUMO (22%), H-6->LUMO (32%), HOMO->L+20 (11%)
38	53672.97103	186.313517	-0.2336	0.0001	H-1->L+6 (24%), H-1->L+8 (29%), H-1->L+9 (13%)
39	54198.03797	184.5085242	8.9401	0.0076	H-2->L+2 (25%), H-2->L+3 (42%)
40	54288.37206	184.201508	2.8933	0.0046	H-1->L+8 (15%), H-1->L+9 (18%), HOMO->L+21 (20%)
41	54462.58782	183.6122814	-0.3165	0.0112	H-1->L+8 (13%), H-1->L+9 (10%), HOMO->L+21 (35%)
42	54732.78355	182.7058547	-1.7545	0.0169	H-1->L+6 (10%), H-1->L+9 (13%), H-1->L+10 (30%), H-1->L+11 (11%)
43	54969.91055	181.9177055	24.5997	0.0518	H-4->L+1 (23%), HOMO->L+23 (44%), HOMO->L+25 (13%)
44	55057.82499	181.6272256	-5.1399	0.0052	H-8->LUMO (38%), H-6->LUMO (24%)
45	55178.80815	181.228996	-9.7907	0.059	H-4->L+1 (36%), HOMO->L+22 (12%), HOMO->L+23 (27%)
46	55217.52276	181.101931	4.4159	0.0129	HOMO->L+20 (10%), HOMO->L+22 (58%)
47	55532.88555	180.0734808	-11.5014	0.0239	H-10->LUMO (12%), H-1->L+6 (14%), H-1->L+9 (11%), H-1->L+11 (13%)
48	55724.03895	179.4557643	12.8988	0.0057	H-3->L+2 (28%), H-2->L+4 (14%), H-2->L+5 (20%)
49	55838.56967	179.0876818	-1.3228	0.0094	H-3->L+2 (17%), HOMO->L+25 (31%)
50	55882.93017	178.94552	2.0251	0.0054	H-3->L+2 (30%), H-2->L+5 (12%), HOMO->L+25 (14%)

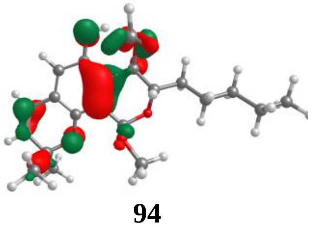

94

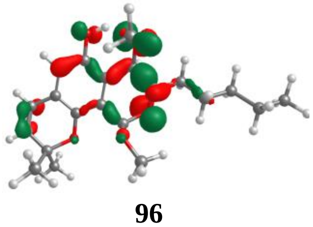

96

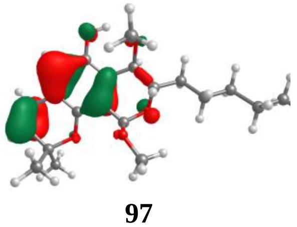

97

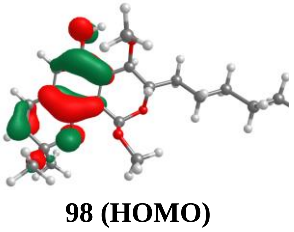

98 (HOMO)

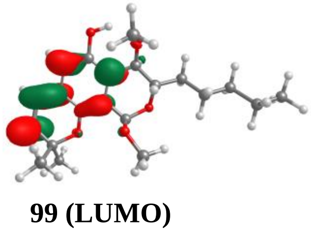

99 (LUMO)

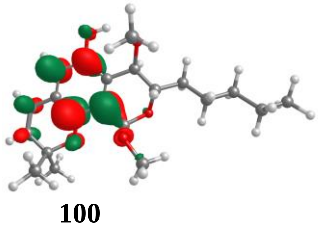

100

Figure S13. Key molecular orbitals involved in important transitions regarding the ECD spectrum of dominant conformer of **2**.

Table S12. Key transitions and their related rotatory and oscillator strengths of dominant conformer of **3** at the B3LYP/6-31+g(d) level.

HOMO is 110					
**No.**	**Energy (cm^{-1})**	**Wavelength (nm)**	**R(length)**	**Osc. Strength**	**Major contribs**
1	33272.78986	300.5458828	-16.4898	0.2792	HOMO->LUMO (91%)
2	38708.16016	258.3434594	-18.4822	0.056	H-1->LUMO (29%), HOMO->L+2 (44%), HOMO->L+3 (13%)
3	42405.40566	235.8189916	-2.4042	0.0026	HOMO->L+1 (70%)
4	43930.60008	227.6317642	32.0094	0.383	H-1->LUMO (62%), HOMO->L+2 (24%)
5	45885.68802	217.9328769	8.2457	0.0312	H-1->L+1 (10%), HOMO->L+4 (58%)
6	47026.15598	212.6476169	22.9836	0.3549	H-1->L+2 (42%), H-1->L+3 (15%), HOMO->L+5 (11%)
7	47327.80734	211.2922732	-3.4325	0.1507	H-1->L+2 (20%), HOMO->L+3 (16%), HOMO->L+5 (27%)
8	48009.34583	208.2927777	-15.7391	0.0495	HOMO->L+3 (24%), HOMO->L+5 (23%), HOMO->L+6 (11%), HOMO->L+8 (12%)
9	49508.73052	201.9845772	2.4804	0.0029	H-1->L+1 (20%), HOMO->L+6 (10%), HOMO->L+7 (26%)
10	50208.01321	199.1713944	-14.3541	0.0078	H-1->L+1 (17%), HOMO->L+3 (14%)
11	50382.22896	198.4826834	2.0831	0.0022	HOMO->L+10 (16%), HOMO->L+11 (15%)
12	51425.91039	194.454506	7.9184	0.0143	H-2->LUMO (68%)
13	51936.45935	192.5429674	-7.6382	0.0029	HOMO->L+8 (10%), HOMO->L+9 (24%), HOMO->L+10 (23%)
14	52736.56134	189.621768	-21.4953	0.042	H-1->L+5 (17%), HOMO->L+13 (28%)
15	53210.81534	187.9317191	6.1381	0.1379	HOMO->L+16 (29%)
16	53387.45076	187.3099363	7.6787	0.0203	HOMO->L+10 (15%), HOMO->L+11 (25%)
17	53959.29785	185.3248726	7.6281	0.0109	H-3->LUMO (42%)
18	54215.78216	184.4481367	-13.5517	0.0374	H-3->LUMO (33%)
19	54485.17134	183.5361761	7.1092	0.0098	H-1->L+4 (17%), H-1->L+13 (10%)
20	54856.99293	182.2921649	7.0229	0.0044	H-1->L+4 (31%), HOMO->L+13 (11%)
21	55501.42992	180.1755381	19.6245	0.0166	H-1->L+5 (14%), HOMO->L+12 (11%), HOMO->L+14 (10%)
22	55805.50094	179.193804	-4.3462	0.0456	H-4->LUMO (57%), HOMO->L+12 (11%)
23	55936.96931	178.7726458	-14.3298	0.0053	HOMO->L+13 (13%), HOMO->L+17 (12%), HOMO->L+18 (13%)
24	56285.40083	177.6659641	-7.9894	0.0073	H-4->LUMO (10%), H-1->L+3 (17%), HOMO->L+14 (10%)
25	56629.79957	176.585474	0.1192	0.0034	HOMO->L+19 (10%), HOMO->L+20 (17%), HOMO->L+23 (18%)
26	57036.303	175.3269317	1.8813	0.0112	
27	57413.77047	174.1742428	4.6854	0.0065	HOMO->L+12 (10%), HOMO->L+14 (14%), HOMO->L+15 (17%)
28	57614.60253	173.5671091	-29.7819	0.0492	H-2->L+3 (11%)
29	57792.0445	173.0341968	19.6693	0.0296	H-2->L+2 (26%)
30	57892.05725	172.7352676	3.0617	0.0061	H-5->LUMO (14%), HOMO->L+6 (11%), HOMO->L+18 (15%)
31	57994.48966	172.430175	8.3687	0.0513	H-5->LUMO (37%)
32	58079.98443	172.1763547	-0.6829	0.0049	HOMO->L+22 (23%)
33	58519.5566	170.8830446	-10.0789	0.0127	HOMO->L+6 (10%), HOMO->L+7 (14%)

34	58684.0937	170.4039267	12.7206	0.0142	H-2->L+1 (15%)
35	59006.71547	169.4722358	-4.1209	0.0088	H-7->LUMO (16%)
36	59151.89527	169.0562907	-4.63	0.0027	
37	59389.02227	168.3812869	-1.3606	0.0179	HOMO->L+20 (11%), HOMO->L+23 (12%)
38	59497.90712	168.0731388	-0.6572	0.0097	
39	59709.22438	167.4783102	20.5784	0.013	
40	60028.61993	166.5872048	7.1102	0.0051	H-1->L+10 (10%)
41	60220.57989	166.0561891	7.0263	0.0081	HOMO->L+25 (15%), HOMO->L+26 (12%)
42	60589.17526	165.0459832	-3.5612	0.0058	HOMO->L+21 (11%), HOMO->L+22 (15%), HOMO->L+24 (23%)
43	60759.35825	164.5837002	-2.2869	0.0018	H-1->L+10 (13%)
44	60897.27905	164.2109493	-9.8554	0.0219	H-3->L+2 (24%)
45	61045.68507	163.8117418	-0.109	0.0107	H-1->L+11 (17%)
46	61150.53714	163.5308612	21.7785	0.0214	H-8->LUMO (40%)
47	61236.83847	163.3003965	1.538	0.0015	
48	61439.28363	162.7623144	-19.0445	0.0134	
49	61982.90131	161.3348163	13.5891	0.0039	HOMO->L+27 (15%)
50	62172.44161	160.8429674	-0.2288	0.0219	

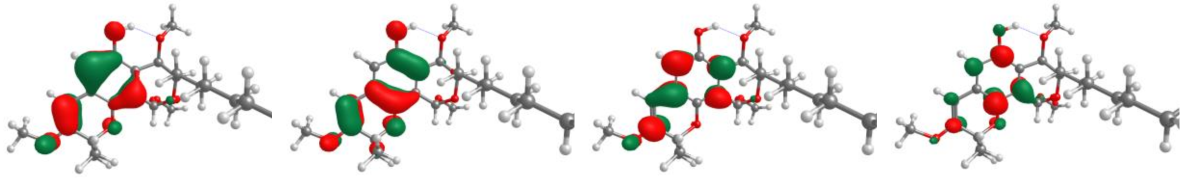

109 **110 (HOMO)** **111 (LUMO)** **113**

Figure S14. Key molecular orbitals involved in important transitions regarding the ECD spectrum of dominant conformer of **3**.

Table S13. Key transitions and their related rotatory and oscillator strengths of dominant conformer of **4** at the B3LYP/6-31+g(d) level.

HOMO is 111					
**No.**	**Energy (cm-1)**	**Wavelength (nm)**	**R(length)**	**Osc. Strength**	**Major contribs**
1	35936.83914	278.2659867	-8.3259	0.1626	HOMO->LUMO (89%)
2	43905.5969	227.7613951	14.2247	0.0472	H-1->LUMO (17%), HOMO->L+1 (10%), HOMO->L+2 (28%), HOMO->L+5 (16%), HOMO->L+6 (10%)
3	44522.61104	224.6049764	5.6483	0.0064	HOMO->L+1 (60%), HOMO->L+3 (12%)
4	48460.20976	206.3548641	-11.8262	0.0654	H-1->LUMO (23%), HOMO->L+2 (38%)
5	49934.59125	200.2619777	34.0402	0.231	H-1->LUMO (16%), HOMO->L+4 (27%), HOMO->L+10 (15%)
6	50129.77743	199.4822342	38.9163	0.2521	H-1->LUMO (33%), HOMO->L+3 (22%), HOMO->L+4 (13%), HOMO->L+6 (12%)
7	50416.9108	198.346147	-11.8505	0.0417	H-2->LUMO (87%)
8	50837.12566	196.7066365	-31.8741	0.1124	HOMO->L+3 (11%), HOMO->L+5 (27%), HOMO->L+6 (14%), HOMO->L+8 (11%)
9	52849.47896	189.2166242	-58.2449	0.1649	HOMO->L+5 (14%), HOMO->L+10 (11%), HOMO->L+11 (16%)
10	53653.61373	186.3807357	112.4306	0.424	H-1->L+2 (17%), H-1->L+5 (14%), H-1->L+6 (10%)
11	54528.72528	183.3895795	-3.1878	0.0093	H-1->L+1 (15%), HOMO->L+3 (14%), HOMO->L+6 (13%), HOMO->L+7 (10%), HOMO->L+9 (12%)

12	54702.94104	182.8055276	-0.5642	0.0107	HOMO->L+8 (18%), HOMO->L+9 (28%), HOMO->L+11 (13%), HOMO->L+15 (10%)
13	54944.90737	182.0004888	-1.1515	0.0031	H-1->L+1 (38%), HOMO->L+6 (10%)
14	55753.88146	179.3597098	10.8271	0.0141	HOMO->L+13 (35%)
15	55960.35939	178.6979231	0.4908	0.0123	HOMO->L+5 (10%), HOMO->L+8 (18%), HOMO->L+9 (16%)
16	56132.15549	178.1510066	13.4627	0.0128	H-3->LUMO (62%)
17	56473.32801	177.0747422	9.3418	0.0077	H-3->LUMO (16%), H-2->L+1 (21%), H-2->L+2 (27%)
18	56704.00258	176.3543938	-6.7402	0.0068	H-4->LUMO (76%)
19	57574.27481	173.6886836	8.5868	0.0089	H-3->L+1 (16%), H-3->L+2 (16%), H-3->L+3 (10%), H-3->L+5 (16%)
20	57700.90385	173.3075105	-6.4239	0.0024	H-5->LUMO (17%), HOMO->L+12 (16%)
21	58031.59117	172.3199347	-7.6224	0.0035	H-5->LUMO (26%)
22	58253.39363	171.6638186	2.2957	0.0022	H-5->LUMO (21%), HOMO->L+13 (15%), HOMO->L+15 (10%)
23	58818.78829	170.0137029	-2.7985	0.0015	HOMO->L+12 (29%), HOMO->L+14 (10%)
24	58935.73868	169.6763326	0.3677	0.0025	H-2->L+1 (35%), H-2->L+5 (22%)
25	59347.08144	168.5002827	1.7368	0.0022	HOMO->L+15 (14%), HOMO->L+16 (16%)
26	59895.53845	166.9573437	-6.2869	0.0039	H-7->LUMO (15%), H-1->L+5 (11%)
27	60031.0396	166.5804901	7.5711	0.0121	H-7->LUMO (12%), H-1->L+3 (11%)
28	60074.59354	166.4597197	8.086	0.0029	H-7->LUMO (12%)
29	60181.05872	166.1652389	-6.1401	0.0066	H-1->L+2 (20%), H-1->L+4 (14%)
30	60692.41423	164.7652368	-16.6135	0.0056	H-7->LUMO (11%), H-2->L+3 (23%)
31	60861.79066	164.3067003	19.2151	0.0213	HOMO->L+7 (19%), HOMO->L+15 (14%)
32	61107.78976	163.6452577	-5.1219	0.0112	HOMO->L+17 (19%), HOMO->L+22 (10%)
33	61148.11748	163.5373322	-0.0455	0.0159	H-4->L+1 (11%), H-1->L+2 (11%), H-1->L+4 (19%)
34	61250.54989	163.2638404	-8.3856	0.0166	H-8->LUMO (40%)
35	61623.98459	162.2744791	3.3612	0.0011	HOMO->L+7 (32%), HOMO->L+17 (12%), HOMO->L+22 (12%)
36	61710.28592	162.0475396	-16.1249	0.0073	H-8->LUMO (11%), H-4->L+1 (12%)
37	61838.52807	161.7114817	-4.0523	0.0118	H-3->L+6 (15%)
38	62338.59182	160.4142748	-10.1782	0.0025	H-2->L+4 (12%)
39	62480.5454	160.0498193	11.2909	0.0044	H-2->L+4 (15%)
40	62690.24955	159.5144392	16.7641	0.0075	H-3->L+2 (10%), H-3->L+5 (16%), H-3->L+8 (14%)
41	62801.55406	159.2317284	-2.6203	0.0065	HOMO->L+15 (12%), HOMO->L+21 (15%), HOMO->L+24 (14%)
42	63232.25412	158.1471377	3.6241	0.0013	
43	63477.44667	157.5362672	-8.4756	0.0044	
44	63591.9774	157.2525405	2.2774	0.0076	HOMO->L+19 (26%)
45	63724.25233	156.9261252	7.5253	0.0023	H-3->L+4 (10%), H-3->L+5 (17%), H-3->L+6 (10%)
46	63843.62238	156.6327164	-18.5813	0.0162	
47	64123.49677	155.9490749	-2.0665	0.0051	
48	64159.79172	155.8608551	-4.2188	0.0014	
49	64370.30242	155.3511421	-10.1489	0.0099	H-3->L+1 (10%), H-3->L+12 (14%)
50	64471.12173	155.1082055	-9.0334	0.0092	H-4->L+3 (16%)

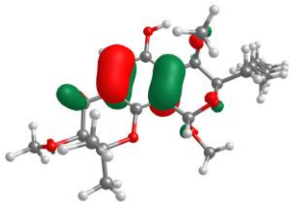

110

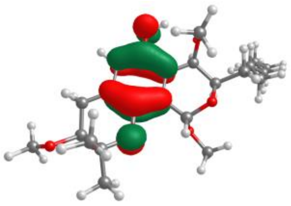

111 (HOMO)

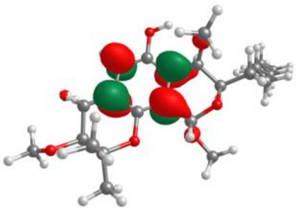

112 (LUMO)

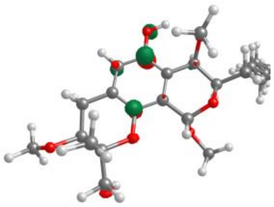

114

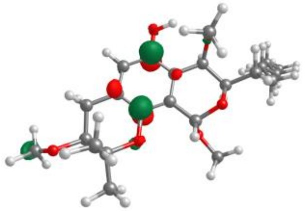

117

Figure S15. Key molecular orbitals involved in important transitions regarding the ECD spectrum of dominant conformer of **4**.

Table S14. Key transitions and their related rotatory and oscillator strengths of dominant conformer of **5** at the B3LYP/6-31+g(d) level.

HOMO is 103					
**No.**	**Energy (cm-1)**	**Wavelength (nm)**	**R(length)**	**Osc. Strength**	**Major contribs**
1	35936.83914	278.2659867	-7.7812	0.1582	HOMO->LUMO (89%)
2	43928.18042	227.6443027	12.5697	0.0457	H-1->LUMO (17%), HOMO->L+1 (11%), HOMO->L+2 (32%), HOMO->L+5 (22%)
3	44658.11218	223.9234825	7.2011	0.0074	HOMO->L+1 (59%), HOMO->L+5 (21%)
4	48477.95396	206.2793329	-16.3485	0.06	H-1->LUMO (22%), HOMO->L+2 (38%)
5	49988.6304	200.0454887	43.106	0.3232	H-1->LUMO (27%), HOMO->L+3 (14%), HOMO->L+5 (12%), HOMO->L+10 (19%)
6	50162.0396	199.3539354	6.4831	0.0209	H-2->LUMO (61%)
7	50216.8853	199.1362057	3.3825	0.2125	H-2->LUMO (27%), H-1->LUMO (22%), HOMO->L+3 (15%)
8	50940.36463	196.3079784	-30.9938	0.0659	HOMO->L+4 (40%), HOMO->L+8 (30%)
9	52526.05063	190.3817226	-46.2897	0.0861	HOMO->L+2 (12%), HOMO->L+5 (21%), HOMO->L+10 (17%)
10	53603.60735	186.5546088	57.9395	0.4155	H-1->L+2 (18%), H-1->L+5 (26%), HOMO->L+8 (15%)
11	53841.54091	185.730197	41.0632	0.0956	HOMO->L+6 (16%), HOMO->L+7 (11%), HOMO->L+8 (11%), HOMO->L+10 (15%)
12	54530.33839	183.3841545	0.0755	0.0041	HOMO->L+3 (19%), HOMO->L+6 (10%), HOMO->L+7 (15%), HOMO->L+11 (14%)
13	55141.70665	181.3509339	-0.0956	0.0027	H-1->L+1 (53%)
14	55605.47544	179.8384048	-5.1732	0.0238	HOMO->L+9 (10%), HOMO->L+12 (43%)
15	56168.45044	178.0358889	11.1134	0.0094	H-2->L+1 (19%), H-2->L+2 (35%)
16	56514.46229	176.9458577	2.2486	0.0132	H-4->LUMO (29%), H-3->LUMO (18%), HOMO->L+9 (15%)
17	56631.41268	176.5804441	2.3289	0.003	H-4->LUMO (25%), H-3->LUMO (10%), HOMO->L+9 (15%)
18	57283.10866	174.5715314	9.7993	0.0046	H-5->LUMO (12%), H-4->LUMO (14%), H-3->LUMO (40%)
19	57849.30987	172.8629092	1.4311	0.0059	HOMO->L+11 (20%)
20	58433.25527	171.1354254	2.4614	0.0005	H-5->LUMO (28%)
21	58565.5302	170.7489024	-3.8247	0.0034	H-5->LUMO (17%), HOMO->L+14 (19%)
22	58693.77235	170.3758269	4.7343	0.0151	H-3->L+1 (11%), H-3->L+2 (14%)
23	59037.36454	169.3842548	-6.1932	0.0071	H-2->L+1 (20%), H-2->L+5 (37%)
24	59350.30766	168.4911232	-0.9004	0.0033	HOMO->L+13 (12%), HOMO->L+15 (10%), HOMO->L+18 (14%)
25	59807.62402	167.2027633	8.9339	0.0183	HOMO->L+15 (35%)
26	60098.79017	166.3927006	-0.2522	0.0099	H-1->L+2 (22%), H-1->L+3 (16%), H-1->L+8 (24%)
27	60214.12745	166.0739834	12.3347	0.0041	H-2->L+4 (11%), H-1->L+4 (27%)
28	60373.82523	165.6346929	-0.7762	0.0157	H-6->LUMO (15%), HOMO->L+15 (14%), HOMO->L+17 (13%)
29	60531.10334	165.2043239	9.1153	0.0143	H-2->L+4 (10%), HOMO->L+17 (15%)

30	60894.85939	164.2174742	-1.9151	0.0022	H-6->LUMO (17%), H-2->L+4 (12%), HOMO->L+17 (11%)
31	61040.03919	163.8268935	0.0648	0.0015	HOMO->L+11 (10%), HOMO->L+20 (10%), HOMO->L+21 (21%)
32	61238.45158	163.2960949	-18.7976	0.0333	H-1->L+3 (15%)
33	61533.6505	162.5127051	-1.8456	0.0021	HOMO->L+6 (13%), HOMO->L+7 (12%), HOMO->L+14 (12%), HOMO->L+19 (17%)
34	61550.58814	162.4679845	-8.5989	0.0179	H-8->LUMO (24%)
35	61891.76066	161.572395	-0.8475	0.0017	H-8->LUMO (10%), H-2->L+3 (10%)
36	62174.05471	160.8387943	4.0567	0.0075	HOMO->L+6 (10%)
37	62251.48394	160.6387409	-5.7522	0.0042	H-2->L+3 (20%)
38	62742.67559	159.3811534	24.6967	0.0057	HOMO->L+20 (13%), HOMO->L+26 (11%)
39	63147.56591	158.3592314	-2.4795	0.0093	H-1->L+5 (11%)
40	63289.51949	158.0040437	-11.7542	0.0114	H-1->L+5 (15%)
41	63669.40663	157.0613035	-5.7599	0.0007	HOMO->L+22 (11%), HOMO->L+23 (11%)
42	64012.19226	156.2202394	-2.0966	0.0071	HOMO->L+24 (10%)
43	64154.14584	155.8745716	-15.5733	0.0073	
44	64188.82768	155.7903511	-18.6523	0.0091	H-2->L+9 (17%)
45	64317.87639	155.4777701	-0.0047	0.0005	H-10->LUMO (17%)
46	64562.26238	154.8892438	-4.8762	0.0024	H-1->L+3 (11%)
47	64672.76033	154.6246047	-2.8157	0.0035	HOMO->L+16 (12%)
48	64863.10718	154.1708443	8.2131	0.0074	H-10->LUMO (12%)
49	64960.70027	153.9392272	-7.2255	0.0135	
50	65047.00159	153.7349879	7.2162	0.0015	

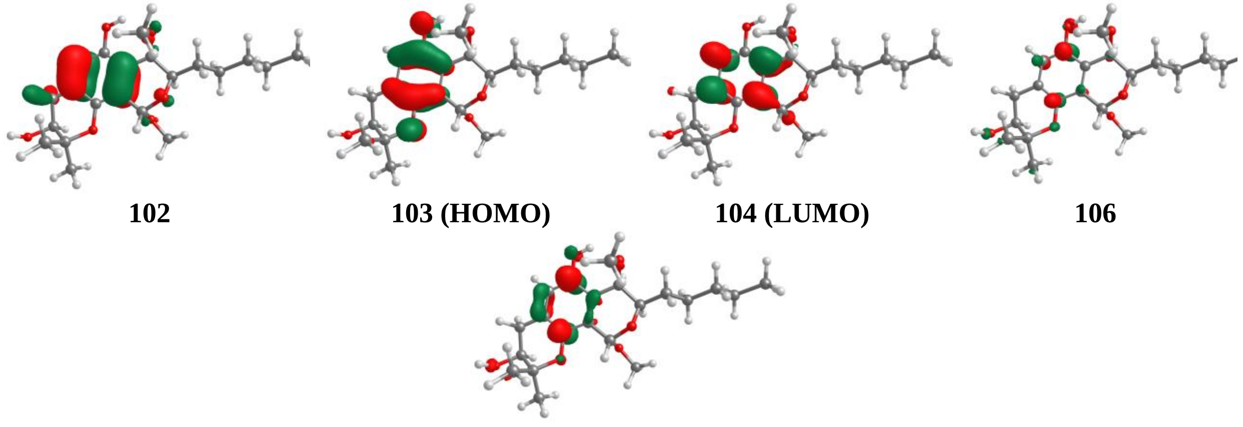

Figure S16. Key molecular orbitals involved in important transitions regarding the ECD spectrum of dominant conformer of **5**.

Table S15. Key transitions and their related rotatory and oscillator strengths of dominant conformer of **6** at the B3LYP/6-31+g(d) level.

HOMO is 107					
**No.**	**Energy (cm-1)**	**Wavelength (nm)**	**R(length)**	**Osc. Strength**	**Major contribs**
1	36093.3107	277.0596492	-7.351	0.1434	HOMO->LUMO (88%)
2	43666.05023	229.0108665	13.5984	0.0582	H-1->LUMO (15%), HOMO->L+1 (29%), HOMO->L+2 (16%), HOMO->L+3 (15%), HOMO->L+4 (12%)

3	45501.76811	219.7716795	3.6619	0.0065	HOMO->L+1 (41%), HOMO->L+2 (11%), HOMO->L+4 (30%)
4	48339.22659	206.871328	-12.0884	0.024	HOMO->L+2 (42%), HOMO->L+4 (10%), HOMO->L+5 (16%)
5	49378.87525	202.5157509	19.3289	0.0678	H-2->LUMO (65%), H-1->LUMO (15%)
6	49907.97496	200.3687789	-47.4991	0.5634	H-2->LUMO (21%), H-1->LUMO (52%), HOMO->L+4 (12%)
7	51029.89217	195.963573	11.8641	0.0301	HOMO->L+3 (24%), HOMO->L+5 (21%)
8	52190.52399	191.6056639	8.6503	0.015	HOMO->L+3 (21%), HOMO->L+6 (24%), HOMO->L+7 (17%), HOMO->L+8 (10%)
9	52776.08251	189.4797705	-6.7375	0.423	H-1->L+1 (19%), H-1->L+2 (12%), H-1->L+3 (17%), H-1->L+4 (16%)
10	54330.31289	184.0593118	-12.4997	0.0489	H-4->LUMO (41%), H-3->LUMO (11%)
11	54437.58463	183.6966145	28.8304	0.0383	H-4->LUMO (13%), HOMO->L+9 (21%)
12	55449.00388	180.3458908	21.2489	0.0512	HOMO->L+7 (11%), HOMO->L+8 (32%), HOMO->L+9 (13%)
13	55686.93744	179.575327	1.809	0.0022	H-1->L+1 (13%), HOMO->L+6 (13%), HOMO->L+7 (21%), HOMO->L+11 (13%)
14	55827.27791	179.1239046	15.3314	0.0095	H-2->L+1 (24%), H-2->L+2 (29%)
15	56064.40491	178.3662917	-11.569	0.0349	H-4->LUMO (10%), H-3->LUMO (58%)
16	56116.83095	178.1996565	11.2282	0.0055	H-1->L+1 (27%), H-1->L+4 (11%)
17	56712.06812	176.3293128	6.343	0.0049	HOMO->L+5 (14%), HOMO->L+10 (25%), HOMO->L+12 (21%)
18	57424.25568	174.14244	6.9337	0.004	HOMO->L+10 (12%), HOMO->L+12 (43%)
19	57931.57842	172.6174269	-3.2483	0.0163	H-6->LUMO (52%)
20	58682.48059	170.4086109	-4.4788	0.0007	H-5->LUMO (15%), H-2->L+1 (14%), H-2->L+4 (21%)
21	58924.44692	169.7088479	-30.7107	0.0201	H-6->LUMO (10%), H-5->LUMO (26%), H-2->L+4 (22%)
22	59062.36773	169.3125485	2.3256	0.0047	HOMO->L+15 (20%)
23	59079.30537	169.2640077	4.5674	0.0152	H-5->LUMO (16%), H-4->L+1 (12%)
24	59634.21482	167.688969	0.3158	0.0006	HOMO->L+7 (17%), HOMO->L+8 (12%), HOMO->L+14 (27%)
25	60095.56395	166.4016334	3.8255	0.0041	H-8->LUMO (22%), H-1->L+2 (16%), H-1->L+5 (14%)
26	60439.96269	165.4534443	-19.3604	0.0057	
27	60619.82433	164.9625368	-4.4013	0.0149	H-2->L+5 (11%)
28	60755.32547	164.5946248	10.7642	0.0043	
29	61065.84893	163.7576514	3.4909	0.0021	H-8->LUMO (21%)
30	61156.98958	163.5136077	-22.1207	0.0109	
31	61403.79523	162.8563831	-1.4047	0.007	H-3->L+1 (19%), H-3->L+3 (12%)
32	61759.48574	161.9184467	-7.4976	0.0134	HOMO->L+13 (11%), HOMO->L+15 (18%)
33	61868.37059	161.6334794	29.6881	0.043	
34	61907.0852	161.5323992	-7.7345	0.0036	
35	62146.63186	160.9097661	-8.1571	0.0084	H-2->L+3 (24%)
36	62507.96825	159.9796039	3.5892	0.0052	HOMO->L+13 (10%), HOMO->L+16 (13%)
37	62755.58046	159.3483787	-11.8954	0.0054	HOMO->L+16 (12%), HOMO->L+20 (12%)
38	62853.17354	159.100956	-2.7735	0.0046	HOMO->L+18 (33%)
39	63167.72977	158.3086813	8.6829	0.0105	HOMO->L+16 (15%)
40	63282.2605	158.022168	-4.918	0.0083	H-2->L+5 (11%)

41	63460.50903	157.5783137	-10.3014	0.0118	HOMO->L+19 (10%), HOMO->L+21 (20%)
42	63578.26597	157.2864539	25.7283	0.024	H-12->LUMO (14%)
43	63753.28829	156.8546544	14.2632	0.0123	H-3->L+2 (15%), H-3->L+3 (15%), H-3->L+4 (19%)
44	64260.61102	155.616323	4.2917	0.0076	H-3->L+5 (11%), H-1->L+7 (13%), H-1->L+9 (11%)
45	64620.3343	154.7500506	1.1	0.0014	H-6->L+4 (11%)
46	64855.84819	154.1880999	-13.2859	0.0049	
47	65097.81452	153.6149881	19.6734	0.0208	
48	65305.90556	153.1255085	-0.3546	0.0059	HOMO->L+11 (11%), HOMO->L+17 (17%)
49	65434.14772	152.8254031	-0.8763	0.0043	H-1->L+9 (10%)
50	65564.80953	152.5208427	0.5528	0.0024	H-5->L+1 (10%), H-5->L+2 (16%)

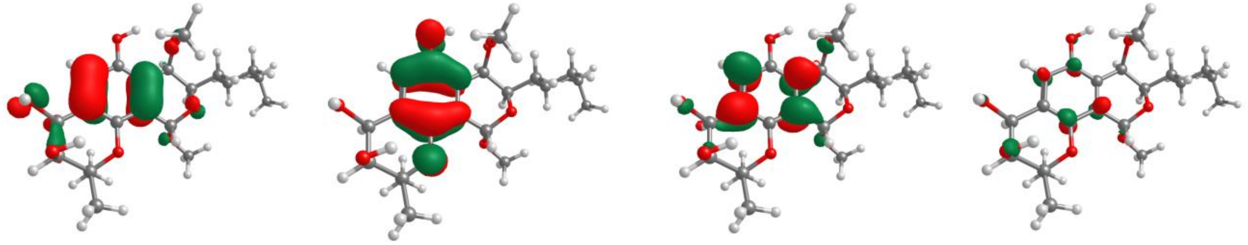

106 **107 (HOMO)** **108 (LUMO)** **109**

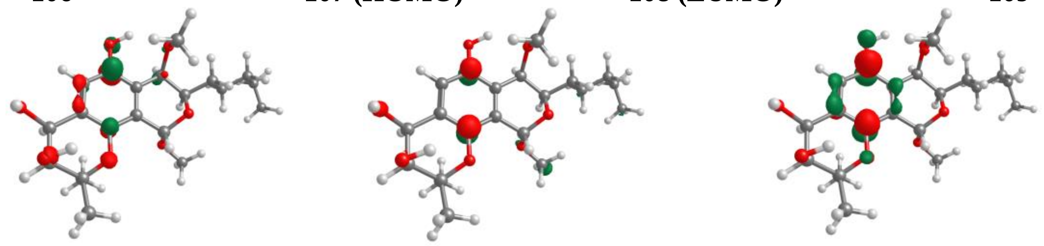

110 **111** **112**

Figure S17. Key molecular orbitals involved in important transitions regarding the ECD spectrum of dominant conformer of **6**.

6. TDP1 inhibition assay

Briefly, TDP1 solution (20 μL/well, 0.02 μL of purified TDP1 (100 nM) in 10 mM Tris-HCl, pH 7.5, 50 mM KCl, 1 mM EDTA, 1 mM DTT) was dispensed into wells of a white 384-well plate (NEST). The tested compound solution in DMSO (5 μL) was pinned into assay plates and incubated at room temperature for 30 min. During this time, the plates were read by a Flash multimode reader (Molecular Devices) at Ex_{485}/Em_{510} nm to identify false-positive compound that had autofluorescence. The linear oligonucleotide substrate 5′-*FAM*-AGGATCTAAAAGACTT-*BHQ*-3′ (25 μL, 35 nM) was dispensed into the wells to start the reaction. The whole plate was immediately read five times using a kinetic read on the Flash

multimode reader (Molecular Devices) (Ex_{485}/Em_{510} nm). TDP1 percentage inhibition of the tested compound was calculated by comparing the rate of increase in fluorescence throughout time for the compound-treated wells to that of DMSO control wells.

7. Molecular Modelling

The X-ray crystal structure of TDP1-ligand complex (PDB: 6DJD) was obtained and cleaned by removing one of the monomers and waters, and inspected for errors. The missing hydrogens were added. The ligand centroid coordination was built as the binding pocket for molecular modeling. Inhibitors were constructed using ChemDraw and saved in mol2 file formats, which were optimized using Discovery Studio software. Every inhibitor was docked 10 times, starting each time from different orientations and the default automatic genetic algorithm parameter settings was used. All torsion angles of the inhibitor were allowed to rotate freely and the results were scored and ranked by using GOLD. The selected top ligand-binding pose was merged into the prepared protein. The calculation was terminated when the gradient reached a value of 0.05 kcal/mol·Å.

Spectral Information

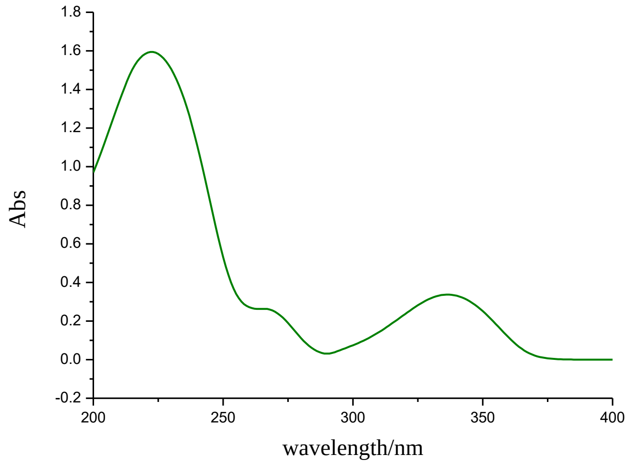

Figure S18. UV spectrum of **1** in MeOH.

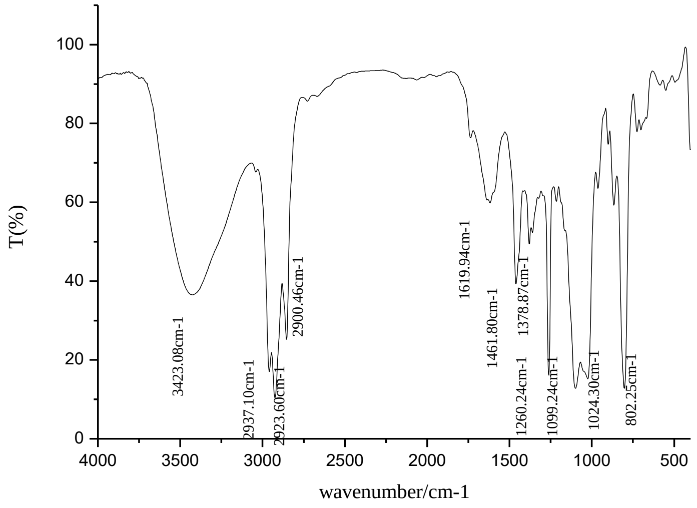

Figure S19. IR spectrum of **1** (KBr disc).

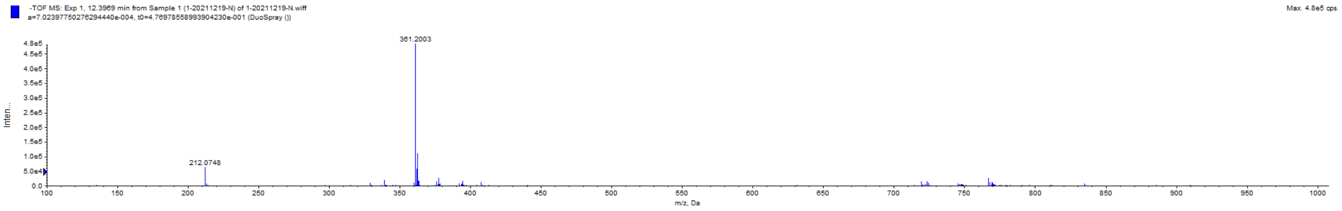

Figure S20. HR-ESI-MS of **1**.

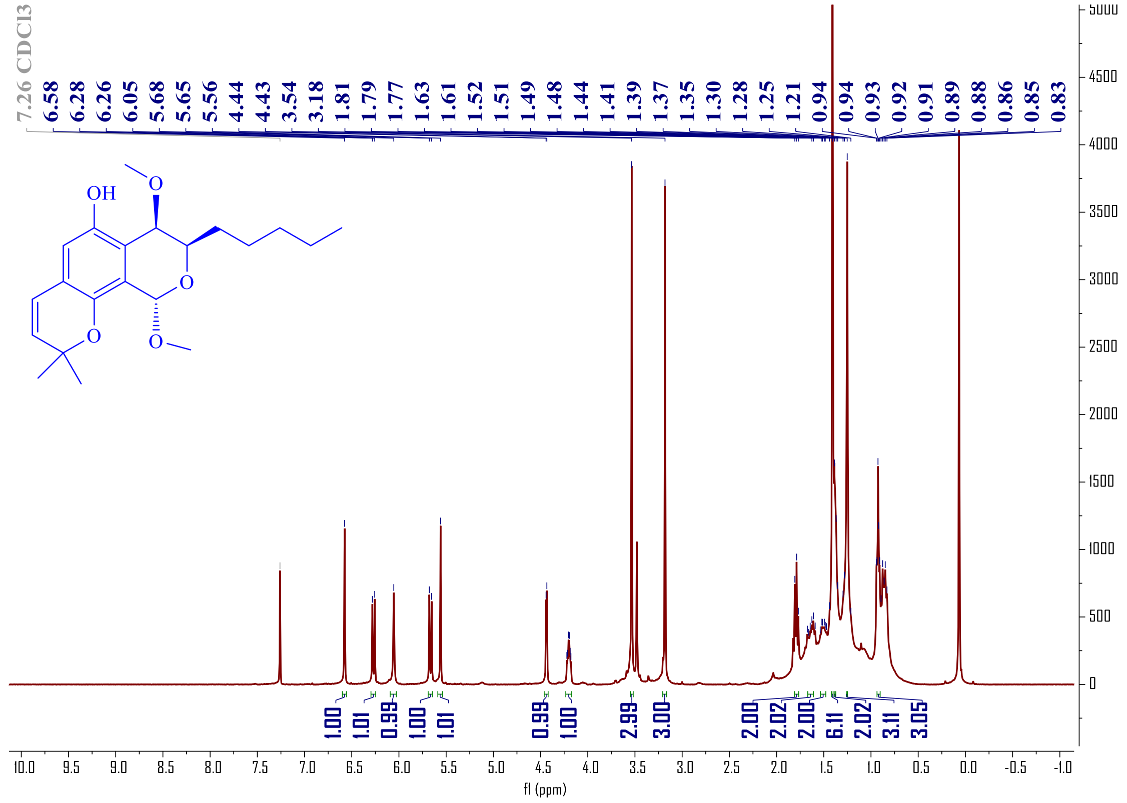

Figure S21. ^{1}H NMR spectrum of **1** in $CDCl_3$.

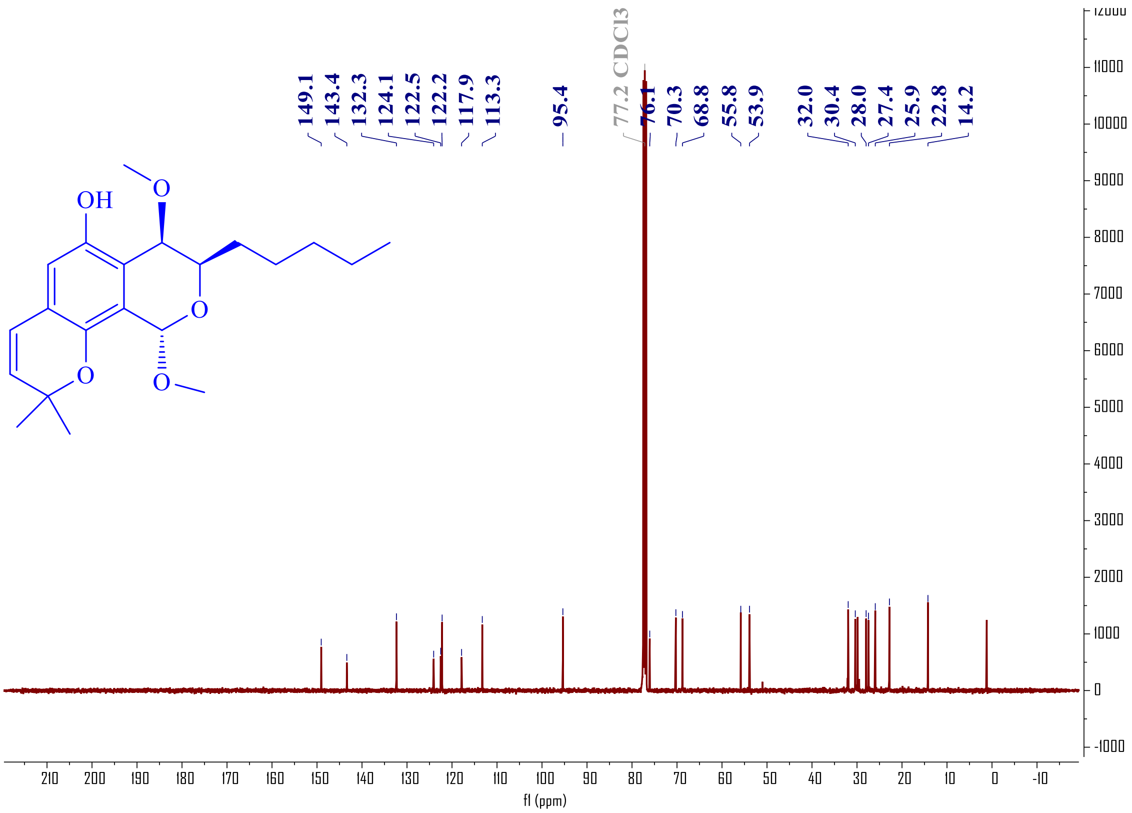

Figure S22. ^{13}C NMR spectrum of **1** in $CDCl_3$.

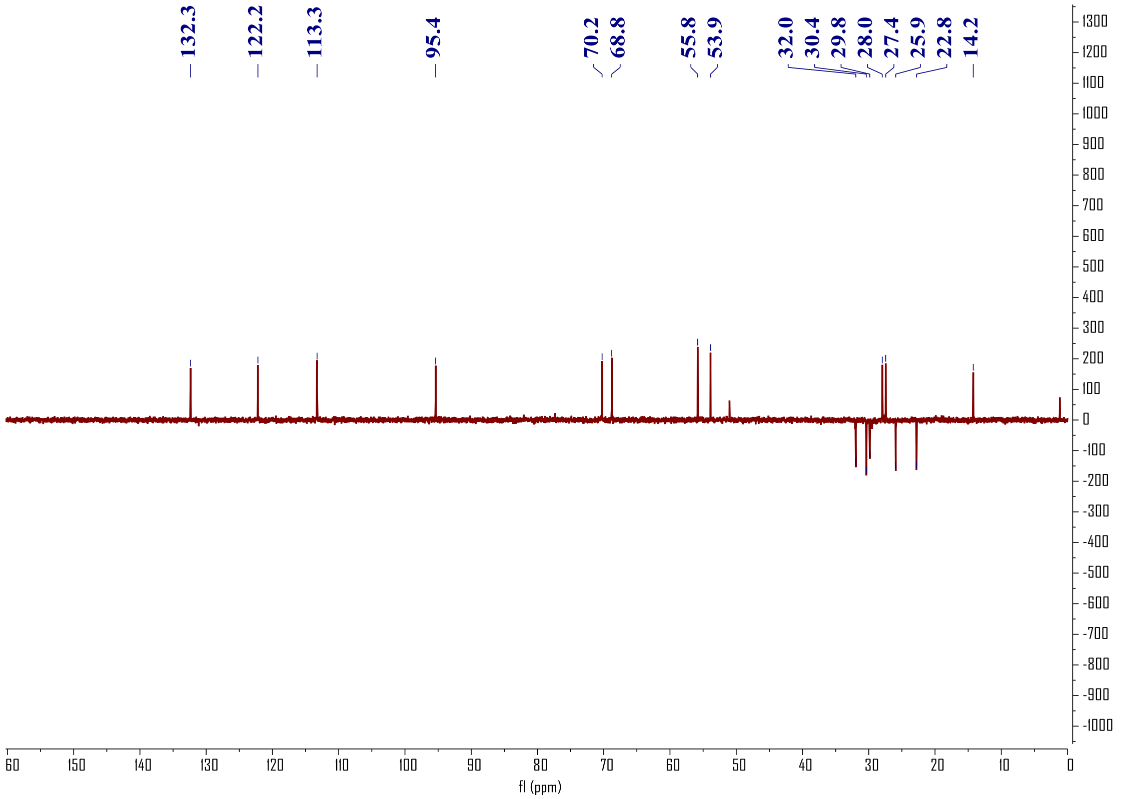

Figure S23. DEPT 135 spectrum of **1** in $CDCl_3$.

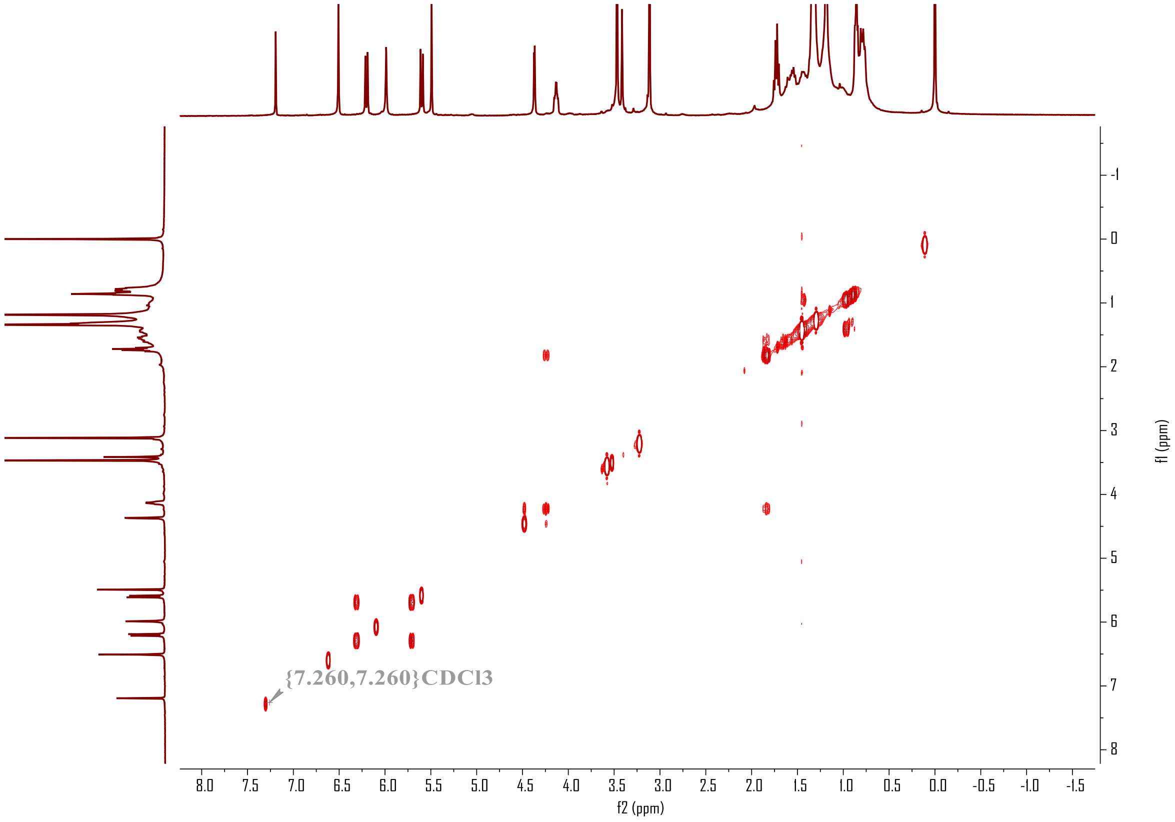

Figure S24. ^1H—^1H COSY spectrum of **1** in $CDCl_3$.

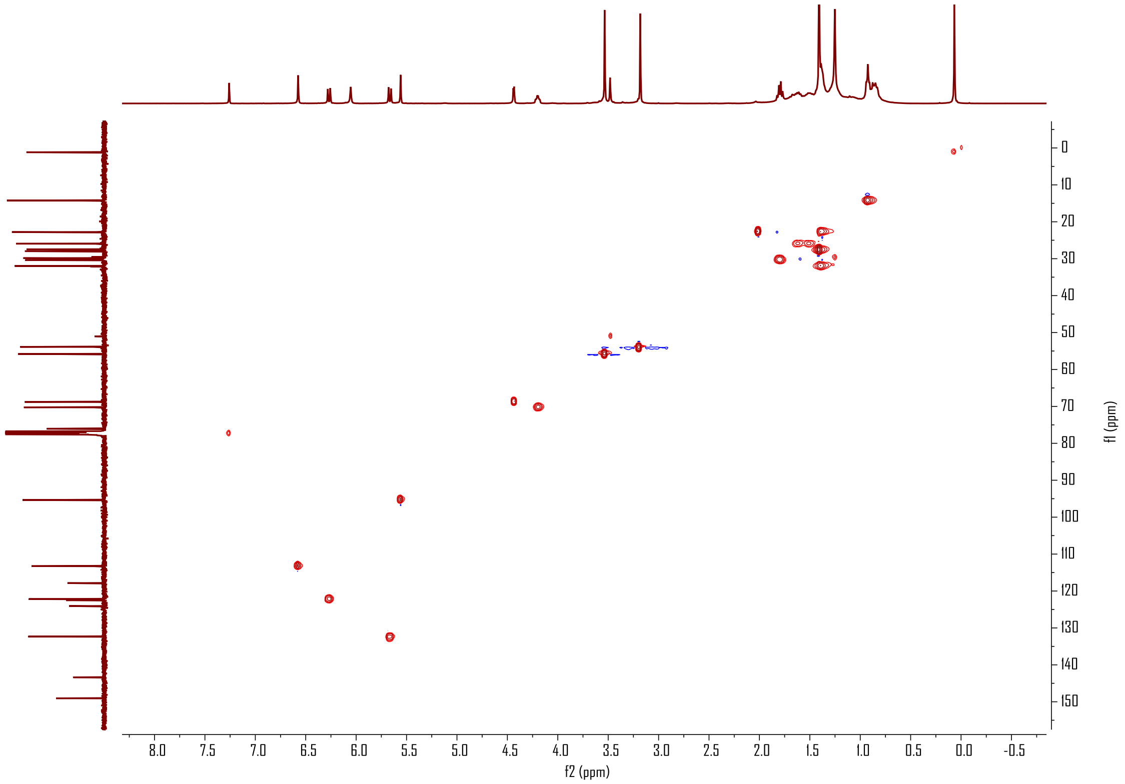

Figure S25. HSQC spectrum of **1** in $CDCl_3$.

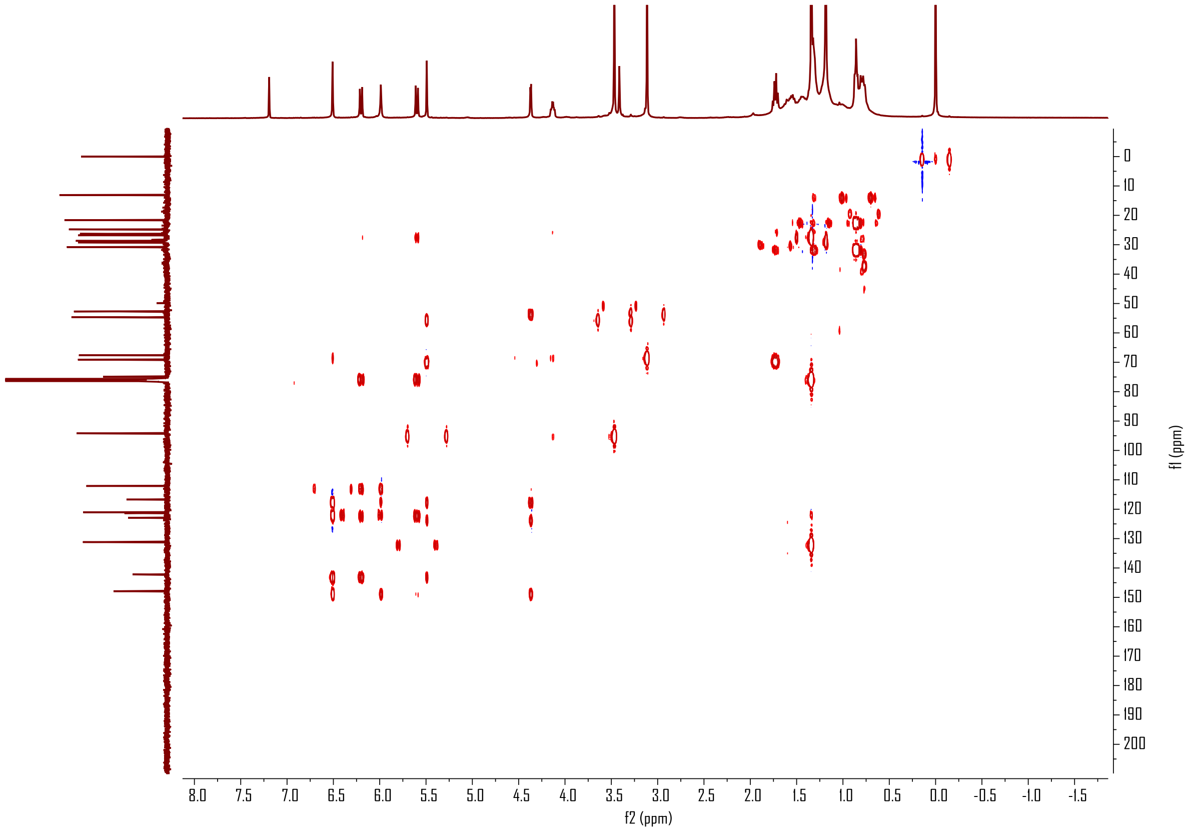

Figure S26. HMBC spectrum of **1** in $CDCl_3$.

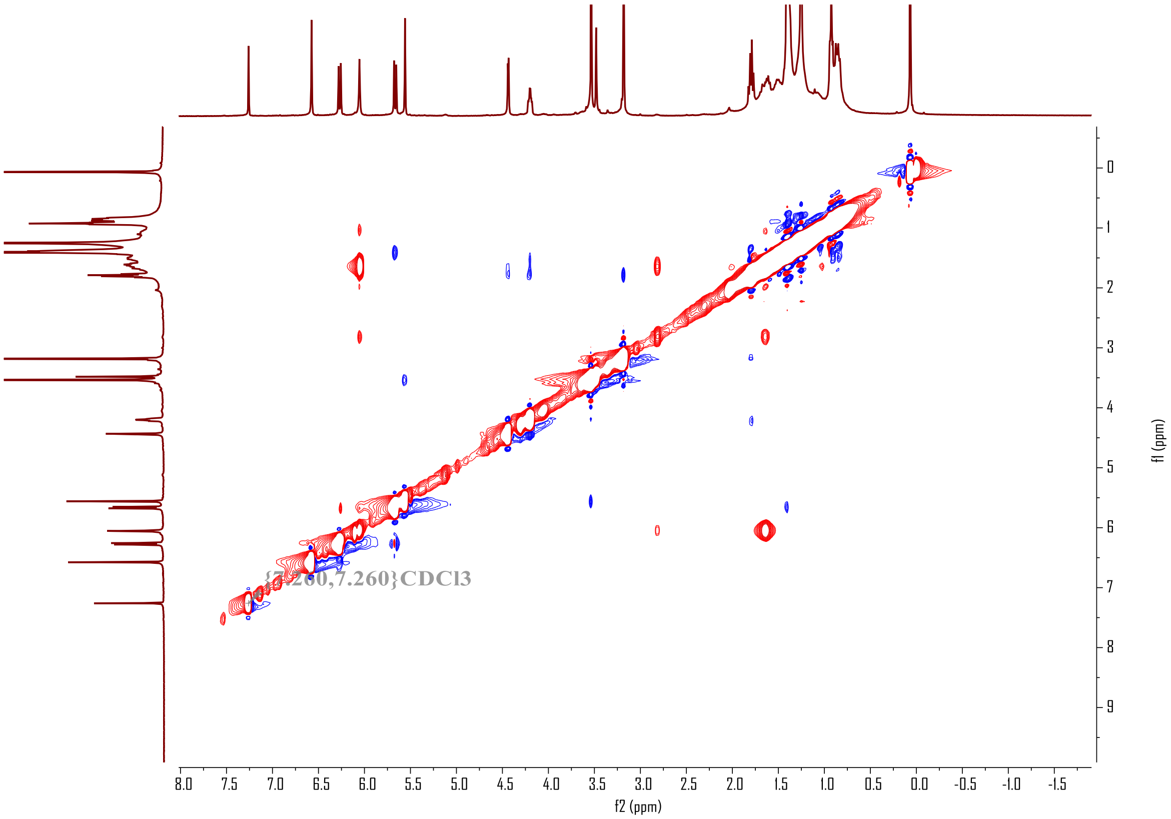

Figure S27. NOESY spectrum of **1** in $CDCl_3$.

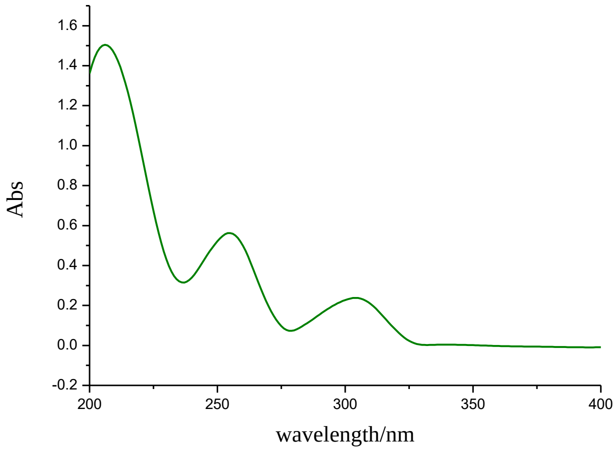

Figure S28. UV spectrum of **3** in MeOH.

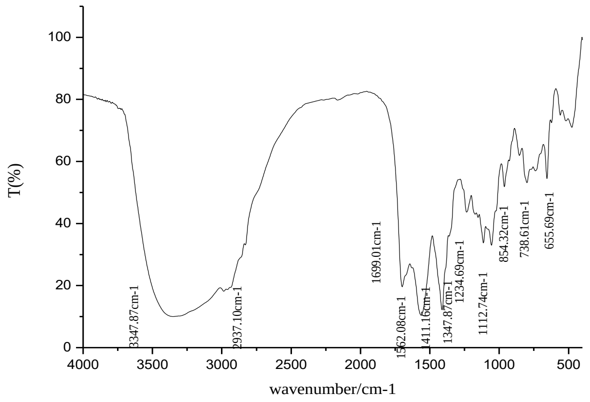

Figure S29. IR spectrum of **3** (KBr disc).

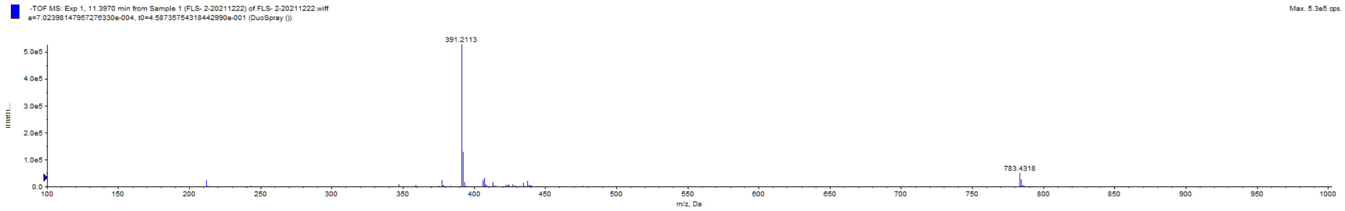

Figure S30. HR-ESI-MS of **3**.

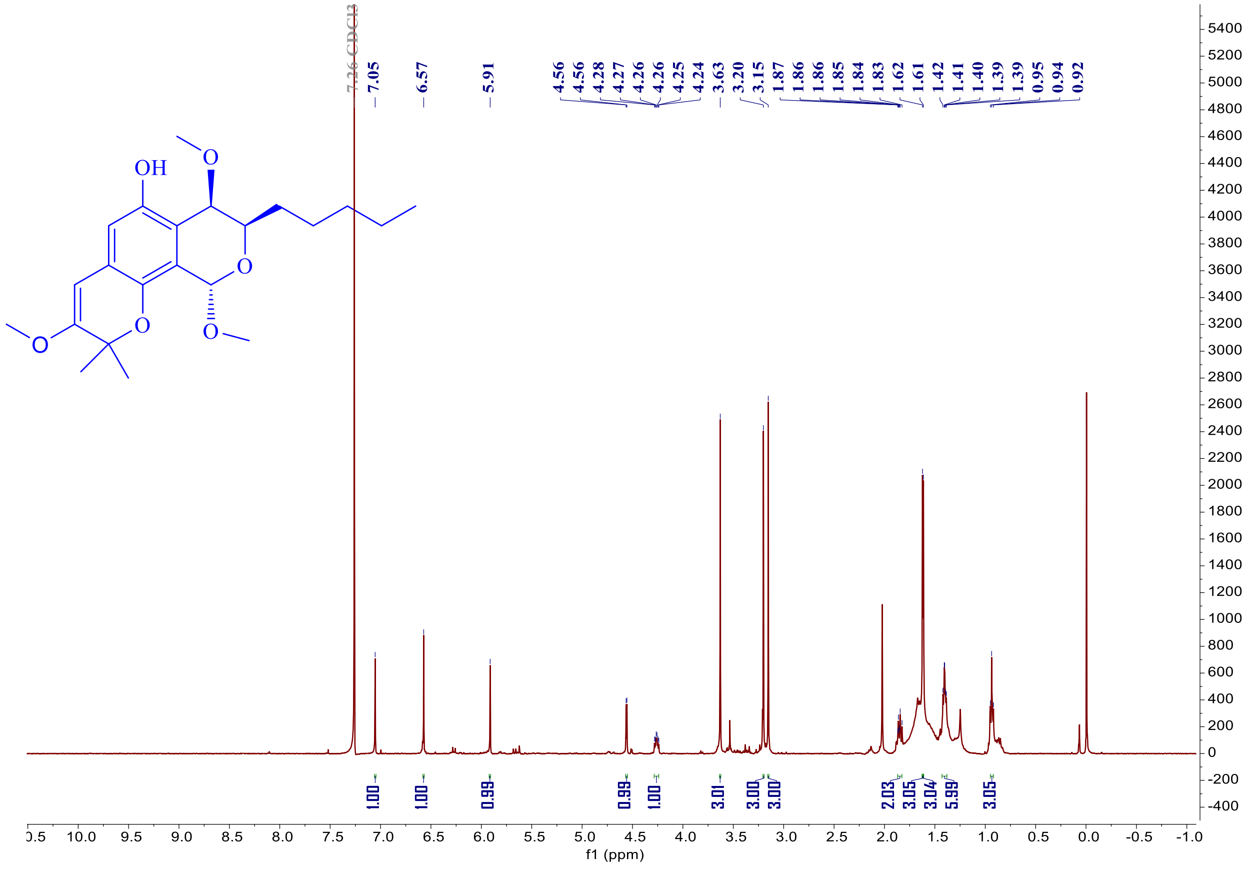

Figure S31. ^{1}H NMR spectrum of **3** in $CDCl_3$.

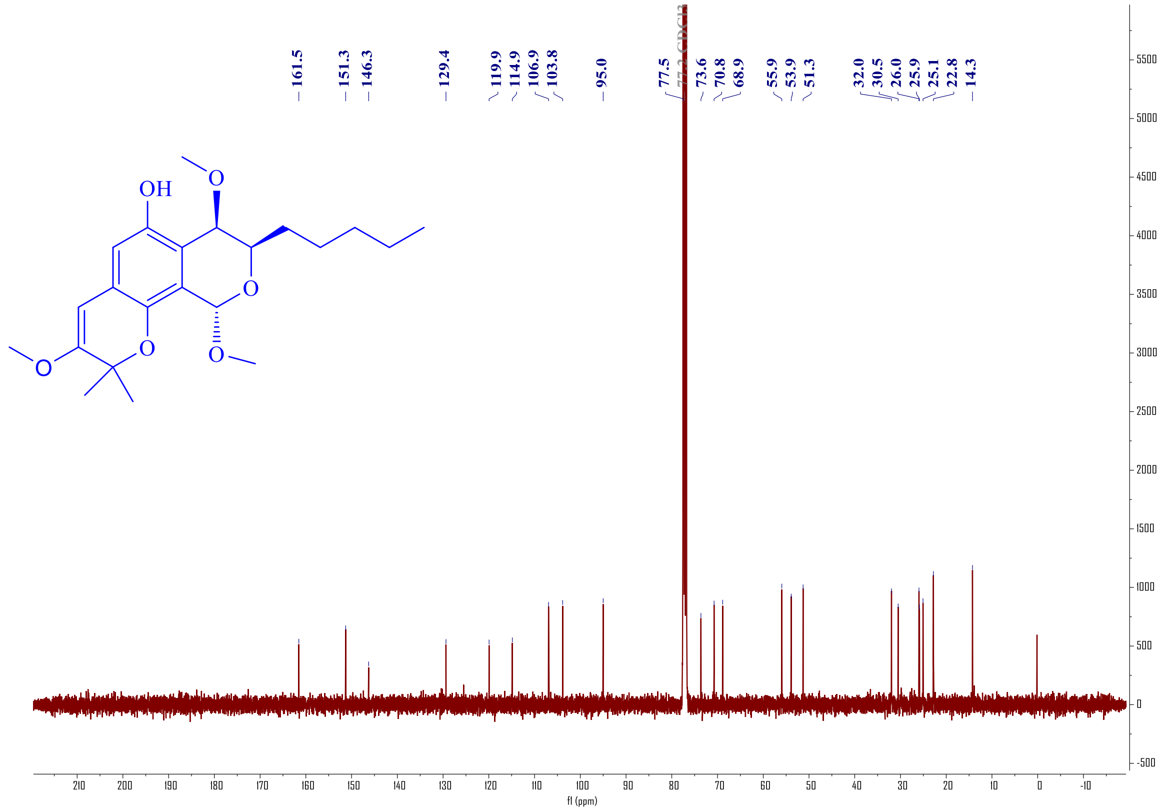

Figure S32. ^{13}C NMR spectrum of **3** in $CDCl_3$.

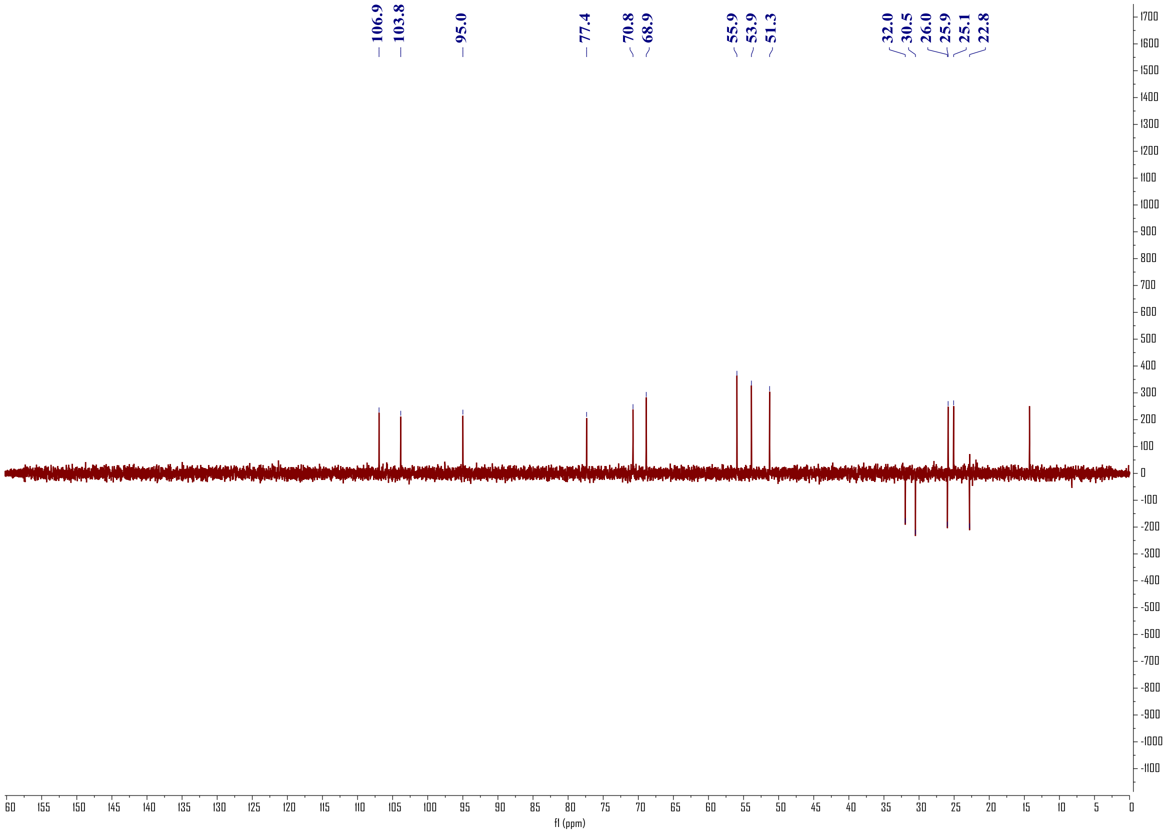

Figure S33. DEPT 135 spectrum of **3** in $CDCl_3$.

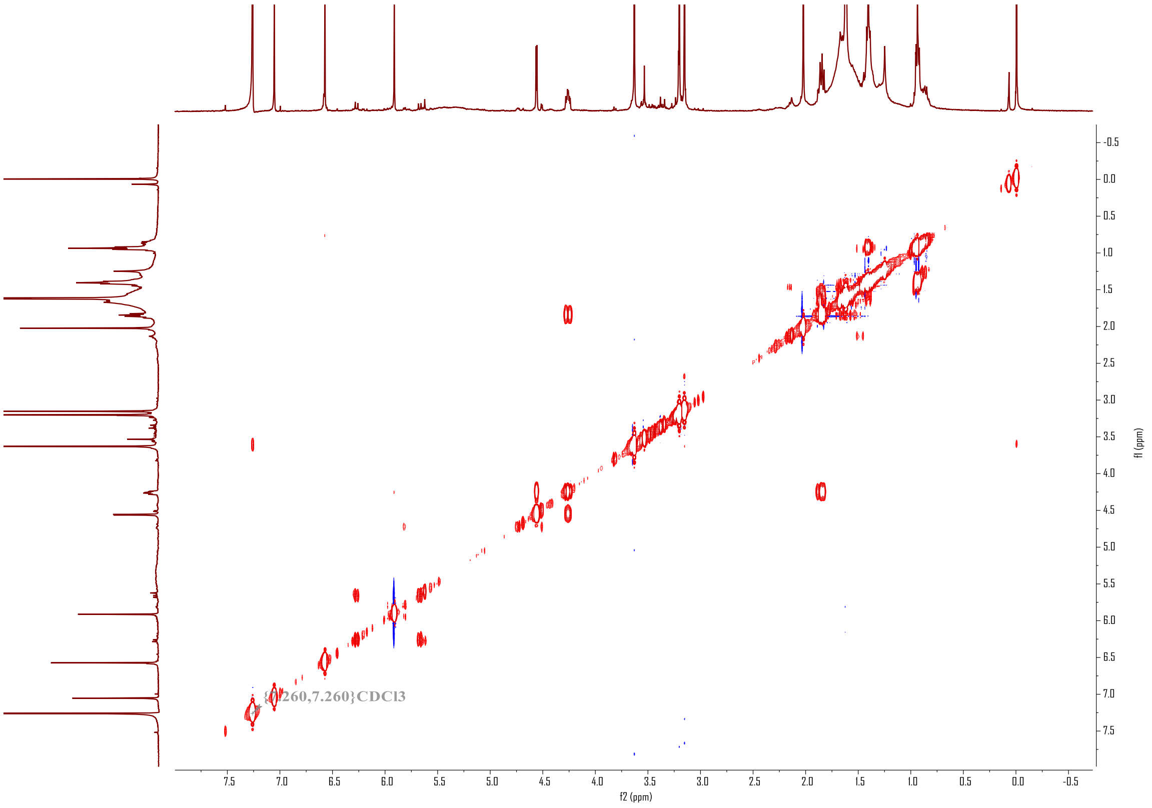

Figure S34. $^{1}H—^{1}H$ COSY spectrum of **3** in $CDCl_3$.

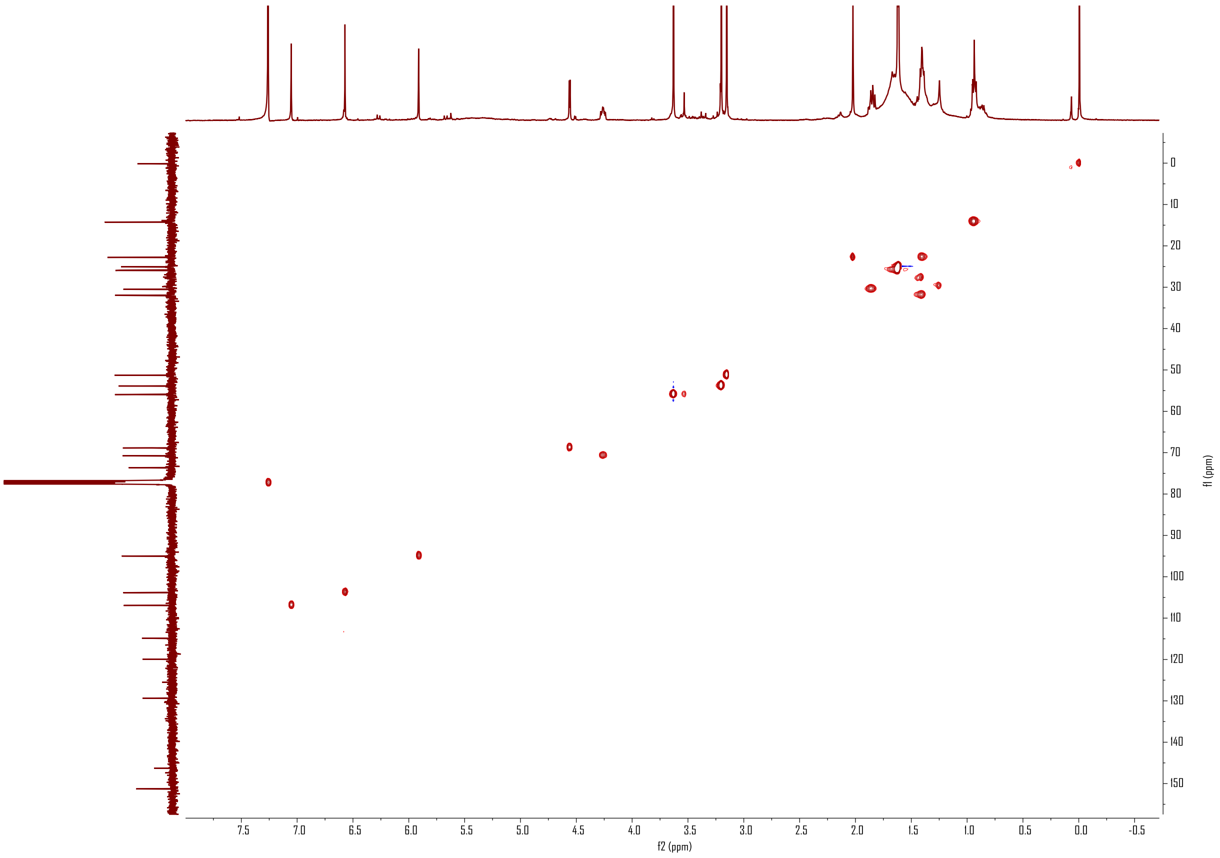

Figure S35. HSQC spectrum of **3** in $CDCl_3$.

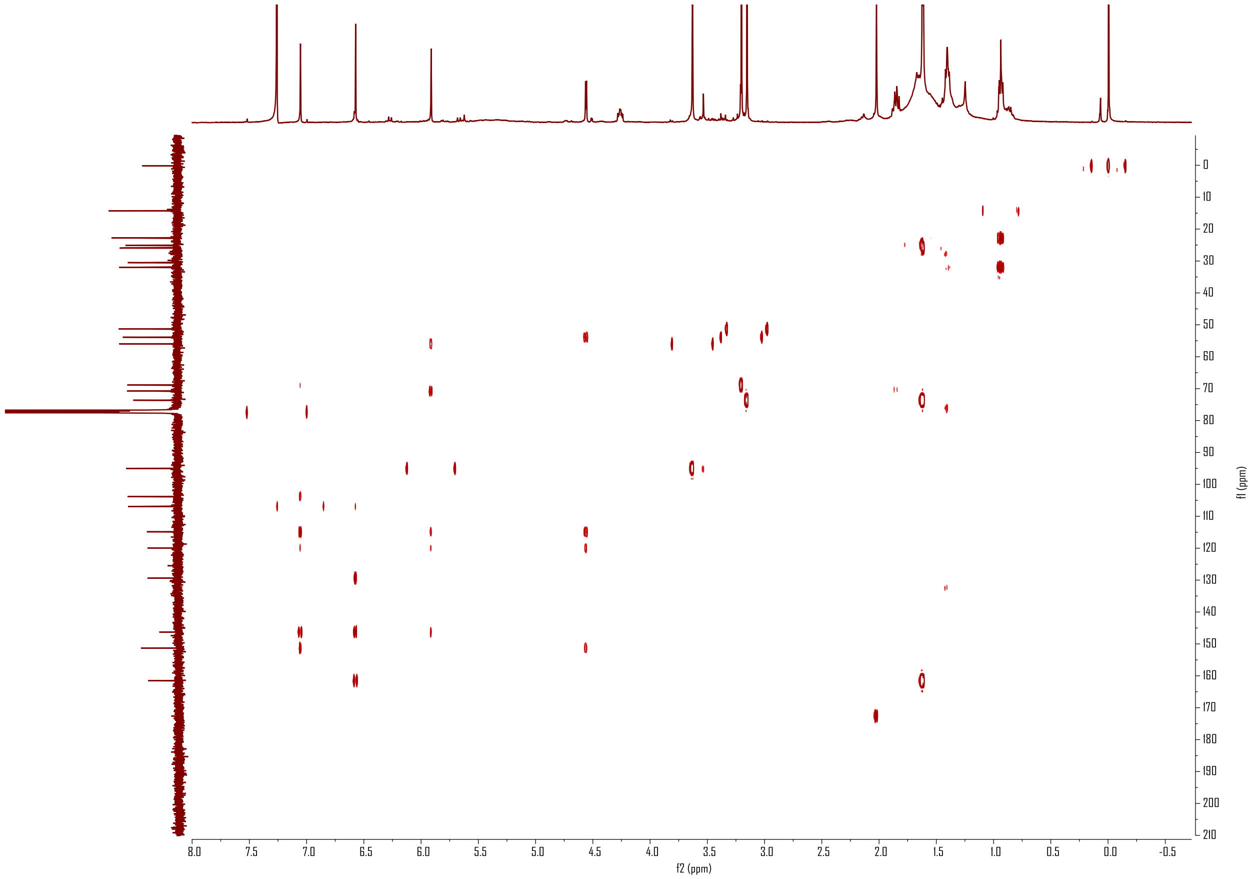

Figure S36. HMBC spectrum of **3** in $CDCl_3$.

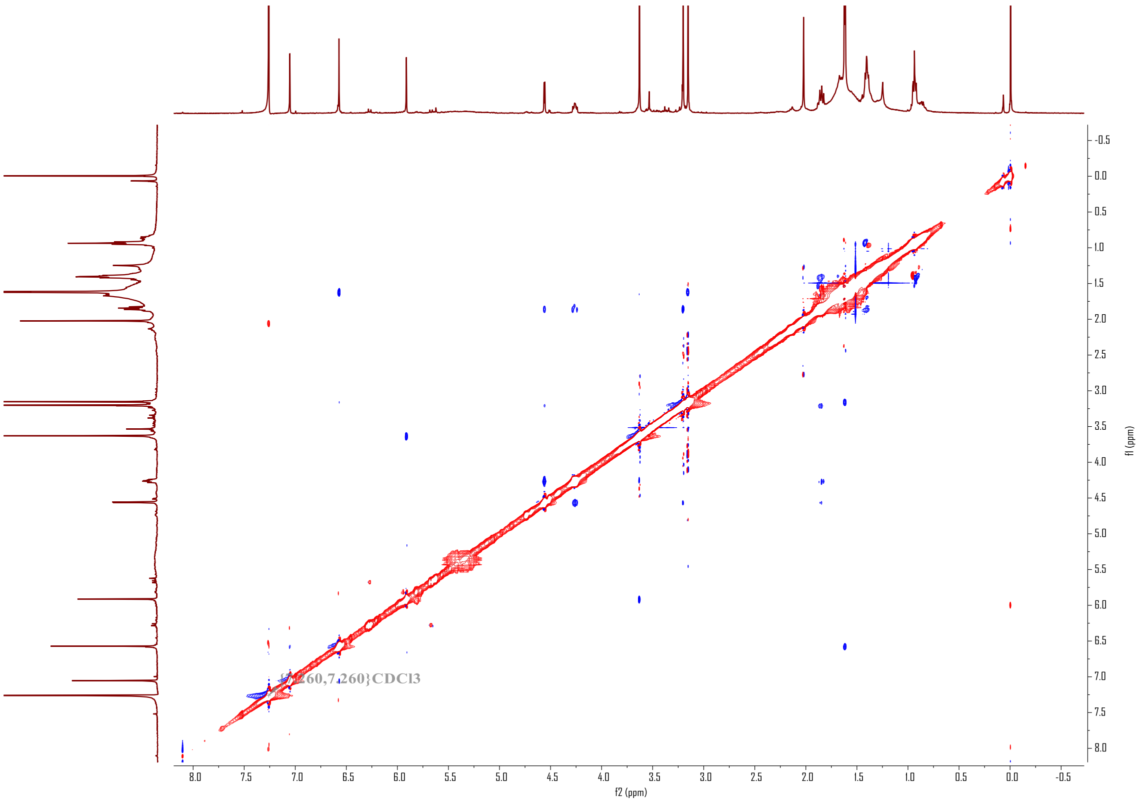

Figure S37. NOESY spectrum of **3** in $CDCl_3$.

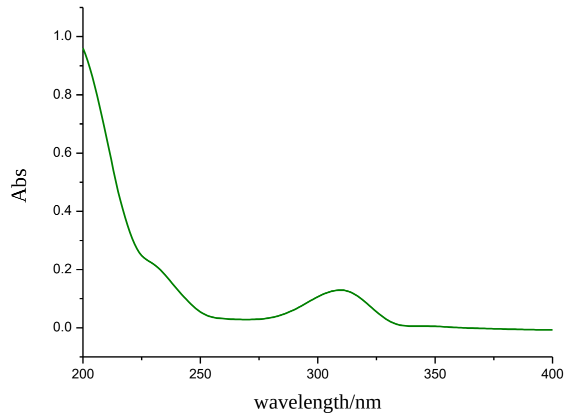

Figure S38. UV spectrum of **4** in MeOH.

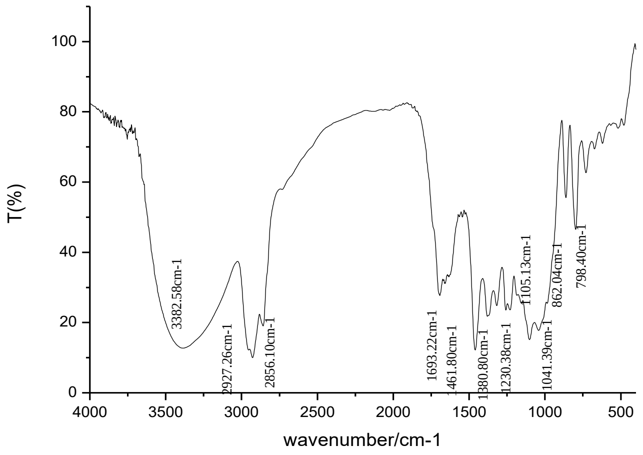

Figure S39. IR spectrum of **4** (KBr disc).

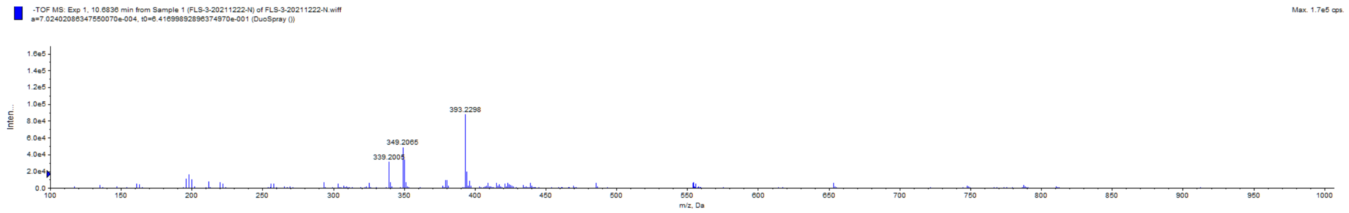

Figure S40. HR-ESI-MS of **4**.

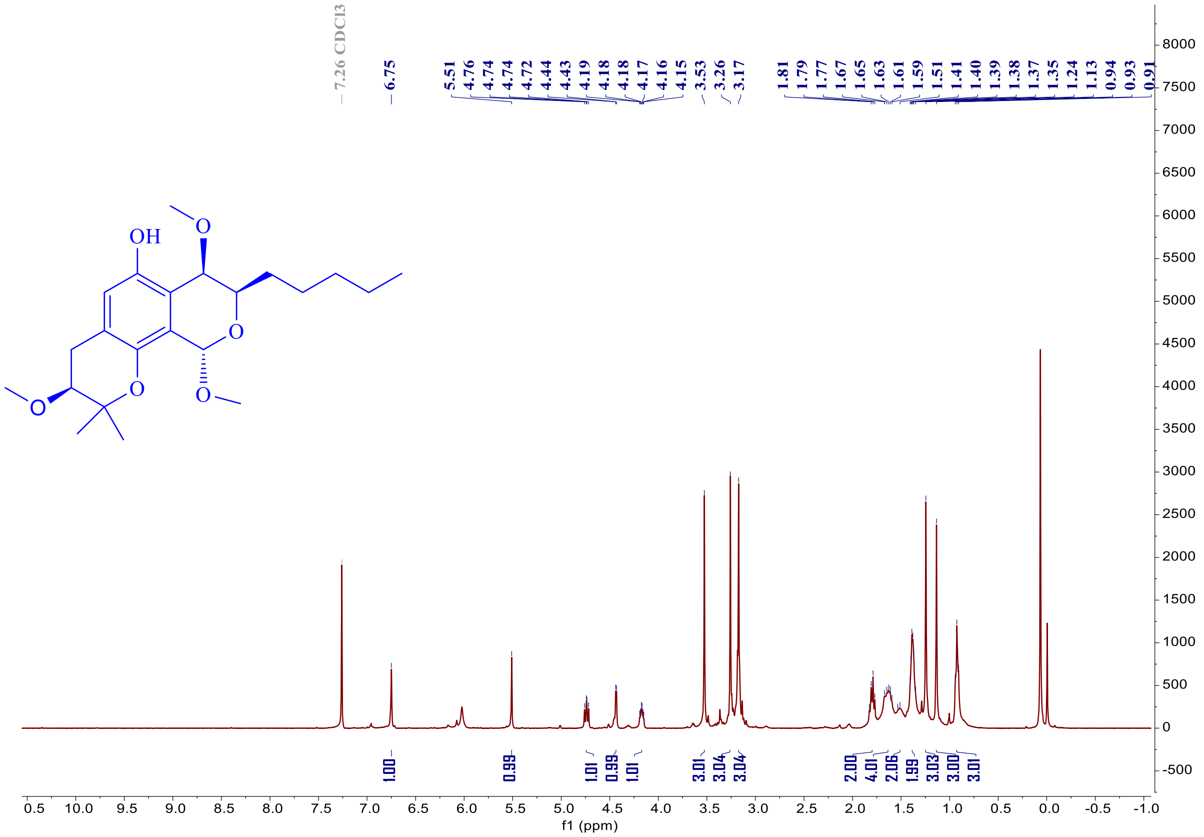

Figure S41. ^{1}H NMR spectrum of **4** in $CDCl_3$.

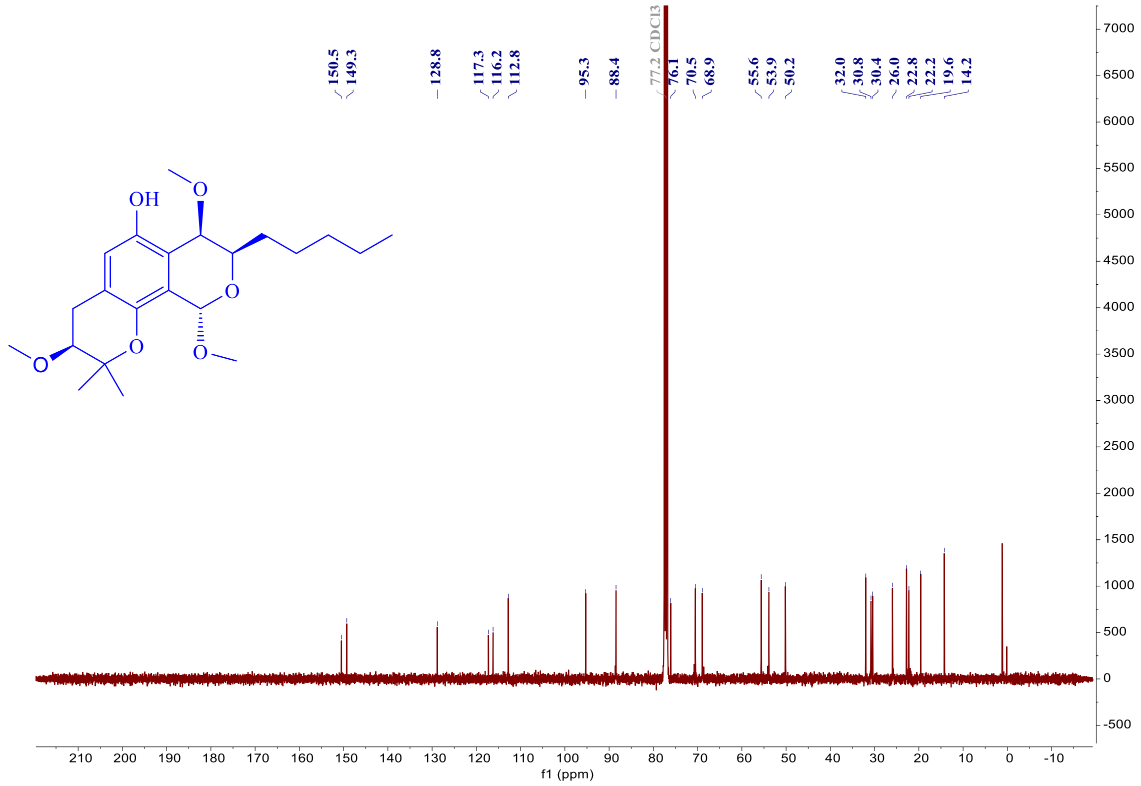

Figure S42. ^{13}C NMR spectrum of **4** in $CDCl_3$

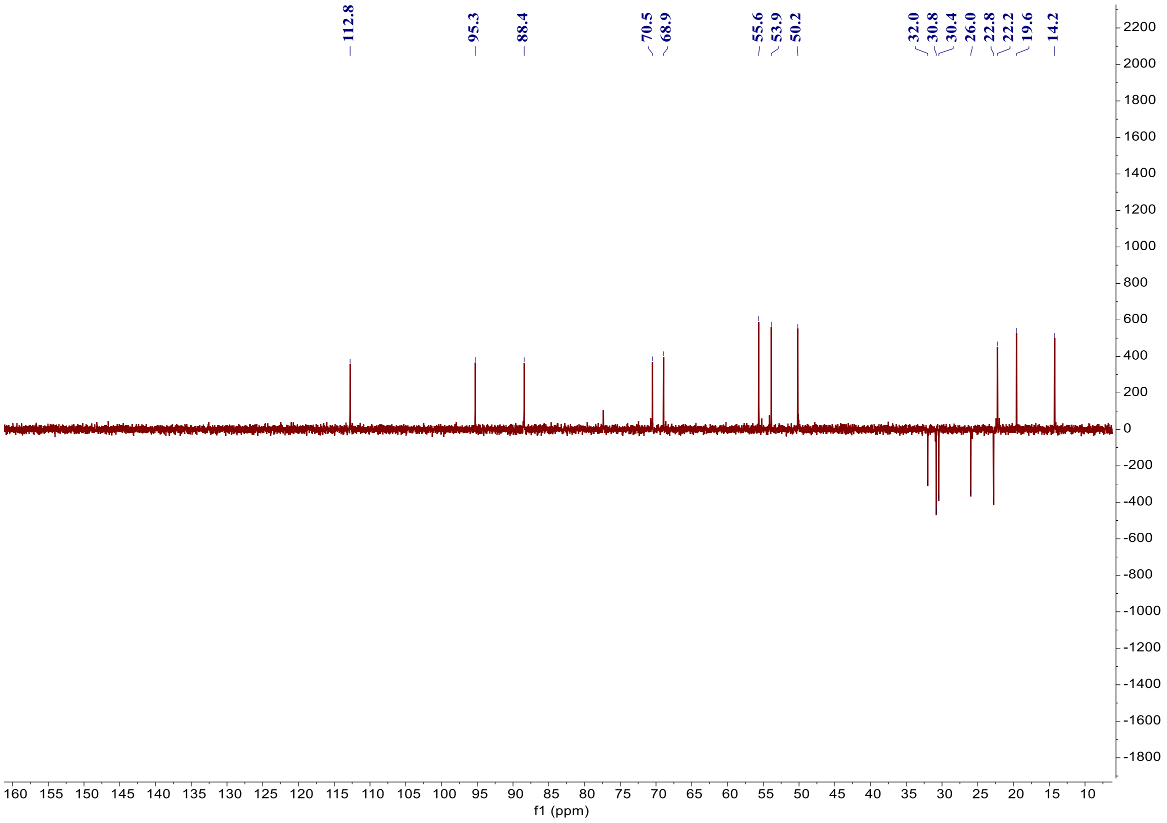

Figure S43. DEPT 135 spectrum of **4** in $CDCl_3$.

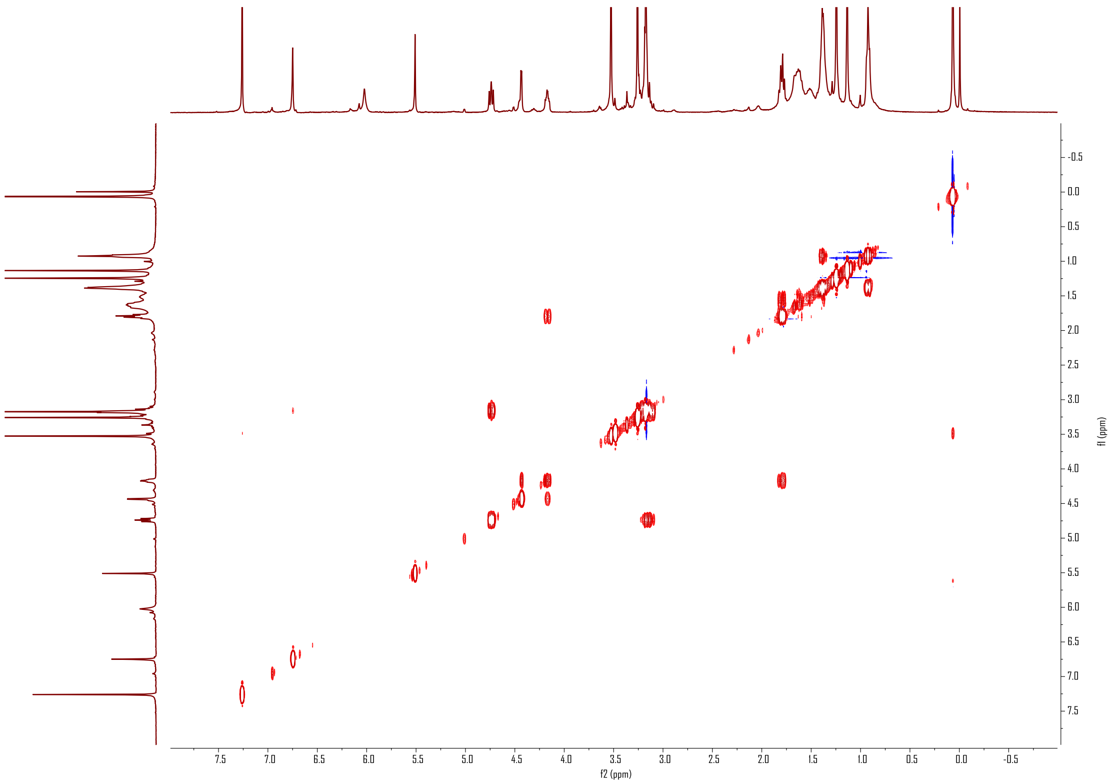

Figure S44. ^1H—^1H COSY spectrum of **4** in $CDCl_3$.

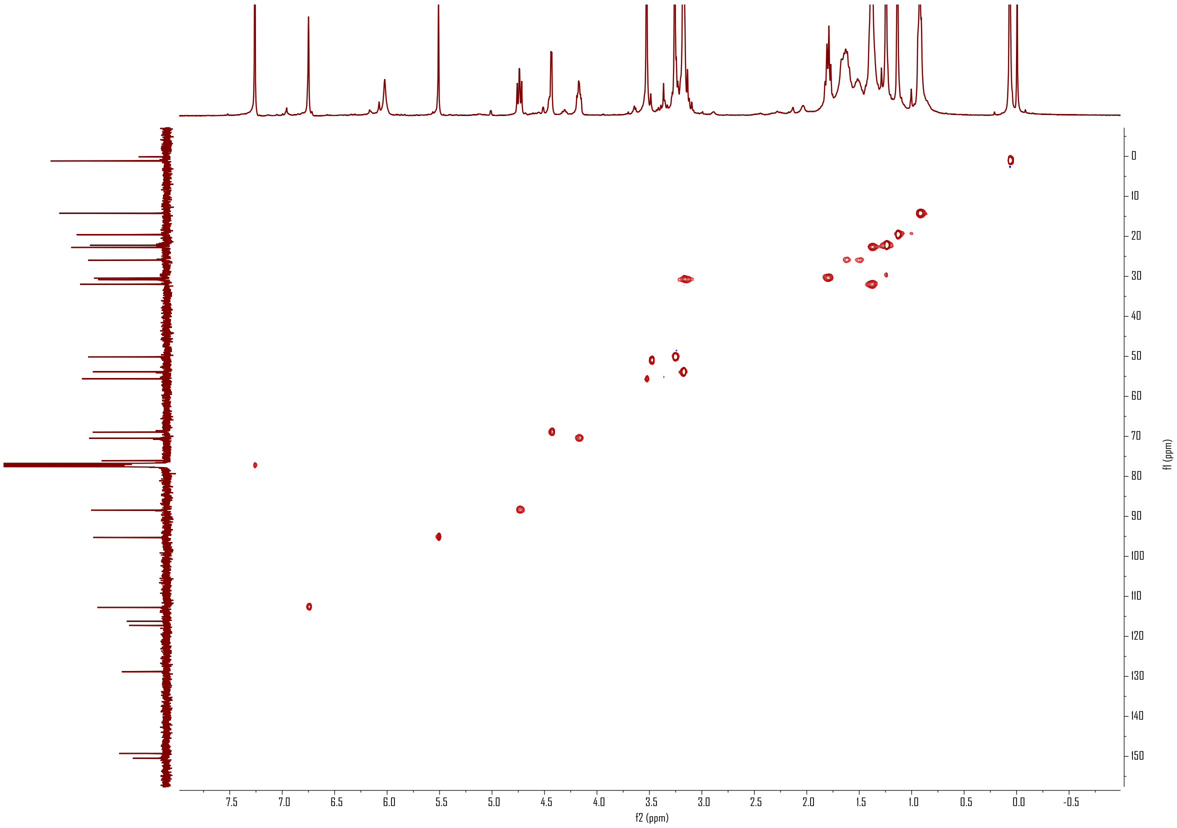

Figure S45. HSQC spectrum of **4** in $CDCl_3$.

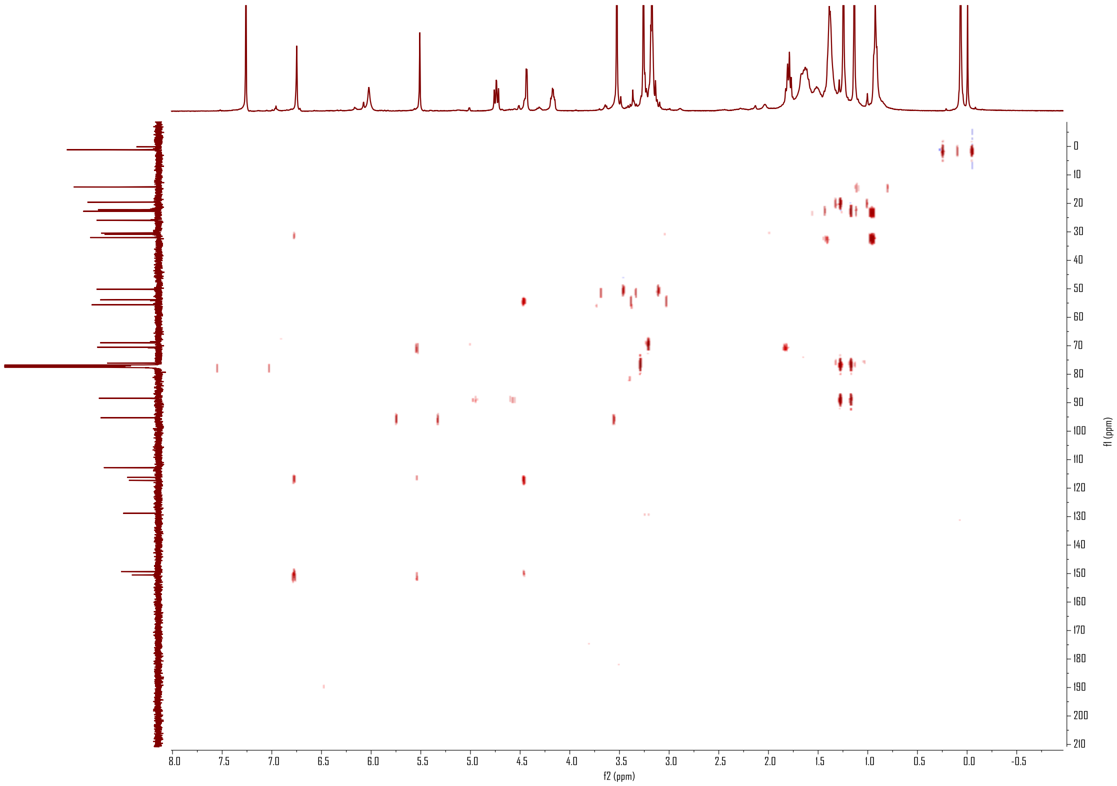

Figure S46. HMBC spectrum of **4** in $CDCl_3$.

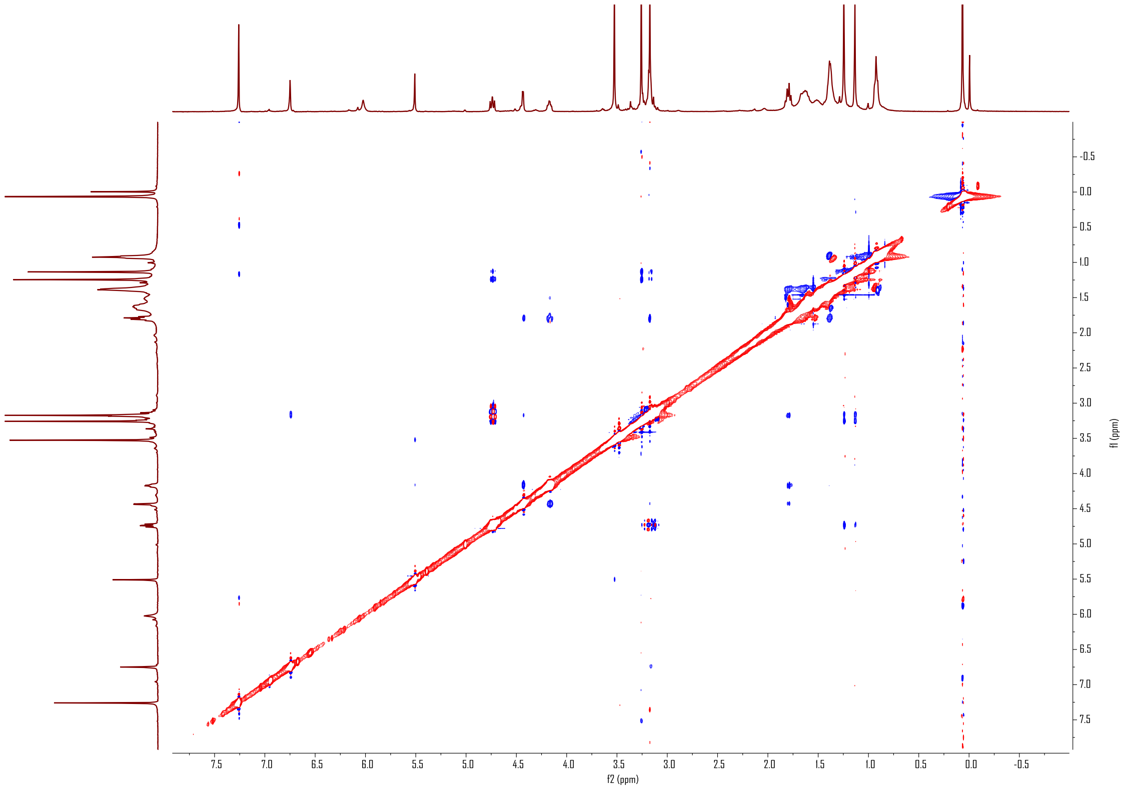

Figure S47. NOESY spectrum of **4** in $CDCl_3$.

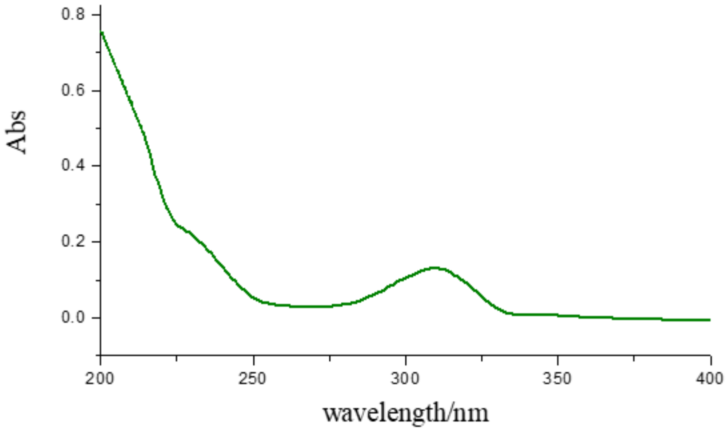

Figure S48. UV spectrum of **5** in MeOH.

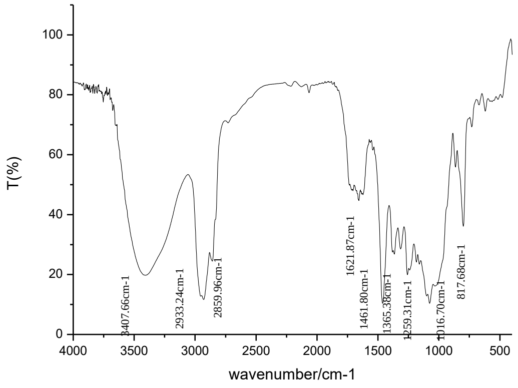

Figure S49. IR spectrum of **5** (KBr disc).

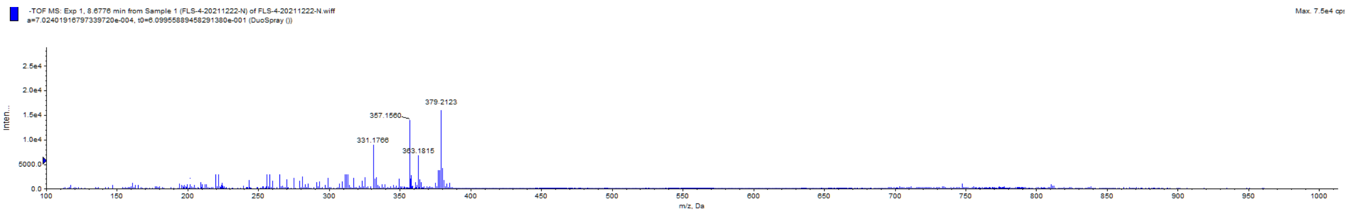

Figure S50. HR-ESI-MS of **5**.

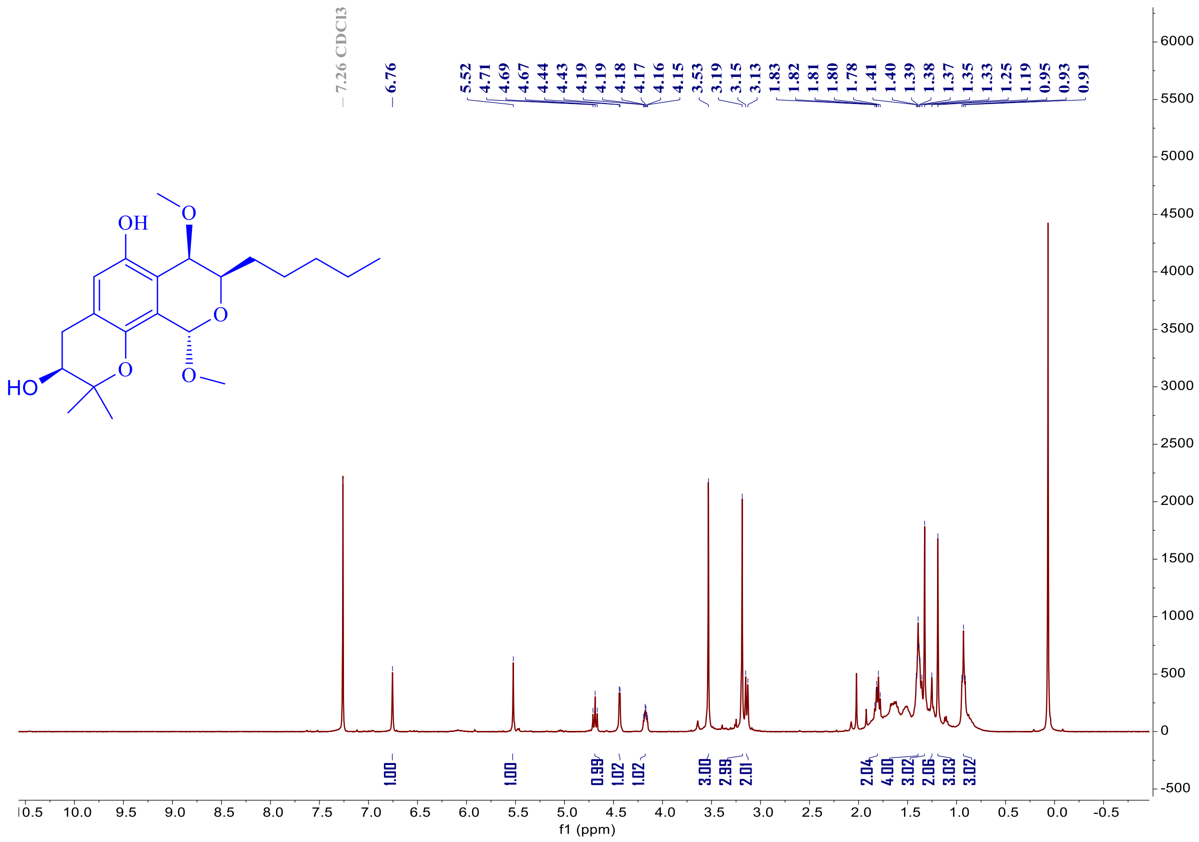

Figure S51. ^{1}H NMR spectrum of **5** in $CDCl_3$.

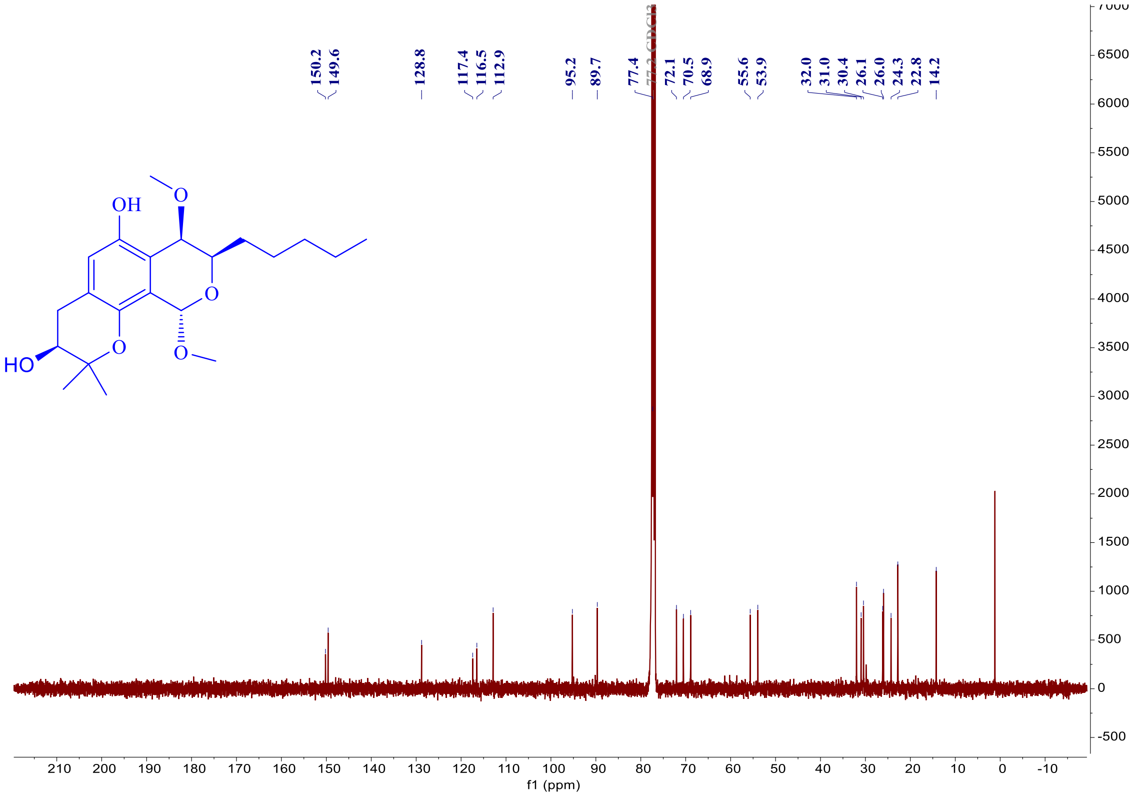

Figure S52. ^{13}C NMR spectrum of **5** in $CDCl_3$.

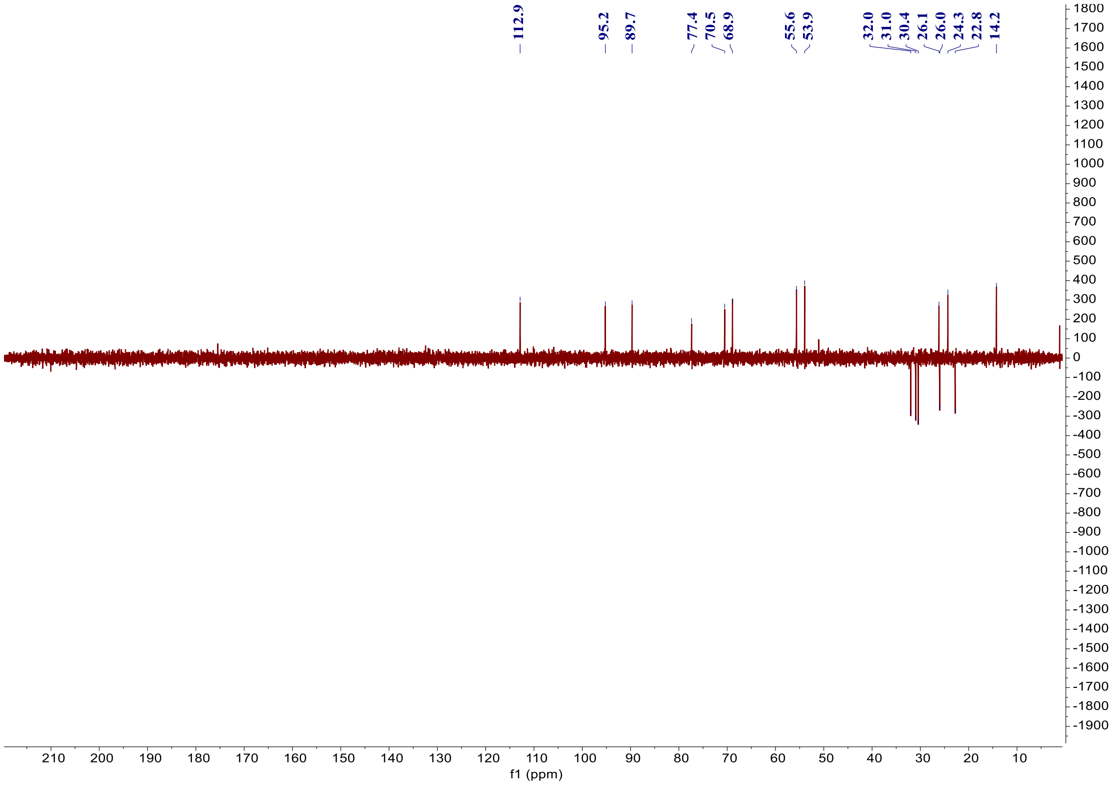

Figure S53. DEPT 135 spectrum of **5** in $CDCl_3$.

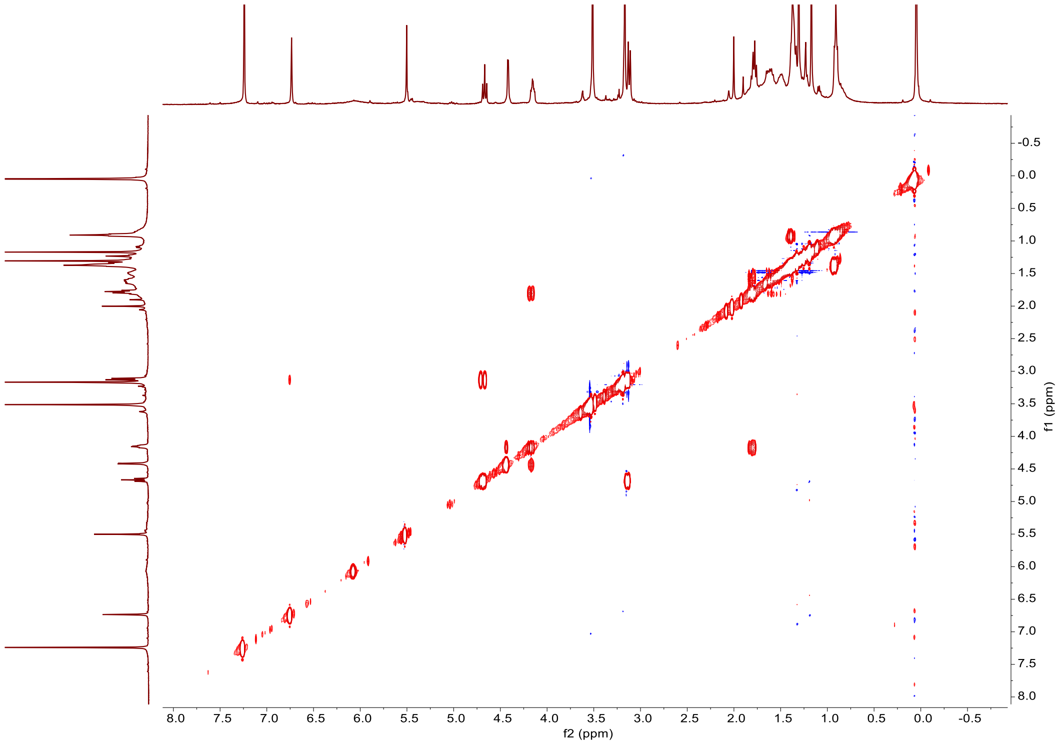

Figure S54. ^{1}H—^{1}H COSY spectrum of **5** in $CDCl_3$.

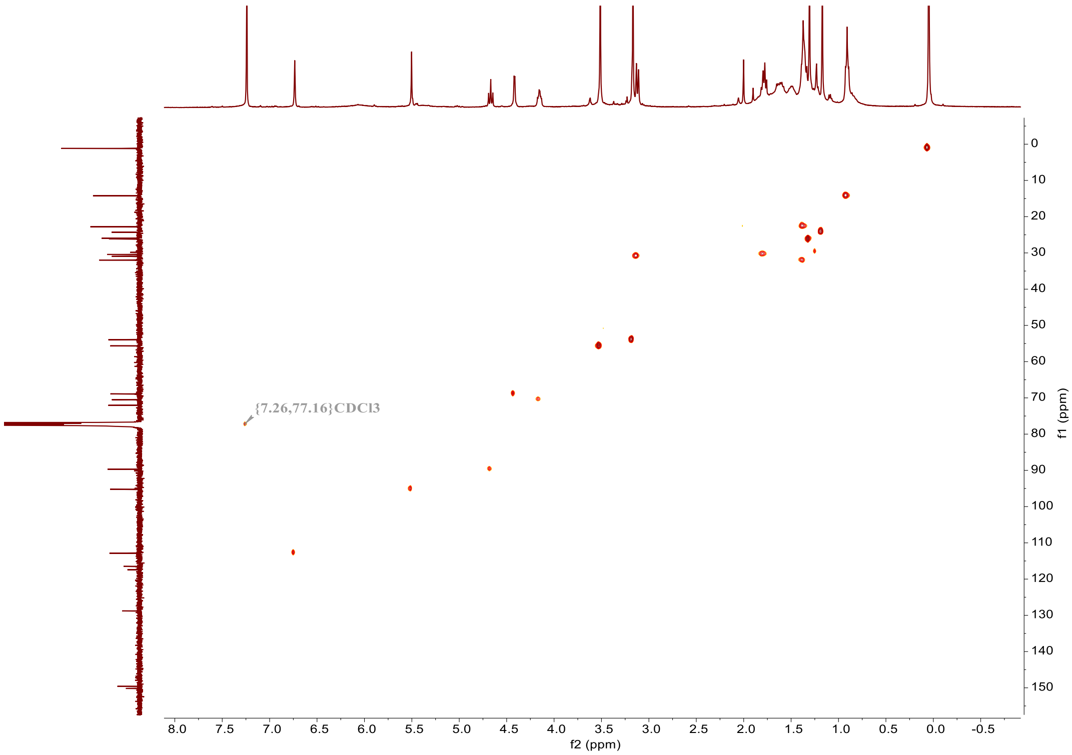

Figure S55. HSQC spectrum of **5** in $CDCl_3$.

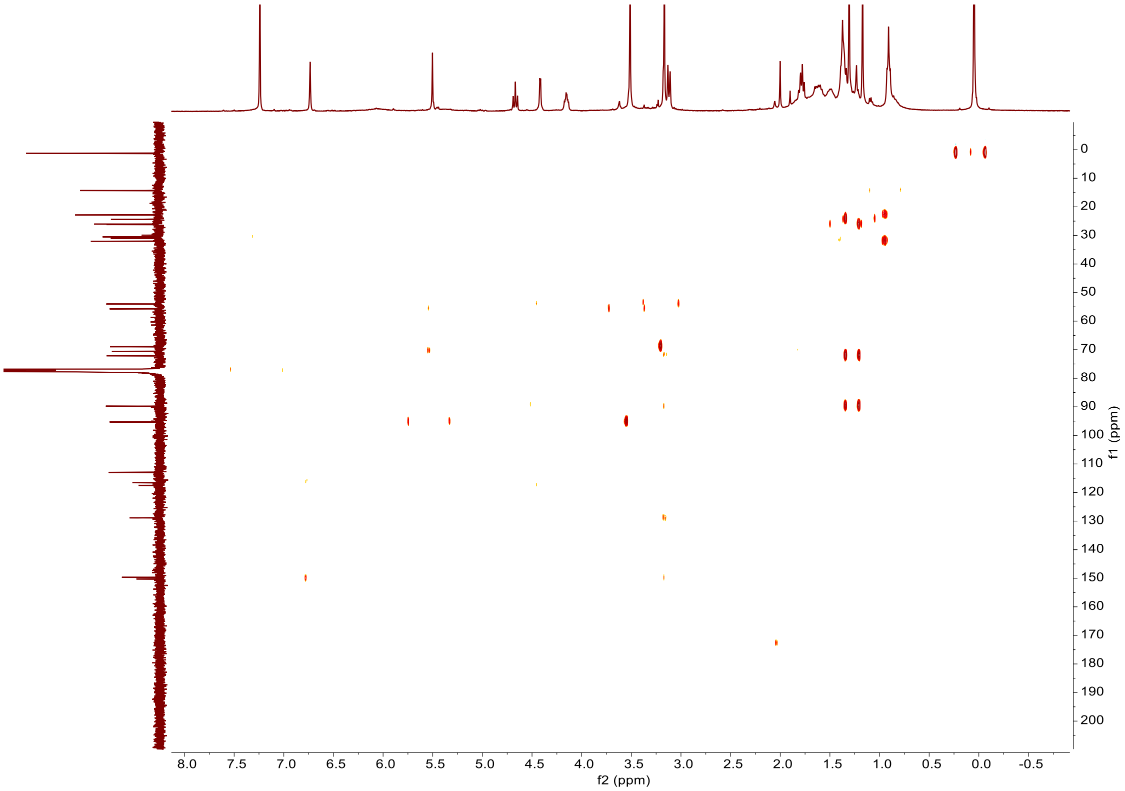

Figure S56. HMBC spectrum of **5** in $CDCl_3$.

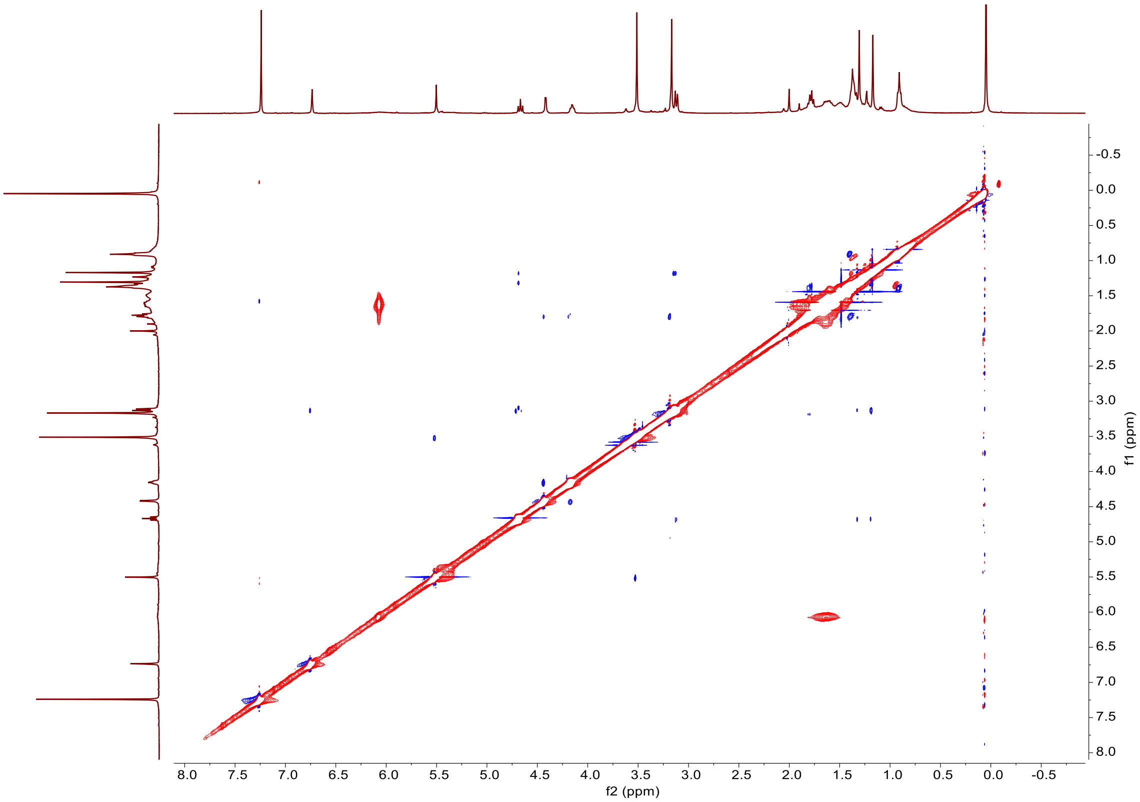

Figure S57. NOESY spectrum of **5** in $CDCl_3$.

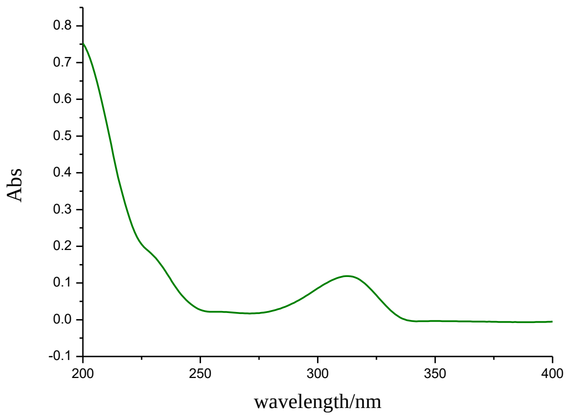

Figure S58. UV spectrum of **6** in MeOH.

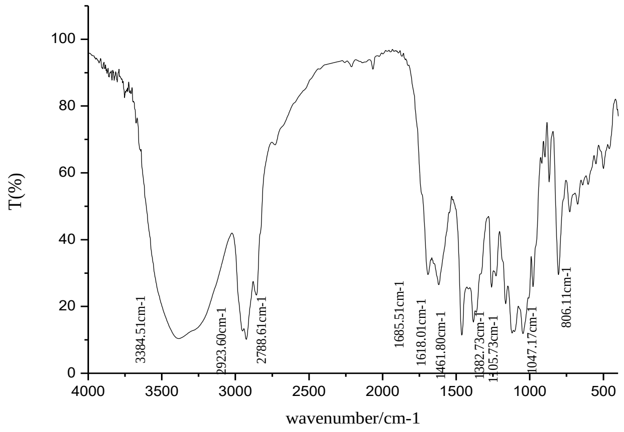

Figure S59. IR spectrum of **6** (KBr disc).

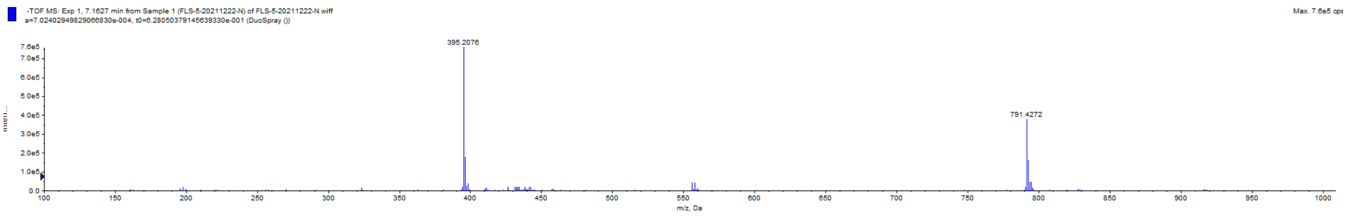

Figure S60. HR-ESI-MS of **6**.

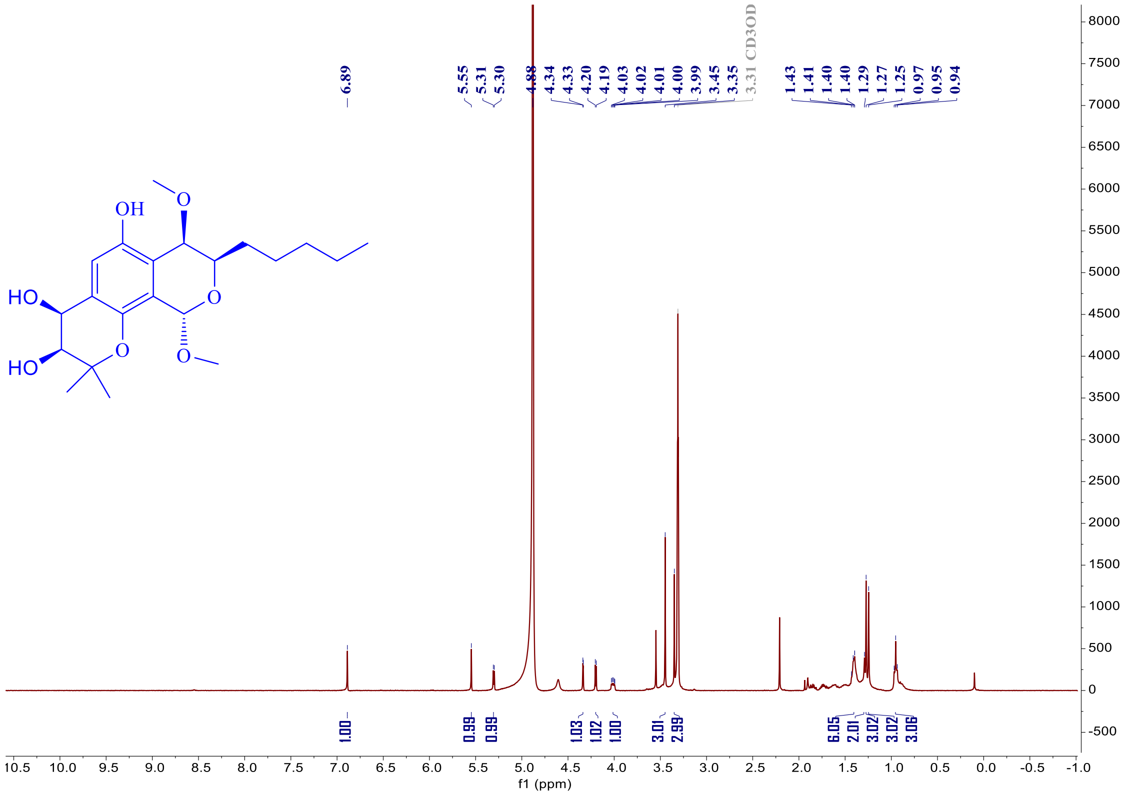

Figure S61. ^{1}H NMR spectrum of **6** in CD_3OD.

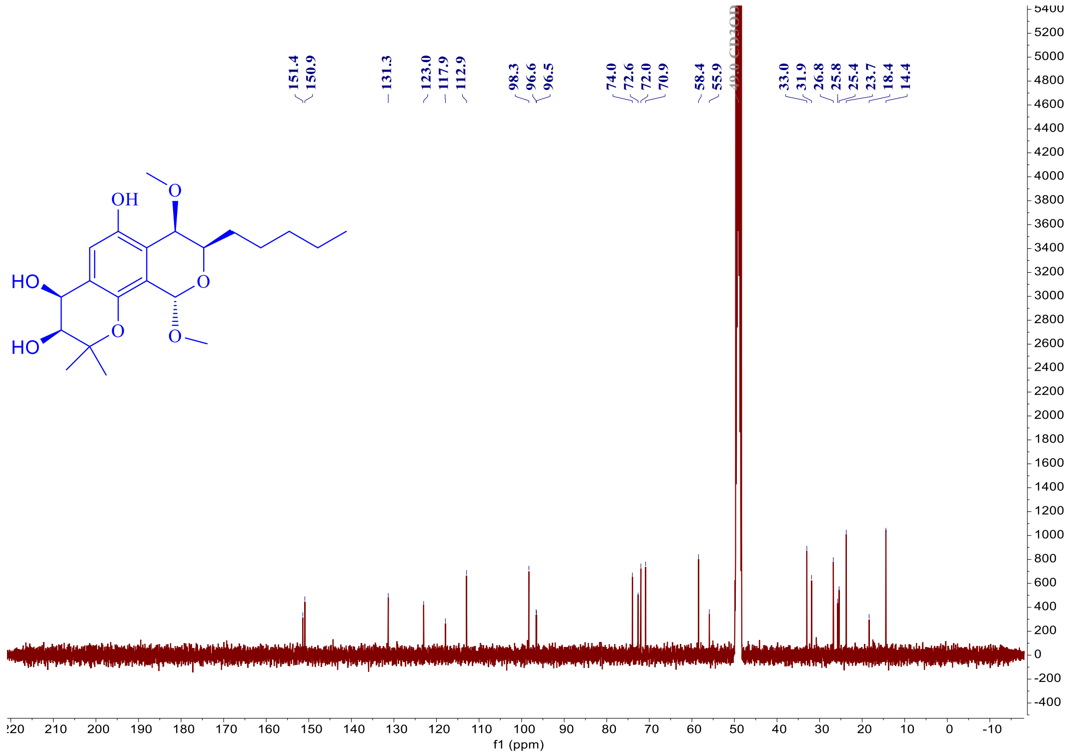

Figure S62. ^{13}C NMR spectrum of **6** in CD_3OD.

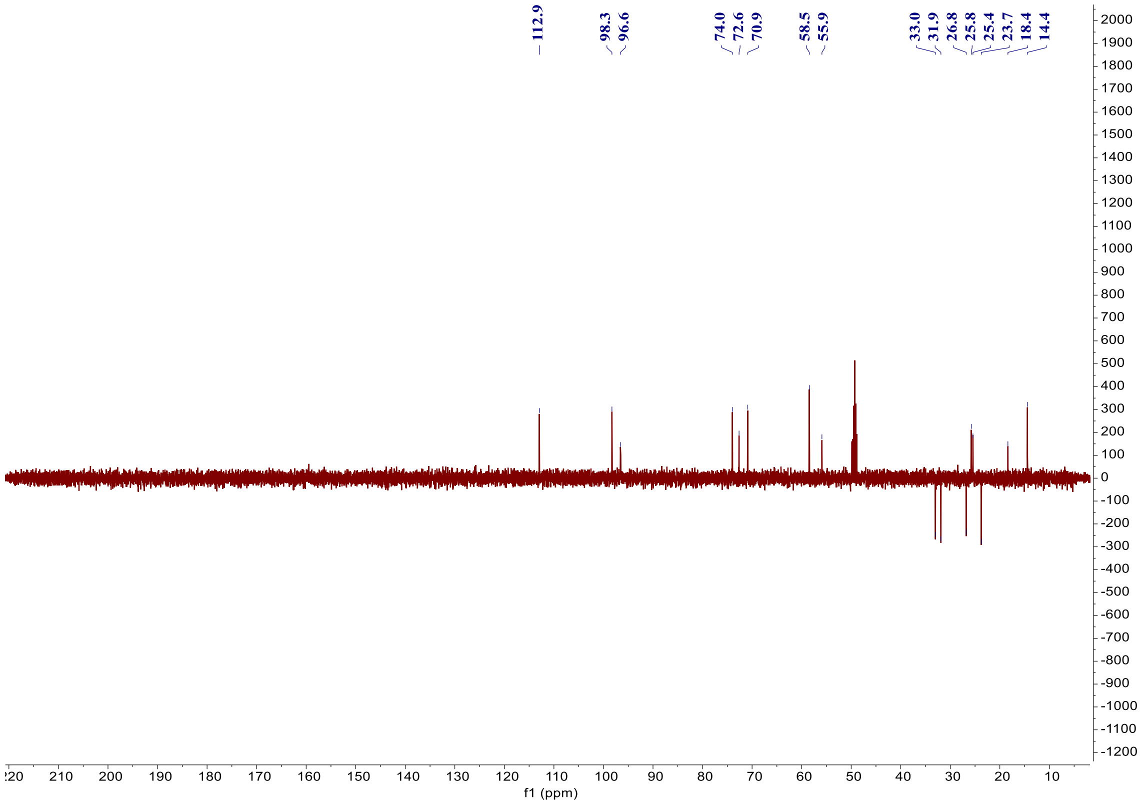

Figure S63. DEPT 135 spectrum of **6** in CD_3OD.

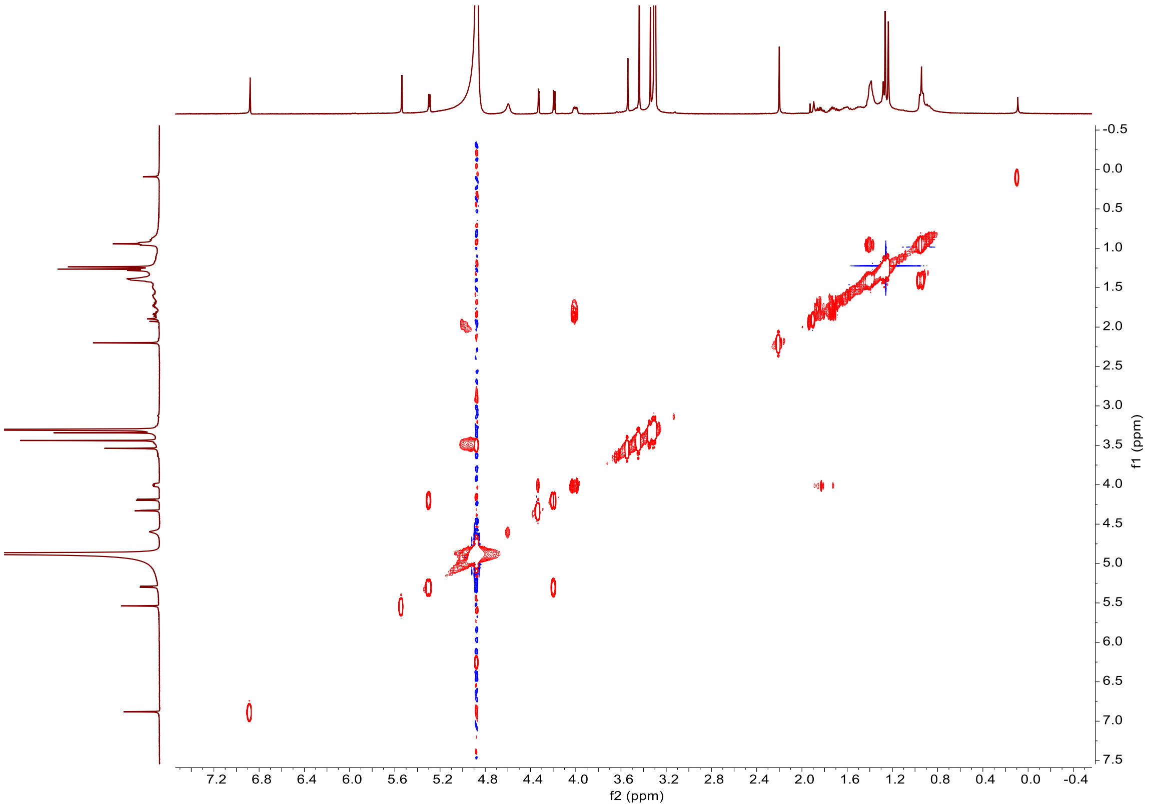

Figure S64. $^1H-^1H$ COSY spectrum of **6** in CD_3OD.

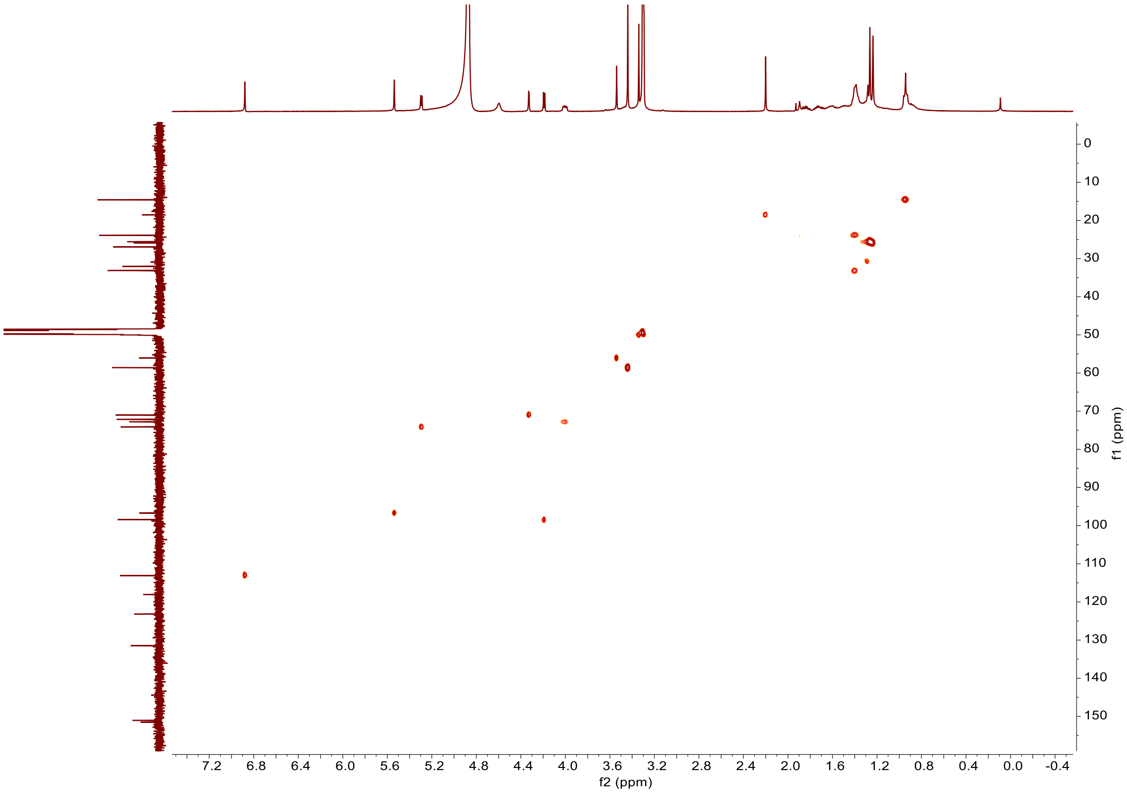

Figure S65. HSQC spectrum of **6** in CD_3OD.

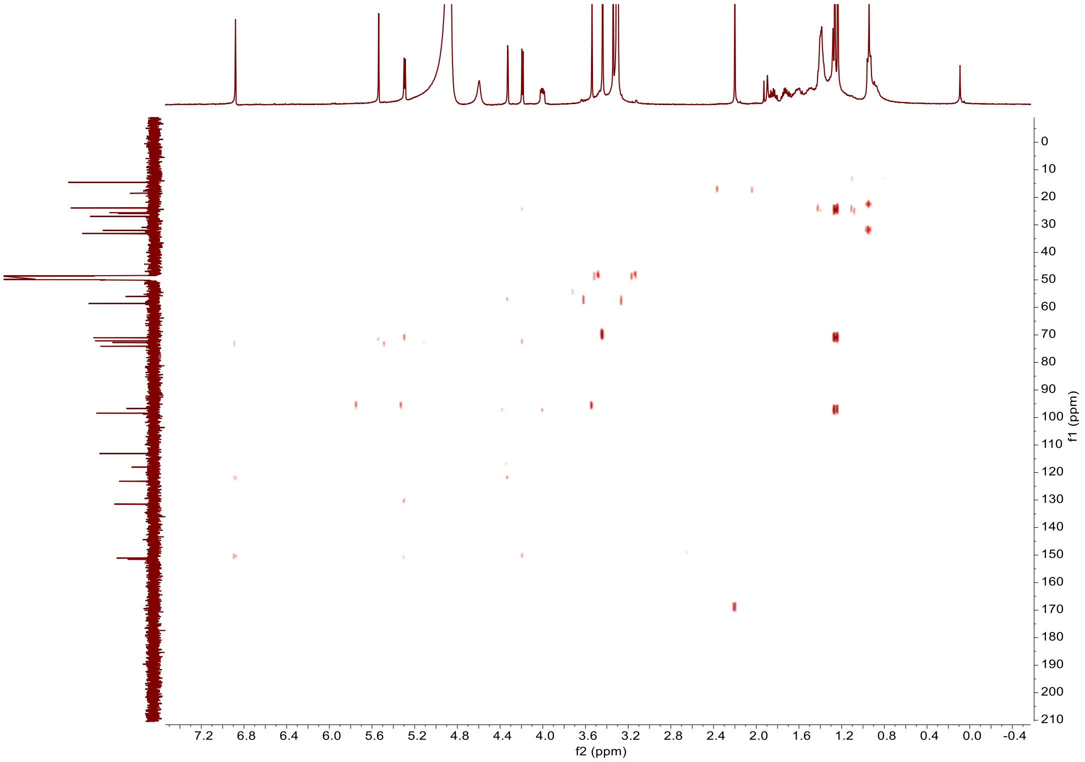

Figure S66. HMBC spectrum of **6** in CD_3OD.

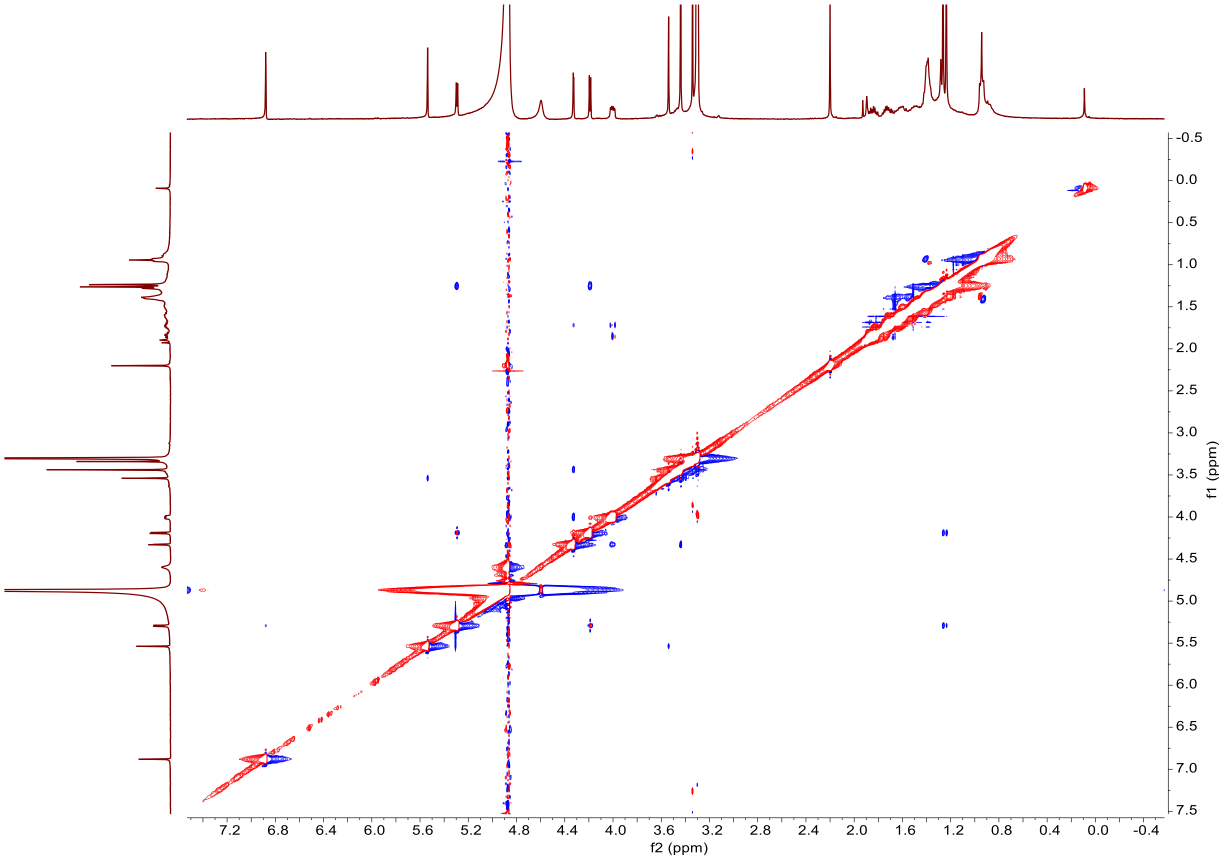

Figure S67. NOESY spectrum of **6** in CD_3OD.
